# Problem‐oriented policing for reducing crime and disorder: An updated systematic review and meta‐analysis

**DOI:** 10.1002/cl2.1089

**Published:** 2020-06-15

**Authors:** Joshua C. Hinkle, David Weisburd, Cody W. Telep, Kevin Petersen

**Affiliations:** ^1^ Department of Criminal Justice and Criminology Georgia State University Atlanta Georgia; ^2^ Criminology, Law and Society George Mason University Fairfax Virginia; ^3^ Institute of Criminology, Faculty of Law Hebrew University Jerusalem Israel; ^4^ School of Criminology and Criminal Justice Arizona State University Phoenix Arizona

## Abstract

**Background:**

Herman Goldstein developed problem‐oriented policing (POP) to focus police on more proactively addressing chronic problems, rather than using traditional reactive efforts. POP has been utilized to target a wide range of problems and has become commonly used in agencies across the United States and the world, although implementation is often uneven. POP interventions commonly use the SARA (scanning, analysis, response, assessment) model to identify problems, carefully analyze the conditions contributing to the problem, develop a tailored response to target these underlying factors, and evaluate outcome effectiveness.

**Objectives:**

To extend and update the findings of the original POP systematic review by synthesizing the findings of published and unpublished evaluations of POP through December 2018 to assess its overall impacts on crime and disorder. The review also examined impacts of POP on crime displacement, police financial costs, and noncrime outcomes.

**Search Methods:**

Searches using POP keywords of the Global Policing Database at the University of Queensland were conducted to identify published and unpublished evaluations between 2006 and 2018. We supplemented these searches with forward searches, hand searches of leading journals and the Center for Problem‐Oriented Policing, and consultation with experts.

**Selection Criteria:**

Eligible studies had to include a target area or group that received a POP intervention AND a control area/group that received standard police services. The control condition could be either experimental or quasi‐experimental. Units of analysis could be places or people. We defined POP as studies that generally followed the tenets of the SARA model.

**Data Collection and Analysis:**

We identified 39 new (published between 2006 and 2018) studies that met our eligibility criteria as an evaluation of POP. Twenty‐four of these studies had sufficient data available to calculate an effect size. Along with the 10 studies from our initial systematic review of POP, these 34 studies are included in our meta‐analytic review of POP. Nine of these studies were randomized experiments and 25 were quasi‐experiments. We calculated effect sizes for each study using Cohen's *D* and relative incidence risk ratios and used random effects meta‐analyses to synthesize studies.

**Results:**

Our meta‐analyses suggest statistically significant impacts of POP. Our relative incident risk ratio analysis of mean effects suggests a 33.8% reduction in crime/disorder in the POP treatment areas/groups relative to the controls. We find no evidence of significant crime displacement as a result of POP and some evidence for a greater likelihood of a diffusion of crime control benefits. Few studies assessed noncrime outcomes, but our narrative review suggests POP is cost‐effective, but has limited impacts on fear of crime, legitimacy, and collective efficacy.

**Authors’ Conclusions:**

Our review provides strong and consistent evidence that POP is an effective strategy for reducing crime and disorder. There is a great deal of heterogeneity in the magnitude of effect sizes across factors such as study type, study rigor and crime type. Despite this heterogeneity, 31 out of 34 studies (91.2%) have effect sizes in favor of a treatment effect and the overall mean effect is positive and significant in all of our models.

## 1 PLAIN LANGUAGE SUMMARY

### 1.1 Problem‐oriented policing (POP) is associated with reductions in crime and disorder

POP is associated with statistically significant reductions in crime and disorder. Place‐based POP programs are more likely to produce a diffusion of benefits into areas adjacent to targeted locations than to lead to crime displacement.

### 1.2 What is this review about?

POP is a proactive policing strategy developed by Herman Goldstein, who argued that the standard reactive model of policing was ineffective as it was overly focused on the means of policing (number of arrests, average response time, etc.) rather than the end goal of reducing crime and enhancing community safety. He suggested that police could be more effective if they were more proactive and researched root causes of crime, and developed tailor‐made responses.

This review assesses the effectiveness of POP interventions—defined as those programs which generally followed the tenets of the SARA model (scanning, analysis, response, assessment) developed by Spelman and Eck—in reducing crime and disorder and fear of crime, and improving citizen perceptions of police.

**What is the aim of this review?**

This update of a Campbell systematic review assesses the effectiveness of problem‐oriented policing in reducing crime and disorder. It summarises the evidence from 34 studies: 28 from the United States, five from the United Kingdom, and one from Canada.

### 1.3 What studies are included?

This review includes both randomized and quasi‐experimental evaluations of POP, where a treatment area or group received a POP approach while a control area or group received standard police services.

Thirty‐four studies are assessed in the review—an increase of 24 studies from the original review (Weisburd, Telep, Hinkle, & Eck, 2008; Weisburd, Telep, Hinkle, & Eck, 2010). All studies were published between 1989 and 2018. Most studies (28) were conducted in the United States, five in the United Kingdom, and one in Canada.

### 1.4 Does POP reduce crime and disorder?

Yes. The results of this updated systematic review suggest that POP is associated with a statistically significant overall reduction in crime and disorder of 34%.

There are positive impacts for POP across a wide variety of crime and disorder outcomes, among studies that targeted problem places and problem people, at a variety of different units of analysis and featuring a wide array of types of interventions. The effect size is smaller in randomized experiments and after accounting for publication bias.

POP had limited impacts on police legitimacy, fear of crime, and collective efficacy. Few studies incorporated cost‐benefit analyses, but those that did suggest POP can be cost‐effective and provide substantial savings through prevented calls‐for‐service and incidents.

### 1.5 What do the findings of the review mean?

Findings from this review support the notion that proactive policing strategies that identify specific problems, conduct analyses to determine underlying causes, and develop and deliver tailor‐made responses, are more effective in reducing crime and disorder than standard, reactive methods of policing. Moreover, in place‐based interventions, diffusion of crime‐reduction benefits are more likely than displacing crime to nearby areas. As such, police departments should incorporate the use of problem‐solving into their crime prevention strategies.

However, the impacts of POP on crime are highly heterogeneous. This result may reflect the tremendous variability in the types of problems identified and targeted and the types of tailored intervention strategies used, suggesting that more studies are needed to allow more robust analyses of factors that influence POP program impacts. In turn, future evaluations should be designed to capture more data about the problem‐solving process so that future reviews can more directly assess what types of problems seem most amenable to POP efforts and what characteristics of problem‐solving interventions are associated with larger effects.

### 1.6 How up‐to‐date is this review?

This authors of this review update searched for studies up to December 2018.

## 2 BACKGROUND

### 2.1 The issue

In an article in *Crime & Delinquency* in 1979, Herman Goldstein critiqued police practices of the time by noting that they were more focused on the “means” of policing than the “ends” or goals of policing. His critique drew from a series of recently completed studies that suggested that such standard policing practices as “preventive patrol” (Kelling, Pate, Dieckman, & Brown, 1974) or “rapid patrol car response to calls for service” (Kansas City Police Department, 1977) had little impact on crime. Goldstein suggested that the research evidence was not idiosyncratic but reflected a crisis in policing. To illustrate his concern, he referred to a newspaper article in the United Kingdom that reported on bus drivers in a small city that were passing bus stops waving and smiling but failing to pick up passengers. When questioned by a reporter, a representative for the bus company responded that “it is impossible for the drivers to keep their timetable if they have to stop for passengers” (Goldstein, 1979, p. 236). Goldstein termed this the “means over ends syndrome” and noted that police were particularly susceptible to this problem. Goldstein noted that the police too had become so focused on the means of policing—such issues as the staffing and management of police—that they had begun to ignore the problems policing was meant to solve. Goldstein saw this dysfunction as at the heart of the failures of the police to be effective in addressing community problems.

Goldstein called for a paradigm shift in policing that would replace the primarily reactive, incident driven “standard model of policing” (Skogan & Frydl, 2004; Weisburd & Eck, 2004) with a model that required the police to be proactive in identifying underlying problems that could be targeted to alleviate crime and disorder at their roots. He termed this new approach “problem‐oriented policing” to accentuate its call for police to focus on problems and not on the everyday management of police agencies. Goldstein also expanded the traditional mandate of policing beyond crime and law enforcement. He argued that the police should deal with an array of problems in the community, including not only crime, but also social and physical disorder.

He also called for police to expand the tools of policing much beyond the law enforcement powers that are seen as the predominant tools of the standard model of policing. In Goldstein's view, the police needed to draw upon not only the criminal law, but also civil statutes and rely on other municipal and community resources if they were to successfully ameliorate crime and disorder problems. As such, successful implementations of POP would be reliant on forming partnerships with other agencies, community organizations and community members to deliver non‐law enforcement responses. This would particularly be the case when the targeted problems do not necessarily involve law violations.

John Eck and William Spelman (1987) drew upon Goldstein's idea to create a straightforward model for implementing POP, which has become widely accepted. In an application of problem solving in Newport News, in which Goldstein acted as a consultant, they developed the SARA model for problem solving. SARA is an acronym representing four steps they suggest police should follow when implementing POP, which will be outlined in Section 2.2.^1^

A 2004 report from the National Research Council offered the following description of POP and how the SARA model works in practice:

The heart of problem‐oriented policing is that this concept calls on police to analyze problems, which can include learning more about victims as well as offenders, and to consider carefully why they came together where they did. The interconnectedness of person, place, and seemingly unrelated events needs to be examined and documented. Then police are to craft responses that may go beyond traditional police practices … Finally, problem‐oriented policing calls for police to assess how well they are doing. Did it work? *What* worked, exactly? Did the project fail because they had the wrong idea, or did they have a good idea but fail to implement it properly? (Skogan & Frydl, 2004, p. 91).

A number of studies going back to the mid‐1980s demonstrate that problem solving can be utilized to address a variety of police concerns, including fear of crime (Cordner, 1986), violent and property crime (Eck & Spelman, 1987), firearm‐related youth homicide (Kennedy, Braga, Piehl, & Waring., 2001), and various forms of disorder, including prostitution and drug dealing (Capowich & Roehl, 1994; Eck & Spelman, 1987; Hope, 1994). As a further example of the proliferation of POP, the Center for Problem‐Oriented Policing (POP Center, https://popcenter.asu.edu/) documents a large number of case studies and evaluations of POP. For instance, there have been over 1,000 programs submitted for consideration for the Goldstein Award and more than 800 submissions to the Tilley Award. These submissions document the use of a wide array of problem‐solving responses to document crime, disorder and a host of other issues police are tasked with addressing, highlighting the utility of the POP model for a wide variety of problem types (see also, Scott, 2000; Scott & Clarke, 2020). As our review is focused on impacts on crime and disorder, we limit our discussion here to those outcomes.

There are also a number of experimental and other more rigorous examinations of POP. For example, a study in Jersey City, New Jersey, public housing complexes (Mazerolle, Ready, Terrill & Waring, 2000) found that a police problem‐solving model could be used to respond to violent and property crime in six housing complexes. In another example, Clarke and Goldstein (2002) report a POP project to reduce thefts of appliances from new home construction in Charlotte, North Carolina. Officers carefully analyzed this problem before working with construction firms to implement changes in building practices.

Two early experimental evaluations of applications of problem solving in crime hot spots (Braga et al., 1999; Weisburd & Green, 1995) suggested POP interventions, particular those implemented in crime hot spots, could be evaluated rigorously.^2^ In a randomized trial involving Jersey City violent crime hot spots, Braga et al. (1999) examined the impact of problem solving in 12 hot spots on property and violent crime. While this study tested problem‐solving approaches, it is important to note that focused police attention was brought only to the experimental locations. Accordingly, it is difficult to distinguish between the effects of bringing focused attention to hot spots and that of such focused efforts being developed using a problem‐oriented approach. The Jersey City Drug Market Analysis Experiment (Weisburd & Green, 1995) more directly tested the value added of problem‐solving approaches in hot spots policing. In that study, a similar number of narcotics detectives were assigned to treatment and control hot spots. Weisburd and Green (1995) compared the effectiveness of unsystematic, arrest‐oriented enforcement based on ad hoc target selection (the control group) with a treatment strategy involving analysis of assigned drug hot spots, followed by site‐specific enforcement and collaboration with landlords and local government regulatory agencies, and concluding with monitoring and maintenance for up to a week following the intervention. More recent experimental evaluations have also examined the impact of POP in crime hot spots (e.g., Braga & Bond, 2008; Groff et al., 2015; Taylor, Koper, & Woods, 2011).

In sum, POP has emerged as one of the most widely accepted and widely used strategies in American policing (Scott, 2000; Weisburd & Majmundar, 2018). This is indicated both by the adoption of POP by major federal agencies and national policing groups, the creation of national awards for effective POP programs, and the widespread adoption of the approach in American policing and throughout the world. For example, the U.S. federal agency, the Office of Community Oriented Policing Services (COPS) adopted POP as a key strategy, initially funding the Center for Problem‐Oriented Policing, which has developed over 70 problem‐specific guides for police. More recently, the Bureau of Justice Assistance has also funded the creation of problem‐oriented response and tool guides. The Police Executive Research Forum adopted POP as a “powerful tool in the policing arsenal,” in the 1980s and began to run a yearly national conference to promulgate and advance POP strategies (Solé Brito & Allan, 1999, p. xiii) that the POP Center still continues today. In 1993 the Herman Goldstein Award was created for “problem solving excellence.” In the United Kingdom, the Tilley Award for POP was created in 1999.^3^ To date there have been more than 1,800 submissions to these awards. Reflecting the wide‐scale adoption of POP by American police agencies, the 2013 Law Enforcement Management and Administrative Statistics (LEMAS) survey reported that 33% of all departments, and 74% of departments serving 100,000–249,000 citizens, reported actively encouraging officer involvement in problem‐solving projects (Reaves, 2015).^4^

While POP has been widely adopted and assessed, it is important to note that fully implementing POP has been challenging (Cordner & Biebel, 2005; Maguire, Uchida, & Hassell, 2015), and programs are often characterized by partial implementations of the SARA model that have been termed “shallow” problem‐solving (Braga & Weisburd, 2010). For instance, in his Stockholm lecture Goldstein (2018) noted that many early initiatives lacked fundamental understanding of the POP approach and that he did not adequately acknowledge the importance of having enough individuals with the requisite research and assessment skills when developing his model. However, he also noted that he has been impressed by improvements in areas such as focusing specifically on micro‐problems, the engagement of rank‐and‐file officers in problem solving, the use of a broad range of responses, and increasing engagement of the private sector in partnerships to share responsibility for public safety problems. Thus while POP still has a long way to go to be fully embedded in police agencies, much less to become standard police practice as Goldstein hoped, there is reason to believe that the model is both spreading and improving in quality over time.

### 2.2 POP in practice

Since its initial proposition, the POP model has been further articulated by Eck and Spelman (1987) whose work in Newport News produced the SARA model. SARA is an acronym representing four steps they suggest police should follow when implementing POP. “Scanning” is the first step, and involves the police identifying and prioritizing potential problems in their jurisdiction that may be causing crime and disorder. After potential problems have been identified, the next step is “Analysis.” This involves the police analyzing the identified problem(s) in‐depth using a variety of data sources so that appropriate responses can be developed. The third step, “Response,” has the police developing and implementing interventions tailored to what was learned in the “analysis” step and designed to solve the problem(s). The search for responses should be broad and not limited to law enforcement methods, and often should involve partnering with other agencies, community groups and/or community members depending on the type of problem and its causes. Indeed, the POP model stresses the need to shift and share responsibility for public safety, and this will require police to identify and mobilize partners (Goldstein, 2018; Scott & Goldstein, 2005). Finally, once the response has been administered, the final step is “Assessment” which involves assessing the impact of the response on the targeted problem(s).

For example, a police agency may determine that drug‐related crime is on the rise in their jurisdiction, constituting a problem in need of prioritization (Scanning phase). Further examination of the nature of drug‐related crime may reveal problem areas and times (Analysis phase). Based on this analysis, the agency may choose to direct increased patrol and enforcement to the specific areas deemed problematic at the specific times deemed problematic and to partner with community organizations to deliver substance abuse treatment programs (Response phase). After a period of time the agency may compare drug‐related crime in the jurisdiction as a whole, as well as in the targeted areas, from before and after the response was implemented (Assessment phase).

This process in general, rather than the specific problem or response chosen, represents the core concept of POP. Thus, a diverse set of variations in problems, responses, and length of interventions are possible across an array of targets (i.e., problem places of varying size or problem people may be the focus) and virtually any unit of analysis.

### 2.3 How might POP work?

It is hypothesized that POP affects change in problem outcomes through an increased knowledge of, and responsiveness to, the mechanisms through which a particular problem operates. The National Academies of Sciences Committee on Proactive Policing noted in its consensus report:

Problem‐oriented policing is an analytic method for developing crime reduction tactics. This strategy draws upon theories of criminal opportunity, such as rational choice and routine activities, to analyze crime problems and develop appropriate responses (Braga, 2008; Clarke, 1997; Reisig, 2010). Using a basic iterative process of problem identification, analysis, response, assessment, and adjustment of the response (often called the scanning, analysis, response, and assessment [SARA] model), this adaptable and dynamic analytic method provides a framework for uncovering the complex mechanisms at play in crime problems and for developing tailor‐made interventions to address the underlying conditions that cause crime problems (Eck & Spelman, 1987; Goldstein, 1990). Depending on the nuances of particular problems, the responses that are developed—even for seemingly similar problems—can be diverse. Indeed, problem‐oriented policing interventions draw upon a variety of tactics and practices, ranging from arrest of offenders and modification of the physical environment to engagement with community members (Weisburd & Majmundar, 2018, p. 53).

POP is not concerned simply with the problem outcomes themselves but rather with the underlying processes that lead to problems emerging and developing. Addressing the underlying mechanisms that cause problems should lead to long‐term solutions and should lead police agencies to think and act in ways that go beyond their normal day‐to‐day operations. Furthermore, the assessment of results should lead to refinement and improvement in subsequent efforts.

Moreover, while the POP model does not favor any particular kind of intervention, one can still look beyond this literature and find support for the basic ideas behind the models by examining evidence for approaches commonly utilized in POP programs. For instance, evidence of the effectiveness of situational and opportunity‐blocking strategies, while not necessarily police based, provides indirect support for the effectiveness of problem solving in reducing crime and disorder. Moreover, POP has been linked to routine activity theory, rational choice perspectives, and situational crime prevention (Clarke, 1992a, 1992b; Eck & Spelman, 1987). Reviews of prevention programs designed to block crime and disorder opportunities in small places find that most of the studies report reductions in target crime and disorder events (Eck, 2002; Poyner, 1981; Weisburd & Telep, 2014; Weisburd, 1997),^5^ and many of these efforts were the result of police problem‐solving strategies. Further, a systematic review and meta‐analysis of situational crime prevention both supports its effectiveness and that such approaches do not merely displace crime to other areas (Guerette & Bowers, 2009). Lastly, hot spots policing and focused deterrence approaches that involve problem‐solving have been found effective in recent systematic reviews (Braga, Turchan, Papachristos, & Hureau, 2019; Braga, Weisburd, & Turchan, 2019).

### 2.4 Why is it important to do this review?

This is an update to an earlier Campbell systematic review of the effectiveness of POP that included studies through 2006 and identified a total of 10 studies that met the Campbell criterion for inclusion—4 randomized experiments and 6 quasi‐experiments (Weisburd et al., 2008, 2010). Overall, the findings of this review largely reinforced those of prior narrative reviews and more general assumptions of the effectiveness of POP. Specifically, the authors noted “[w]hether we used a more conservative mean effect size approach or examined the largest effects on crime and disorder reported, we found that POP approaches have a statistically significant effect on the outcomes examined. Importantly, the results are similar whether we look at experimental or nonexperimental studies” (Weisburd et al., 2010, p. 162).

However, the original review also noted that effect sizes were relatively modest, ranging between 0.10 and 0.20 (measured as Cohen's *D*) and were based upon only 10 experimental or quasi‐experimental studies. As such, an updated review may help to shed further light on the ability of POP to reduce crime and disorder problems by analyzing an increased base of empirical research on POP interventions by including studies up to the end of 2018 (12 years beyond the cutoff for the original review's search). Having more complete and current evidence on POP is especially important given an increasing focus on problem‐solving and other proactive policing approaches around the world (Weisburd & Majmundar, 2008).

We also add an additional approach to measuring effect sizes suggested by Wilson (in progress) that has statistical properties better suited to the nature of place‐based data and provides a more easily interpretable set of estimates of program outcomes. As empirical knowledge on POP's effectiveness increases, police agencies may be able to better determine ways to identify and respond to the various problems occurring in their jurisdictions.

## 3 OBJECTIVES

The objectives of this updated review are to extend the findings of the original review (Weisburd et al., 2008, 2010) by synthesizing the findings of published and unpublished evaluations of POP through December 2018 to assess its overall impacts on crime and disorder. Spatial displacement was also assessed for studies that provided data needed to calculate effect sizes for such effects. Finally, while too few studies included outcomes other than crime or disorder to allow for meaningful meta‐analyses, impacts on items such as police legitimacy and fear of crime are reviewed narratively, as well as findings about the financial cost/benefits of POP.

## 4 METHODS

### 4.1 Criteria for considering studies for this review

#### 4.1.1 Types of studies

For studies to be considered in this review the evaluation had to include a target area or group that received a POP intervention AND a control area/group that received standard police services. The control condition could be either experimental or quasi‐experimental (Campbell & Stanley, 1966; Cook & Campbell, 1979; Shadish, Cook, & Campbell, 2002).

The following research designs were eligible for inclusion in our review (this is adapted from the inclusion criterion in Global Policing Database protocol [Higginson, Eggins, Mazerolle, & Stanko, 2015, pp. 47–48]):

- Randomized experimental designs (RCTs)
- The following “strong” quasi‐experimental designs:
  ◦ Regression discontinuity designs
  ◦ Matched control group designs with or without preintervention baseline measures (propensity or statistically matched)
  ◦ Unmatched control group designs with preintervention measures (difference‐in‐difference analysis)
  ◦ Short interrupted time‐series designs with control group (less than 25 pre and 25 postintervention observations [Glass, 1997])
  ◦ Long interrupted time‐series designs with control group (≥25 pre‐ and postintervention observations ([Glass, 1997])

- The following “weak” quasi‐experimental designs:
  ◦ Unmatched control group designs with pre–postintervention measures which allow for difference‐in‐difference analyses
  ◦ Unmatched control group designs without preintervention measures where the control group has face validity
  ◦ Raw unadjusted correlational designs where the variation in the level of the intervention is compared with the variation in the level of the outcome
  ◦ Treatment‐Treatment Designs

Unlike some Campbell reviews, we included studies with nonequivalent control groups; for example, studies that compared a target area to the rest of the jurisdiction. As the POP model requires police to identify specific problems in specific areas or populations, it will often be difficult for evaluators to create equivalent comparison areas/groups (Eck, 2006a). As such, we did not restrict our review to quasi‐experiments with equivalent control groups as we felt it important to be inclusive of studies that were representative of how POP is often carried out in practice. Thus any evaluation of POP that included a comparison group that did not receive the POP intervention was eligible for our review if it met our other inclusion criteria

#### 4.1.2 Type of areas/groups

As noted above, POP is a general approach that calls for police to identify specific problems and develop specific responses to them based on potential underlying causes determined through problem analysis. As such, POP is not limited to any specific unit of analysis. For example, problems can be citywide, confined to small areas such as hot spots or can be individual offenders or groups of offenders rather than places. As such our review is not restricted by the type of target and includes problems at any unit of analysis that were addressed with a POP intervention.

#### 4.1.3 Types of interventions

Given that the POP model calls for police to develop tailor‐made responses designed to address underlying causes of identified problems, a nearly limitless array of interventions can be associated with the approach. As such, our review is not restricted to any specific type of police response to crime or disorder problems. In this review we treat the use of the SARA model described above to identify problems, research underlying causes and develop and deliver specific responses to address them as the “intervention.” That is to say that our central question is whether using the SARA model to identify and respond to problems is associated with larger crime reduction compared with traditional reactive policing strategies. Further, we did not require that publications specifically mention the SARA steps (or even POP). We carefully read every potentially eligible study identified through our search and included studies if we could determine the interventions roughly followed the tenets of the SARA model.^6^

#### 4.1.4 Types of outcome measures

The primary outcomes examined in this review, and included in our meta‐analyses, are measures of crime and disorder. By far the most commonly used measures of these outcomes in evaluations of POP are police recorded calls for service or incident reports. However, all measures of crime and disorder such as arrests, social observations or resident perceptions were coded. We also coded survey measures of other outcomes such as citizen perceptions/opinions of police, fear of crime, and collective efficacy where possible. We had hoped to get enough of these types of measures to conduct meaningful meta‐analyses on some of these types of outcomes. However, few studies reported on more than crime/disorder outcomes, and those that did are characterized by wide variation in measures used and data reported in study publications. As such we provide a narrative review and summary of the limited findings for such outcomes, as well as cost‐benefit analyses. We also conducted a meta‐analysis of displacement/diffusion effects, and the narrative summaries in Appendix A also discuss conclusions about these effects drawn in studies that did not provide data needed to calculate these effect sizes.

### 4.2 Search strategy for identification of studies

The search for this updated review was led by the Global Policing Database (GPD) research team at the University of Queensland (Elizabeth Eggins and Lorraine Mazerolle) and Queensland University of Technology (Angela Higginson). The University of Queensland is home to the GPD (see http://www.gpd.uq.edu.au), which served as the main search location for this review. The GPD is a web‐based and searchable database designed to capture all published and unpublished experimental and quasi‐experimental evaluations of policing interventions conducted since 1950. There are no restrictions on the type of policing technique, type of outcome measure or language of the research (Higginson et al., 2015). The GPD is compiled using systematic search and screening techniques, which are reported in Higginson et al. (2015) and summarized in detail in Appendices B and C. Broadly, the GPD search protocol includes an extensive range of search locations to ensure that both published and unpublished research is captured across criminology and allied disciplines.

To capture studies, we used POP terms to search the GPD corpus of full‐text documents that have been screened as reporting on a quantitative impact evaluation of a policing intervention. Specifically, we used the following terms to search the title and abstract fields of the corpus of documents published from January 2006 through to December 2018:

- "problem‐orient*”
- "problem orient*"
- “problem solv*”
- SARA
- scan*
- "problem focus*"
- “problem ident*”
- “ident* problem*”
- “situational crime prevent*”
- POP

Several additional strategies were also used to extend the GPD search. First, we performed forward citation searches for works that have cited seminal POP studies.^7^ Second, we conducted hand searches of 2017 and 2018 volumes of leading journals in the field to identify any recent studies that may have not yet been indexed in the GPD.^8^ Third, we reviewed the Center for Problem‐Oriented Policing website for all Tilley Award and Goldstein Award winners and submissions.^9^ Fourth, after finishing the above searches and reviewing the studies as described later, we e‐mailed the list to leading policing scholars knowledgeable in the area of POP (see list in Appendix D). This was aimed at identifying studies the above searches missed, as these experts may be able to refer us to eligible studies missing from our list, particularly unpublished pieces such as dissertations and smaller research reports.

Several strategies were used to obtain full‐text versions of the studies found through our search. First, we attempted to obtain full‐text versions from the electronic journals available through the George Mason University, Georgia State University, and Arizona State University libraries. When electronic versions were not available, we used print versions of journals available at the library. If the journals were not available, we made use of both the GPD team and the Interlibrary Loan Office (ILL) to obtain the journal from the libraries of other area schools. When those methods did not work, we contacted the author(s) of the article and/or the agency that conducted and/or funded the research to try to get a copy of the full‐text version of the study.

### 4.3 Data collection and analysis

Search results were given title and abstract review by Kevin Petersen, one of the authors of this review. Any studies that were not obviously eligible or ineligible were flagged. Flagged studies were reviewed by the other three authors of this review, who then discussed and voted on each study's eligibility. All inclusion/exclusion decisions were unanimous.

#### 4.3.1 Details of study coding categories

All eligible studies were coded on a variety of criteria including (but not limited to):

a. Reference information (title, authors, publication, etc.)
b. Nature of description of selection of site, problems, etc.
c. Nature and description of selection of comparison group or period
d. The unit of analysis
e. The sample size
f. Methodological type (randomized experiment, quasi‐experiment, or pre–post test)
g. A description of the POP intervention
h. Dosage intensity and type
i. Implementation difficulties
j. The statistical test(s) used
k. Reports of statistical significance (if any)
l. Effect size/power (if any)
m. Cost‐benefit analysis (if applicable)
n. The conclusions drawn by the authors

The full coding sheet is provided in Appendix E. Kevin Petersen (one of the authors of the review) and another graduate research assistant at George Mason University independently coded each eligible study. Where there were discrepancies, Drs. Hinkle, Weisburd and Telep reviewed the study, had discussions and voted to determine the final coding decision.

#### 4.3.2 Statistical procedure and conventions

We completed a meta‐analysis of the 34 eligible studies by calculating a standardized effect size for each included outcome and then estimating an overall random effect for the impact of POP on crime and disorder. We used Biostat's Comprehensive Meta Analysis 3.0 program for our analyses and to create the forest plots we present below.

Computation of effect sizes in the studies was not always direct. The goal was to convert all observed effects into a standardized mean difference effect size metric. None of the studies we examined calculated standardized effect sizes, and indeed, it was sometimes difficult to develop precise effect size metrics from published materials. This reflects a more general problem in crime and justice with “reporting validity” (Farrington, 2006; Lösel & Köferl, 1989), and has been documented in recent reviews of reporting validity in crime and justice studies (see Perry & Johnson, 2008; Perry, Weisburd, & Hewitt, 2010).

For many of our eligible studies, effect sizes could only be calculated using pre‐ and postintervention crime/disorder counts for the treatment and control group/area. A similar approach was used for some studies in our earlier review and is common in systematic reviews of policing interventions (e.g., Braga and colleagues’ [2019] recent update of their Campbell review of the effectiveness of hot spots policing).

This approach involves calculating relative incidence rate ratio (RIRR), and the variance of the log RIRR from the raw counts using the following formulae (the table provides an example of the grid of pre and post counts used for these equations):

**Table:** 

	Pre	Post
Treatment	*a*	*b*
Control	*c*	*d*


RIRR=(a×d)/(b×c).

V(logRIRR)=(1/a)+(1/b)+(1/c)+(1/d).

The variance of the log of the RIRR (V(log RIRR)) was adjusted for over‐dispersion using the approach outlined by Farrington, Gill, Waples and Argomaniz (2007).^10^ This adjustment is calculated as the product of V(log RIRR) and *D*, with *D* = 0.0008 × *N* + 1.2. *N* is indexed as the mean number of incidents per case and is calculated as the total number of incidents (*a* + *b* + *c* + *d*) divided by the total number of treatment plus control areas/groups.

Finally, Cohen's *D* is obtained by multiplying the log of RIRR by √3/*π*, while its standard error is calculated by multiplying the adjusted V(log RIRR) by (3/*π* ^2^; Hasselblad & Hedges, 1995).

While the Cohen's *D* approach allows us to compare our findings to the prior review, Wilson (in progress) argues that the Cohen's *D* approach fails to produce effect sizes that are comparable across studies when based on place‐based count data (the majority of studies in our review). Moreover, he has also pointed out that Cohen's *D*s obtained through the above conversion are not comparable to those calculated directly through conventional means. As such, we also present meta‐analysis models where the effect size is the log RIRR and its standard error (which is the square root of adjusted V(log RIRR)).^11^ This approach also has an advantage in that the exponent of the log RIRR can be interpreted simply as the relative percent change in the treatment group compared with the control group.

#### 4.3.3 Determination of independent findings

We first note that a few studies had multiple publications found through our searches. In these cases, the publication that provided the data used to calculate effect sizes was considered the main study and that is what is listed in tables, figures and the text. In cases where the effect size data were available in multiple publications, we treated the peer‐reviewed journal article as the main publication for the study (including in our coding of publication type). Secondary publications associated with the project that may have been used to help complete other items on our coding instrument are listed below the main publication in the list of eligible studies (via “see also” notes) in Section 5.1.1. There were no cases where unique crime/disorder outcomes for our main analyses were found across publications for the same study.

A common problem in conducting meta‐analyses in crime and justice is that investigators often do not prioritize the outcomes examined. This is common in studies in the social sciences in which authors view good practice as demanding that all relevant outcomes be reported. However, the lack of prioritization of outcomes in a study raises the question of how to derive an overall effect of treatment. For example, the reporting of one significant result may reflect a type of “creaming” in which the authors focus on one significant finding and ignore the less positive results of other outcomes. However, authors commonly view the presentation of multiple findings as a method for identifying the specific contexts in which a treatment is effective. When the number of such comparisons is small and therefore unlikely to affect the error rates for specific comparisons such an approach is often valid.

This is a particularly important issue for the current review. Given that POP calls for police to identify specific problems and develop tailor‐made solutions, it is important to include only outcomes likely to have been impacted by such focused responses. For example, in the Mazerolle et al. (2000) study, the authors noted that the Beat Health program “uses a variety of tactics to resolve drug and disorder issues” (p. 220). The authors present data on calls for service for disorder, drug crime, property crime, and violent crime. Because of their description of the intervention, we chose to include only drug and disorder calls as primary outcomes, and these were the outcomes we used for our mean effect size discussed below.

A primary outcome is defined in our review as one that was the direct focus of the POP intervention. The police needed to be specifically targeting the crime or call type in an outcome for us to identify an outcome as primary. We note that we erred on the side of being inclusive and only excluding reported crime/disorder outcomes in cases where the studies made it abundantly clear that only certain reported outcomes were the direct target of the tailored POP intervention. As such, for the vast majority of studies we include all reported crime/disorder outcomes.

We also note that it is important to examine variation in impacts across outcomes, and as such we analyze the studies using two approaches. The first is conservative in the sense that it combines all relevant outcomes reported into an overall average effect size statistic for each study. Second, to provide a range of effects, we also present separate models based on the largest and smallest effect for each study with multiple included outcomes. For studies with a sole outcome, or a clearly‐specified primary outcome, the same effect size is reported in all models. We also examined the impacts of POP across crime type.

In addition to providing a range, this approach is important as in some of the studies with more than one outcome reported, the largest outcome reflected what authors thought would be the largest program effect. This was true for the Jersey City Drug Market Analysis Experiment, which examined violent and property crimes, but assumed that the largest program effects, given the nature of the intervention, would be found in the case of calls for disorder (Weisburd & Green, 1995).

#### 4.3.4 Treatment of qualitative research

Qualitative research on crime and disorder outcomes was not included in this review. Our goal was to summarize the findings of experimental and quasi‐experimental evaluations of the quantitative impacts of POP on crime and disorder. Purely qualitative studies do not meet the inclusion criteria of the GPD and would not have come up in our searches. The authors encourage other researchers to examine whether there is a sufficient amount of qualitative research on POP to warrant a systematic review.

## 5 RESULTS

### 5.1 Selection of studies

#### 5.1.1 Results of the search

Search strategies in systematic reviews return a large number of results that must be screened for eligibility. Utilizing the GPD helped keep this number more manageable. Even though the GPD search strategy is much more comprehensive than those typically employed in searches by researchers conducting individual reviews, the studies included in the database have already been screened and confirmed to be policing evaluations that meet their methodological criterion (see above and the full details provided in Appendices B and C).

The initial steps of the review consisted of reviewing titles and abstracts to eliminate any duplicates and studies that were clearly not evaluations of POP. For any studies that could not be eliminated at this this step, we obtained the full‐text of the articles, reports, theses/dissertations or books for careful review to assess whether the interventions and evaluations met the eligibility criterion.

In total, the GPD searches and other strategies used in this review yielded a total of 2,464 results to review. Reviewing titles and abstracts eliminated 1,481 studies which were clearly not evaluations of POP. This left 983 studies which received full‐text review. Of these 39 met our eligibility criteria, and 24 provided the quantitative data needed to calculate effect sizes for our meta‐analyses. As the original Campbell systematic review of POP (Weisburd et al., 2008, 2010) included 10 experimental or quasi‐experimental studies in their main analyses we have a total of 34 studies included in our summary tables and meta‐analyses.

Of the 983 publications which received full‐text review, 746 studies were Goldstein or Tilley Award submissions (all available award submissions from 2006 to 2018 received full‐text review), 204 studies were GPD search results, and 33 studies were identified via forward citation searches.

**Figure 1:**
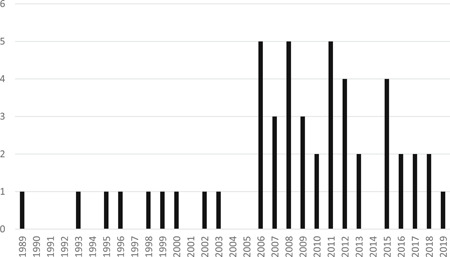
Number of eligible problem‐oriented policing studies by year (*N *= 49)

Figure 1 provides a visual summary of the number of eligible studies by year of publication. As the graph highlights, there was a clear increase in evaluations of POP in the years after the 2006 cutoff for our original review. This uptick was relatively evenly spread over the 12‐year period, with each year other than 2014 having between one and five eligible studies. While it is possible that some of the increase is due to the use of the GPD for searches for this update, the data suggest that there has simply been an increase in experimental and quasi‐experimental evaluations of POP since 2006. For example, the original review only included 2 Goldstein/Tilley award submissions, while our update found 11 new submissions which met our inclusion criteria and included the data needed for effect size calculations.

Below we list the 49 studies that met our inclusion criterion. We first list the 34 studies which provided the quantitative data needed to be included in our meta‐analyses. We refer to these as “included studies.” As noted above, the publication that provided data for the effect size(s) for each study is listed, and any supplemental publications that were used to complete other parts of our coding of studies are listed in sub‐bullets via “see also” notes. Below this, we list the 15 studies that are not included in our analyses due to not providing sufficient data to calculate effect sizes for either of our approaches. Both lists are in chronological order.

**Studies included in summary tables and meta‐analyses (*N *= 34):**

- Sherman, L., Buerger, M., & Gartin, P. (1989). *Repeat call address policing: The Minneapolis RECAP Experiment*. Washington, DC: Crime Control Institute.
- Stone, S. S. (1993). *Problem‐oriented policing approach to drug enforcement: Atlanta as a case study* (Ph.D. dissertation). Emory University.
- Weisburd, D., & Green, L. (1995). Policing drug hot spots: The Jersey City drug market analysis experiment. *Justice Quarterly*, *12*(4), 711–735.
- Stokes, R., Donahue, N., Caron, D., & Greene, J. R. (1996). *Safe travel to and from school: A problem‐oriented policing approach*. Washington, DC: U.S. Department of Justice.
- San Diego Police Department. (1998). *Coordinated agency network*. San Diego, CA: Herman Goldstein Award Submission.
- Braga, A. A., Weisburd, D. L., Waring, E. J., Mazerolle Green, L., Spelman, W., & Gajewski, F. (1999). Problem‐oriented policing in violent crime places: A randomized controlled experiment. *Criminology*, *37*(3), 541–580.
  ◦ See also Braga (1997).

- Mazerolle, L., Price, J. F., & Roehl, J. (2000). Civil remedies and drug control: A randomized field trial in Oakland, California. *Evaluation Review, 24*(2), 212–241.
- Knoxville Police Department. (2002). *The Knoxville public safety collaborative*. Knoxville, TN: Herman Goldstein Award Submission.
- Baker, T., & Wolfer, L. (2003). The crime triangle: Alcohol, drug use, and vandalism. *Police Practice and Research, 4*(1), 47–61.
- Nunn, S., Quinet, K., Rowe, K., & Christ, D. (2006). Interdiction day: Covert surveillance operations, drugs, and serious crime in an inner‐city neighborhood. *Police Quarterly*, *9*(1), 73–99.
- San Angelo Police Department. (2006). “*See! It's me!” identity theft prevention program*. San Angelo, TX: Herman Goldstein Award Submission.
- Tuffin, R., Morris, J., & Poole, A. (2006). An evaluation of the impact of the National Reassurance Policing Programme. London, UK: Home Office Research.
- Elliott, M. (2007). *An evaluation of specialized police response teams on motel crime* (Master's thesis). Reno, NV: University of Nevada.
  ◦ See also Reno Police Department (2006).

- Boston Police Department. (2008). *District D‐14: Breaking and entering solution plan*. Boston, MA: Herman Goldstein Award Submission.
- Braga, A. A., & Bond, B. J. (2008). Policing crime and disorder hot spots: A randomized controlled trial. *Criminology*, *46*(3), 577–607.
  ◦ See also Braga and Bond (2009).

- Lancashire Constabulary. (2008). “*Moppin” up dodge*. Lancashire, UK: Herman Goldstein Award Submission.
- Lexington Division of Police. (2009). *Community law enforcement action and response program*. Lexington, KY: Herman Goldstein Award Submission.
- Vancouver Police Department. (2009). *Reclaiming the “street of shame”: A problem oriented solution to Vancouver's entertainment district*. Vancouver, BC: Herman Goldstein Award Submission.
- Guseynov, N. R. (2010). *Policing serious crime: A longitudinal examination of geographically focused policing activities* (Master's thesis). University of Missouri‐Kansas City.
- London Borough of Enfield. (2011). *Safe as houses‐ domestic burglary project*. London, UK. Herman Goldstein Award Submission.
- Niagara County Sheriff's Office. (2011). *Operation panther pride*. Lockport, NY: Herman Goldstein Award Submission.
- Taylor, B., Koper, C. S., & Woods, D. J. (2011). A randomized controlled trial of different policing strategies at hot spots of violent crime. *Journal of Experimental Criminology*, *7*(2), 149–181.
- Houston Police Department. (2012). *Back from the brink: Reclaiming the Antoine corridor and the development of problem oriented policing within the Houston Police Department*. Houston, TX: Herman Goldstein Award Submission.
- Lancashire Constabulary. (2012). T*he custody experience: Reducing 1st time entrants into the criminal justice system*. Lancashire, UK: Herman Goldstein Award Submission.
- Bichler, G., Schmerler, K., & Enriquez, J. (2013). Curbing nuisance motels: An evaluation of police as place regulators. *Policing: An International Journal*, *36*(2), 437–462.
  ◦ See also Chula Vista Police Department (2009).

- Bond, B. J., & Hajjar, L. M. (2013). Measuring congruence between property crime problems and response strategies: Enhancing the problem‐solving process. *Police Quarterly*, *16*(3), 323–338.
- Groff, E. R., Ratcliffe, J. H., Haberman, C. P., Sorg, E. T., Joyce, N. M., & Taylor, R. B. (2015). Does what police do at hot spots matter? The Philadelphia policing tactics experiment. *Criminology*, *53*(1), 23–53.
  ◦ See also Ratcliffe, Groff, Sorg, and Haberman (2015).

- Hollywood Police Department. (2015). *West district burglary reduction initiative*. Hollywood, FL: Herman Goldstein Award Submission.
- Kochel, T. R., Burruss, G., & Weisburd, D. (2015). *St. Louis County Hot Spots in Residential Areas (SCHIRA) final report: Assessing the effects of hot spots policing strategies on police legitimacy, crime, and collective efficacy*. Carbondale, IL: Southern Illinois University
  ◦ See also Kochel and Weisburd (2017, 2019).

- Dario, L. M. (2016). Crime at convenience stores: *Assessing an in‐depth problem‐oriented policing initiative* (Ph.D. dissertation). Arizona State University.
  ◦ See also White and Katz (2013) and Glendale Police Department (2016).

- Durham Constabulary. (2017). *Reducing dwelling burglaries in areas which repeatedly suffer high rates in county Durham, UK*. County Durham, UK: Herman Goldstein Award Submission.
- Zidar, M. S., Shafer, J. G., & Eck, J. E. (2017). Reframing an obvious police problem: Discovery, analysis and response to a manufactured problem in a small city. *Policing: A Journal of Policy and Practice*, *12*(3), 316–331.
- Gill, C., Weisburd, D., Vitter, Z., Shader, C. G., Nelson‐Zagar, T., & Spain, L. (2018). Collaborative problem‐solving at youth crime hot posts: A pilot study. *Policing: An International Journal*, *41*(3), 325–338.
- Cooley, W., Bemiller, M., Jefferis, E., & Penix, R. (2019). Neighborhood by neighborhood: Community policing in a rust belt city. *Policing: An International Journal*, *42*(2), 226–239.^12^

**Eligible studies which lacked effect size data (*N* = 15):**

- Hampshire Constabulary. (2006). *Operation Mullion: Reducing anti‐social behaviour and crime in and around Mayfield School*. Portsmouth, Hampshire, UK: Herman Goldstein Award Submission.
- South Yorkshire Police. (2006). *Focusing on car crime: An initiative by South Yorkshire Police to tackle the problem of offenders stealing from Ford Focus cars*. Barnsley, South Yorkshire, UK: Tilley Award Submission.
- Charlotte‐Mecklenburg Police Department. (2007). *Operation safe storage*. Charlotte, NC: Herman Goldstein Award Submission.
- Regina Police Services. (2007). *Regina auto theft strategy*. Saskatchewan, Canada: Herman Goldstein Award Submission.
- Northhamptonshire Police. (2008). *Northampton Countywide Traveler Unit*. Northhamptonshire, UK: Tilley Award Submission.
- Sussex Police. (2008). *Operation athlete*. Sussex, UK: Tilley Award Submission.
- Anaheim Police Department. (2009). *Anaheim Police Department's GRIP on gangs: Gang reduction and intervention partnership: An early gang prevention problem solving strategy*. Anaheim, CA: Herman Goldstein Award Submission.
- Warwickshire Police. (2009). *Trolley safe: A design based problem solving response to reduce purse thefts from shoppers in supermarkets*. Warwickshire, UK: Herman Goldstein Award Submission.
- Dayton Police Department. (2011). *The urban high school disorder reduction project: Restoring safe schools and inspiring academic excellence*. Dayton, OH: Herman Goldstein Award Submission.
- State College Police Department. (2011). *Reducing crime and disorder in rental properties: An evaluation of the state college nuisance property ordinance*. State College, PA: Herman Goldstein Award Submission.
- Boston Police Department. (2012). Safe street teams problem‐oriented policing initiative. Boston, MA: Herman Goldstein Award Submission.
- Palm Beach County Sheriff's Office. (2012). *Smart Policing Initiative: Increasing police legitimacy and reducing victimization against immigrants in Lake Worth*. Lake Worth, FL: Herman Goldstein Award Submission.
- Wolfe, S. E., Rojek, J., Kaminski, R., & Nix, J. (2015). *City of Columbia (SC) Police Department Smart Policing Initiative: Final Report*. Retrieved from http://strategiesforpolicinginnovation.com/sites/default/files/2015_Wolfe%20et%20al_SPI_Final%20Report_Submission%20to%20CNA%20and%20BJA.pdf
- Portland Police Bureau. (2018). *Zombie houses: The Portland approach to vacant homes*. Portland, OR: Herman Goldstein Award Submission.
- Carson, J. V., & Wellman, A. P. (2018). Problem‐oriented policing in suburban low‐income housing: A quasi‐experiment. *Police Quarterly, 21* (2), 139–170.

Several studies that received full‐text review after the initial abstract screening were excluded after determining that they did not meet our inclusion criteria. These studies are noted in Appendix F.

### 5.2 Characteristics of selected studies

Table 1 provides an overview of the 34 studies that are included in our meta‐analyses. In terms of location, 28 studies (82.4%) were conducted in the United States, 5 (14.7%) in the United Kingdom and 1 (2.9%) in Canada. Studies were conducted in a total of 23 U.S. cities and 2 counties across 17 states. Lowell, MA, Jersey City, NJ and Philadelphia, PA all served as the jurisdiction for two studies. The U.K. studies included a total of eight jurisdictions, with Lancashire Constabulary serving as a study site in three evaluations. Vancouver was the setting of the Canadian study.

**Table 1:** Characteristics of included problem‐oriented policing interventions (*N *= 34)

Characteristic	*N*	%
Evaluation country		
United States	28	82.4
United Kingdom	5	14.7
Canada	1	2.9
Publication type		
Peer‐reviewed article	13	38.2
Award submission	13	38.2
Research report	4	11.8
Dissertation	2	5.9
Thesis	2	5.9
Evaluation type		
Randomized experiment	9	26.5
Quasi‐experiment	25	73.5
Problem unit		
Places/geographic areas	26	76.5
Place managers	4	11.8
Offenders	3	8.8
Victims	1	2.9
Displacement/diffusion^a^		
Tested quantitatively	8	26.7
Did not test quantitatively	22	73.3

^a^ Only the 30 studies that targeted places/place managers and could have thus tested for displacement/diffusion are included here.

The study documents we identified were predominantly peer‐reviewed journal articles (*N *= 13, 38.2%) and submissions to the Goldstein Award (*N *= 13, 38.2%) for excellence in POP (no Tilley Award submissions met our inclusion criteria). There were also 4 (11.8%) research reports, 2 (5.9%) doctoral dissertations, and 2 (5.9%) master's theses. A few scholars served as an author or coauthor on multiple included studies, including David Weisburd (four studies and a coauthor of this review), Lorraine Mazerolle (three studies), Anthony Braga (two studies), and Brenda Bond (two studies).

In terms of rigor of research design, our sample of studies includes 9 (26.5%) randomized experiments and 25 (73.5%) quasi‐experiments. This is an increase from four randomized experiments and six quasi‐experiments in the original review and suggests a trend toward more rigorous evaluation of POP since 2006.

Turning to the unit of analysis for the POP interventions in these studies, 26 (76.5%) programs targeted problem places, 4 (11.8%) were targeted at place managers, and 4 (11.8%) targeted individuals. Three of the individual‐focused programs targeted problem offenders, while one intervention was aimed at potential victims. Eight studies of place‐based POP approaches quantitatively assessed displacement and diffusion effects, and we conduct a meta‐analysis on these effects.

Table 2a provides a quick overview of the type of problems targeted and the type of responses delivered, while Table 2b provides a detailed summary of the studies based on type/depth of scanning for problems and problem analyses used, the responses delivered and the research design. The latter columns in the table also highlight implementation problems and research design limitations where applicable. These are discussed in detail in the following two sections.

**Table 2a:** Targeted problems and delivered responses in problem‐oriented policing experiments and quasi‐experiments

Study	Problem	Response
Baker and Wolfer (2003)	Alcohol, drug use, and vandalism at local park	Target hardening measures, proactive patrol, offender and victim‐focused community responses
Bichler et al. (2013); Chula Vista Police Department (2009)	Motel crime	Targeted motel management with increased training/supervision and accountability
Bond and Hajjar (2013)	Property crime hot spots	Directed patrols, increased drug and traffic enforcement, community meetings
Boston Police Department (2008)	Residential burglary	Increased available investigatory resources, target hardening measures, community education and targeted patrols
Braga et al. (1999); Braga (1997)	Violent crime hot spots	Order maintenance approaches, situational crime prevention and drug enforcement measures
Braga and Bond (2008)	Crime and disorder hot spots	Situational crime prevention, social service, and order maintenance strategies
Cooley et al. (2019)	Disorderly conditions in residential neighborhood	Area cleanup, intervention with landlords, partnership with the community and other CJS agencies, focused deterrence strategies
Durham Constabulary (2017)	Residential burglary	Target hardening and situational crime prevention measures, community meetings and education
Elliott (2007); Reno Police Department (2006)	Calls for service at low‐budget motels	Warrant sweeps, CPTED recommendations, and education for motel owners/managers, target hardening measures, partnership with community organizations
Gill et al. (2018)	Youth‐related crime and disorder	CPTED measures, partnership with community and social services, communication with other policing agencies, targeting problematic adult offenders
Groff et al. (2015); Ratcliffe et al. (2015)	Violent crime hot spots	Partnership with other agencies, targeting identified offenders, foot patrol
Guseynov (2010)	Crime and quality of life issues	Nuisance abatement and code enforcement, prosecution of landlords, area cleanup, targeting known drug dealers
Hollywood Police Department (2015)	Residential burglary	Target hardening and situational crime prevention measures, partnership with neighborhood watch programs
Houston Police Department (2012)	Violent, drug, and property crime	Code enforcement and nuisance abatement, warrant sweeps, CPTED surveys, crime prevention education for property managers
Knoxville Police Department (2002)	Rearrested probationers	Multi‐agency cooperation for case release and supervision plans, graduated sanctions
Kochel et al. (2015); Kochel and Weisburd (2017, 2019)	Hot spots of Part I and Part II crime	Target hardening education, nuisance abatement and code enforcement, area cleanup and community partnership
Lancashire Constabulary (2008)	Crime and calls for service in local neighborhood	Increased patrol and enforcement, target hardening and other situational prevention measures, community outreach and social service responses
Lancashire Constabulary (2012)	Youth reprimands	Identified most at‐risk youth, toured them through jail, and educated them about the future consequences of crime
Lexington Division of Police (2009)	Neighborhood crime and calls for service	Directed patrols and proactive enforcement of known offenders, code enforcement, situational crime prevention measures
London Borough of Enfield (2011)	Domestic burglary	Target hardening and other situational crime prevention measures, publicizing intervention and educating community
Mazerolle et al. (2000)	Drugs and disorder at problem locations	Targeted property managers to address underlying issues, partnered with local government to take civil action against uncooperative property managers
Niagara County Sheriff's Office (2011)	Crime and disorder in local town	Zero tolerance policing, curfew enforcement, nuisance abatement, partnership with the community and property owners
Nunn et al. (2006)	Drug and overall crime in local neighborhood	Drug interdiction consisting of intelligence gathering and large‐scale warrant sweep
San Angelo Police Department (2006)	Reported forgeries	Worked with retailers and financial institutions to encourage ID checks of paying customers, public advertisement and education of risks of ID theft
Sherman et al. (1989)	Calls for service at residential and commercial addresses	Helped landlords target problem tenants, worked with victims of domestic violence, commercial strategies varied widely by location
Stokes et al. (1996)	Violent victimization of students going to/from school	Creation of Safe Corridor with increased police presence
Stone (1993)	Drugs in public housing projects	Situational crime prevention measures and area cleanup, visual and safety improvements
Taylor et al. (2011)	Violent crime hot spots	Situational crime prevention measures, code enforcement, and nuisance abatement, working with the community and other stakeholders
Thomas (1998)	Rearrests of juvenile probationers	Coordinated Agency Network increasing community supervision and resources between police and probation
Tuffin et al. (2006)	Antisocial behavior and “juvenile nuisance”	Increased police presence, varied responses involving community stakeholders
Vancouver Police Department (2009)	Street disorder calls for service in local entertainment district	Environmental redesign and street closures at hot times, partnership with local organizations, targeting known gang offenders
Weisburd and Green (1995)	Hot spots of drugs and disorder	Coordinated crackdowns, maintenance levels of surveillance after crackdowns
White and Katz (2013); Dario (2016); Glendale Police Department (2016)	Calls for service at Circle K convenience stores	Attempted cooperation with store leadership, CPTED surveys and recommendations, publicization measures, suppression and enforcement efforts
Zidar et al. (2017)	Shoplifting at local Walmart stores	Implementation of new reporting system, officers no longer responded to thefts of less than $500

**Table 2b:** SARA model characteristics of problem‐oriented policing experiments and quasi‐experiments

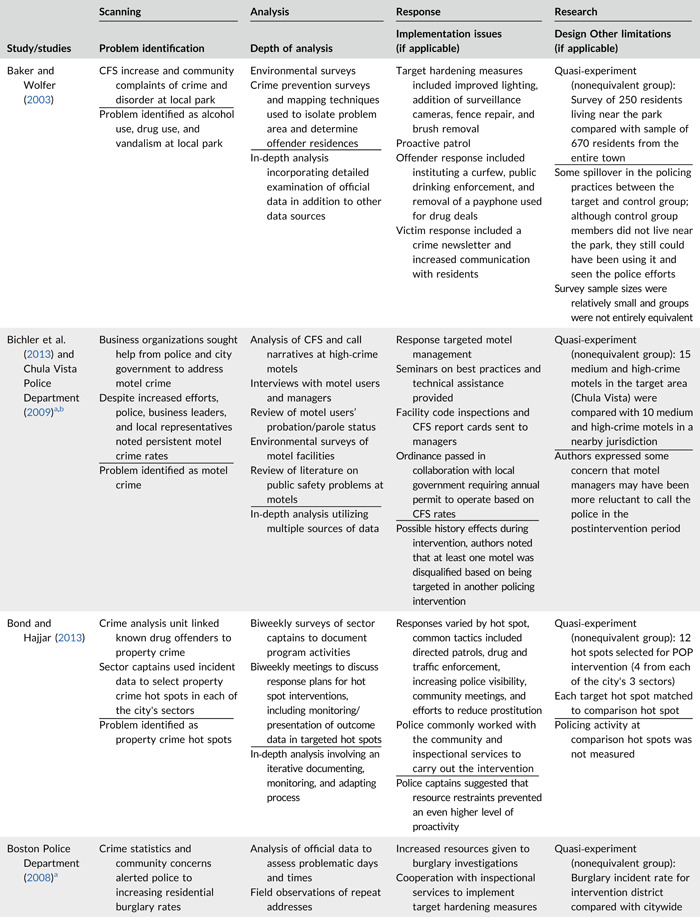
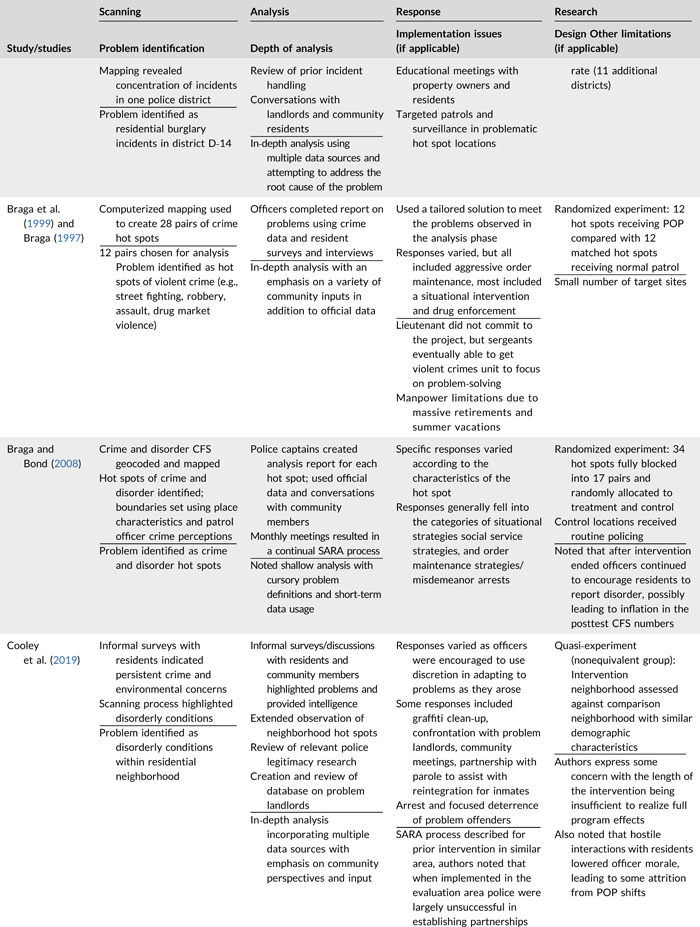
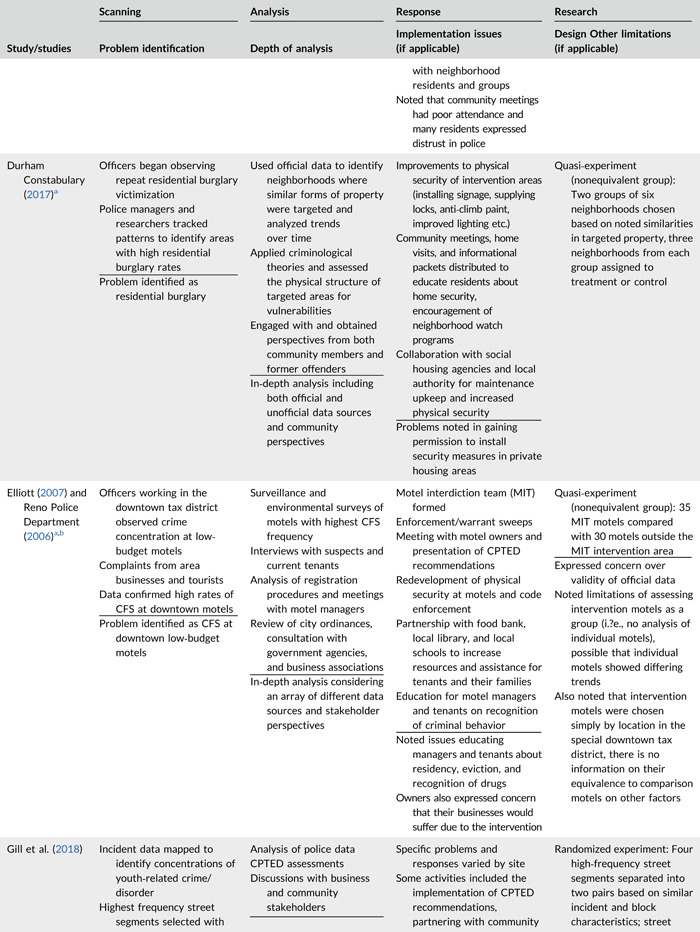
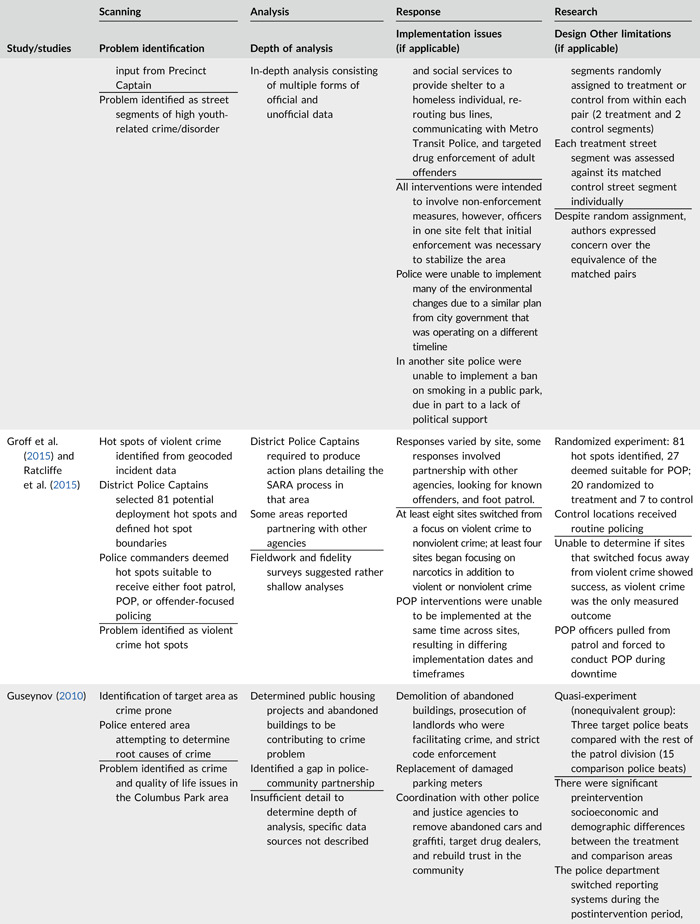
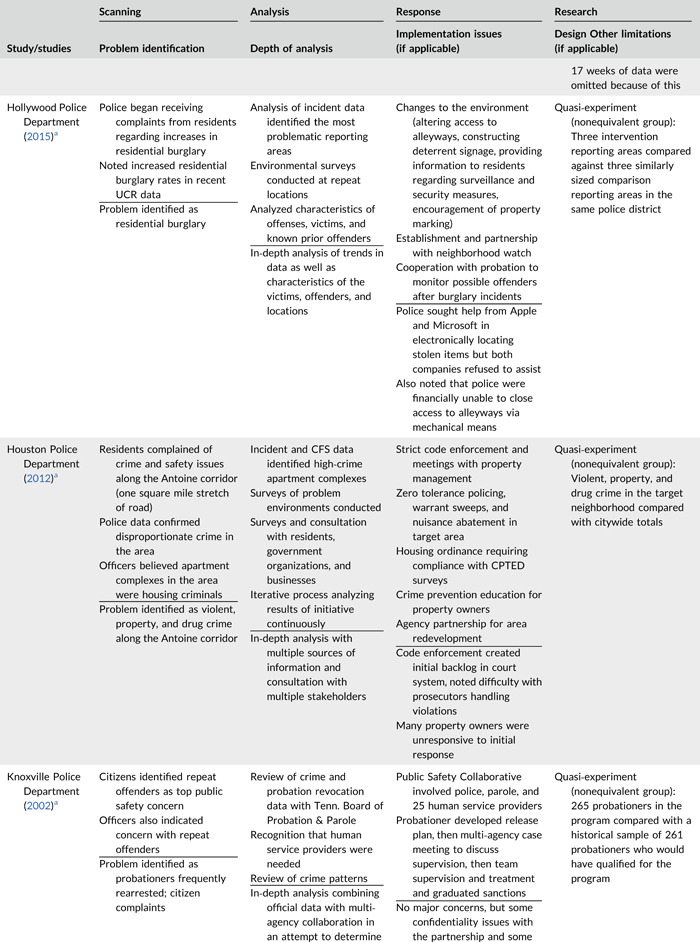
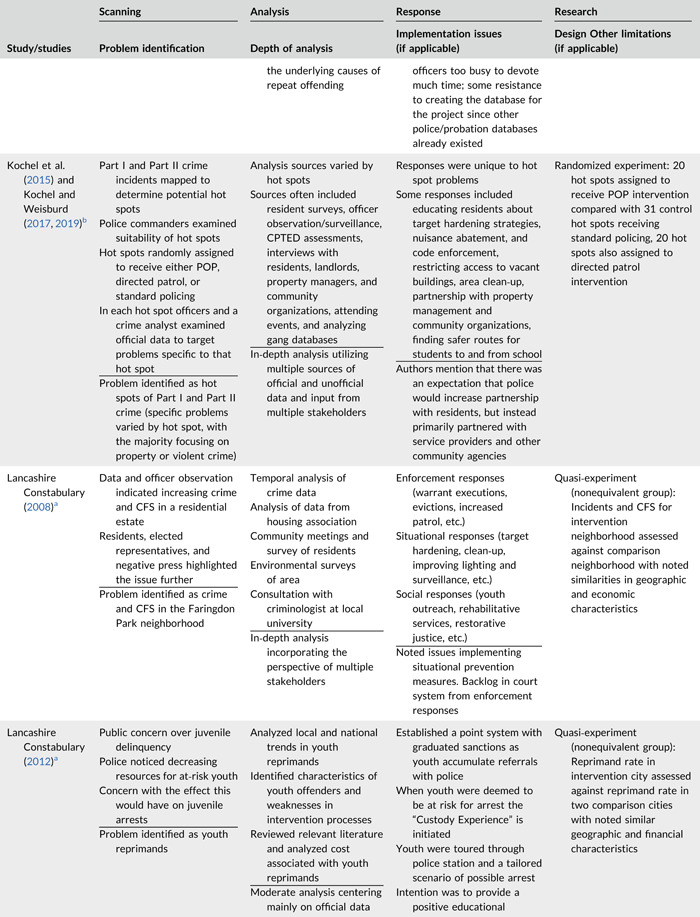
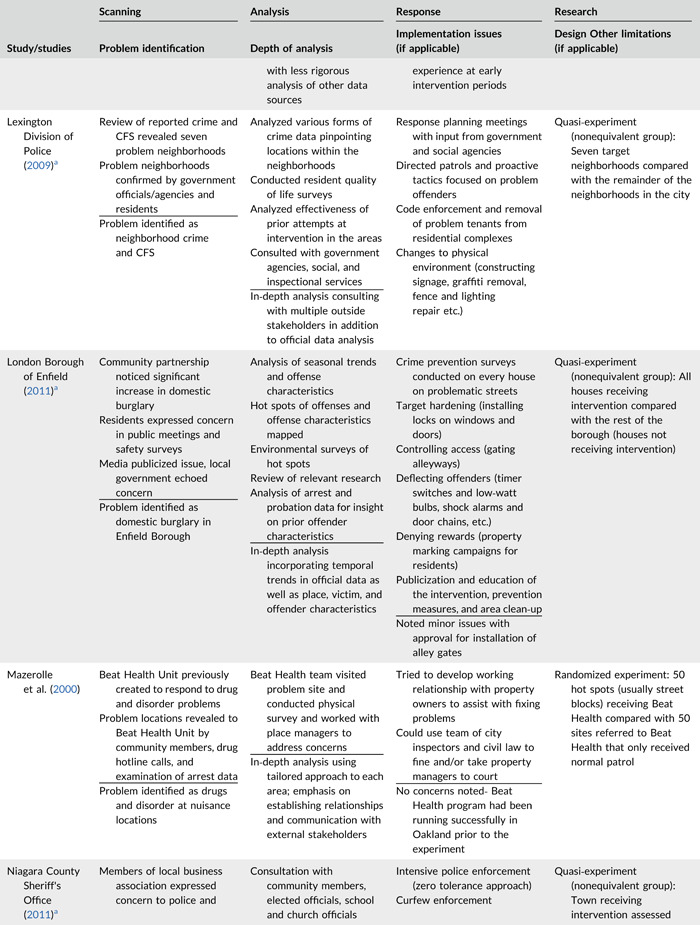
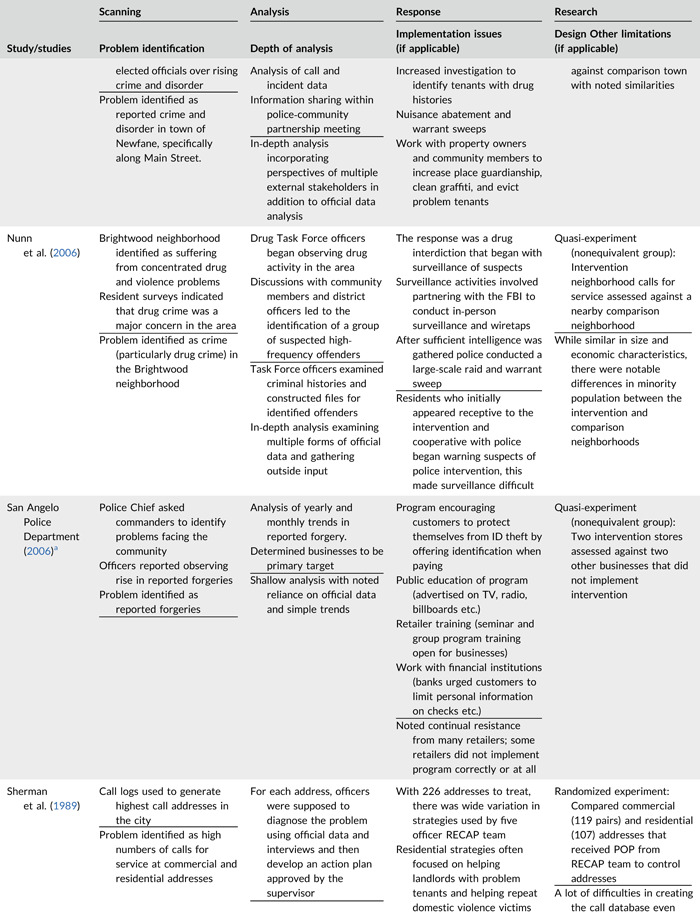
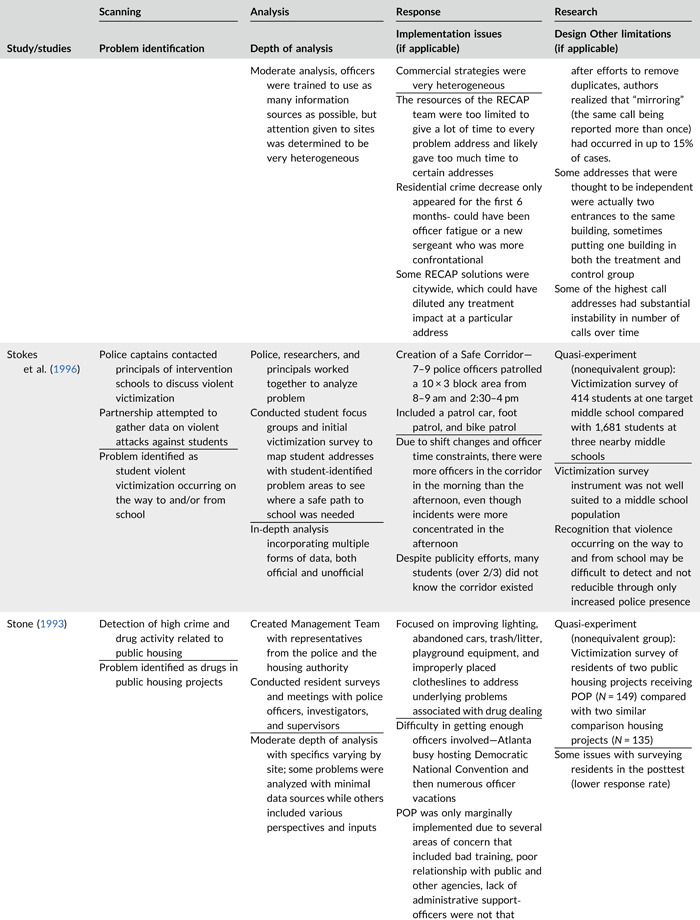
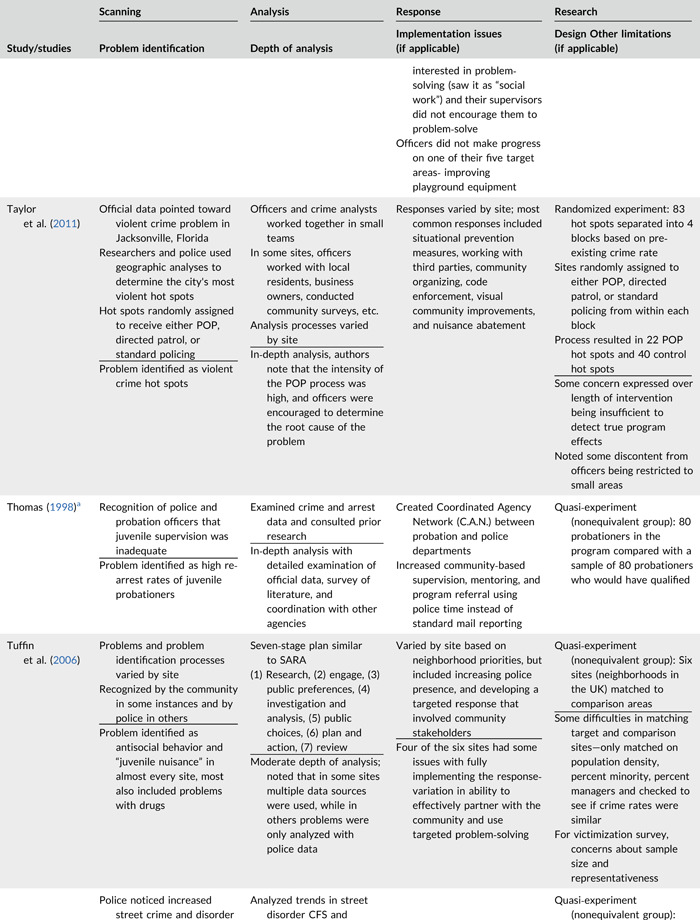
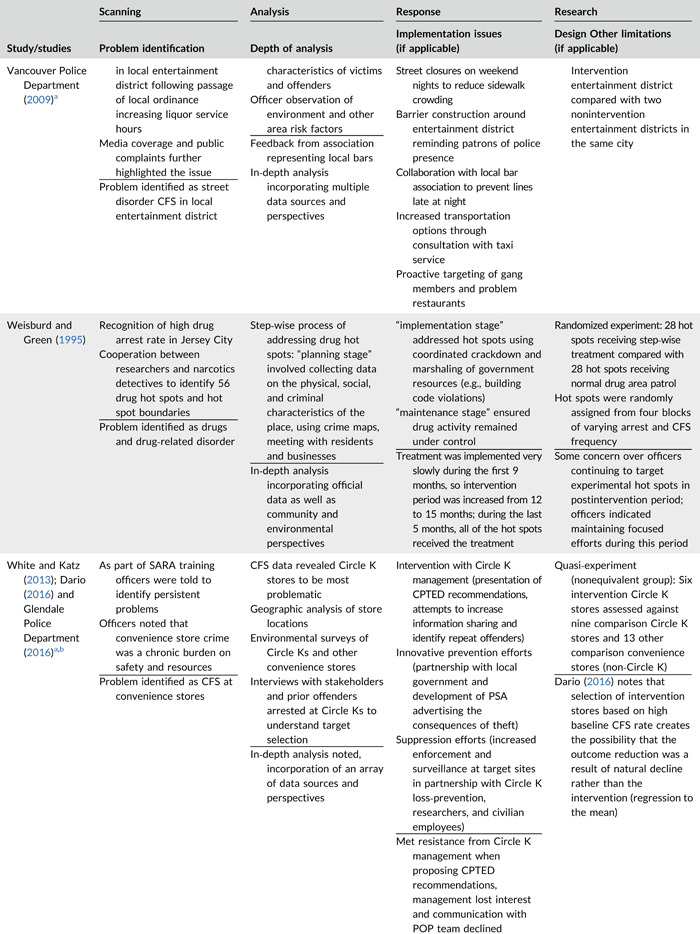
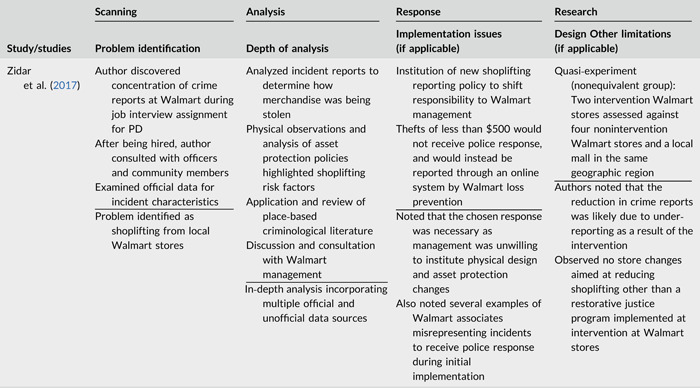

*Note:* The descriptions provided are summaries and are not intended to cover every aspect of the intervention.

^a^ Goldstein Award Submission.

^b^ Report used for intervention description but not included in meta‐analysis.

Finally, 15 of 34 (44.1%) studies reported significant reductions in at least one crime or disorder outcome, while another 17 (50%) studies reported raw differences favoring the treatment group for at least one crime or disorder outcome. Table 3 provides a summary of study conclusions about impacts on crime and disorder, as well as displacement and diffusion of crime control benefits where applicable.

**Table 3:** Impacts of problem‐oriented policing on crime and disorder outcomes and displacement/diffusion

Study	Crime/disorder outcomes	Displacement/diffusion
Baker and Wolfer (2003)	Victimization survey shows pre to post drops in target group noting vandalism in past 6 months and noticing drinking/disorderly conduct in past 6 months In presurvey, target group had significantly higher victimization rates that were not significantly different from the control group by postsurvey	Not tested, but mention of benign displacement as offenders moved from park to more open spaces downtown
Bichler et al. (2013)	Calls for service dropped significantly more at intervention hotels than control hotels.	No evidence of displacement from Chula Vista hotels to similar hotels in neighboring jurisdictions
Bond and Hajjar (2013)	Treatment hot spots showed greater declines in aggregate property crime than comparison hot spots	Not tested
	Reductions ranged from 16% to 19% per sector	
Boston Police Department (2008)	Intervention district saw a 40% drop in residential burglary, while rest of city saw an 8.8% increase	Not tested, but mention that an examination of crime in neighboring jurisdictions provides no evidence of displacement
Braga et al. (1999); Braga (1997)	Total calls and incidents significantly lower at treatment hot spots than control hot spots Significantly fewer street fight, property, and narcotics calls in treatment hot spots, no significant difference in robbery and disorder calls Significantly fewer robbery and property incidents in treatment hot spots, no significant impact on nondomestic assault and disorder incidents and narcotics arrests	Significant evidence of displacement into catchment areas for property crime, but no evidence for other crime types and more evidence of diffusion of crime control benefits for disorder calls, assault incidents, and total calls
Braga and Bond (2008)	Total calls dropped 19.8% in treatment relative to control hot spots Assault, robbery, burglary, and disorder calls are significantly less in treatment hot spots, no significant difference in larceny calls	No significant increase in crime in treatment hot spot catchment areas, although data do show increases in all crime categories, suggesting potential for some displacement
Cooley et al. (2019)	Violent crimes and quality of life crimes decreased slightly in the intervention area, while increasing slightly in the comparison area; differences were not statistically significant	Not tested
Durham Constabulary (2017)	Dwelling burglary counts dropped significantly in target areas relative to control sites Drops both in 3 sites that had preintervention cash/jewelry burglaries and three sites that did not have cash/jewelry burglary issues	Mentioned no evidence of displacement, but no data provided
Elliott (2007); Reno Police Department (2006)	Total calls for service decreased 7% at targeted hotels and increased 15% at comparison hotels Drops in disorder and person calls in targeted versus comparison hotels No impact on property calls	Not tested
Gill et al. (2018)	In Westlake Park, treatment site had slightly more calls and incidents relative to control site, not statistically significant On Retail St, treatment hot spot had significantly fewer calls and fewer incidents relative to control site	Not tested, because treatment hot spots were so close together
Groff et al. (2015)	No significant impact of POP on violent crime incidents or violent crime felonies in treated hot spots relative to controls	Not tested, because no impact of POP on crime
Guseynov (2010)	Significant pre to post drop in treatment beats in Part I crimes, but no significant difference in pre to post changes in treatment and comparison areas, suggesting limited crime prevention impact of program	Not tested
Hollywood Police Department (2015)	Burglaries decline 13.7% pre to post in three target reporting areas and increase 33.8% in three comparison reporting areas	Small amount of displacement noted, but no data provided
Houston Police Department (2012)	Reduction in Part I incidents pre to post in treatment area that is greater than citywide reduction in crime	Short‐term displacement mentioned, but no data provided
Knoxville Police Department (2002)	38% reduction in recidivism in treated parolee group compared with historical comparison group (29% success rate in treated group versus 11% in historical group)	Not tested
Kochel et al. (2015)	Calls for service drop significantly in problem‐solving sites pre‐ to during‐intervention (92.4 calls per month to 85.1 calls per month) with no significant decline in control sites	Not tested
Lancashire Constabulary (2008)	All crime in response area dropped 46.9% pre to post, while increasing 3.2% in comparison area All calls for service dropped 45.9% in response area and dropped only 4.1% in comparison area	No evidence of spatial displacement, displacement area shows small drops in crime and calls pre‐ to postintervention
Lancashire Constabulary (2012)	33% drop in youth reprimands in target area pre to post‐intervention versus 11% and 14% increases in youth reprimands in two control sites	Not tested
Lexington Division of Police (2009)	7.5% decrease in reported crimes in targeted neighborhoods comparing 2 years preintervention to 2 years post, compared with a 0.8% decrease in crime in the rest of the city	Not tested
London Borough of Enfield (2011)	59.3% reduction pre to post in burglaries in houses that received alley gate intervention versus 9.7% reduction in burglary in nearby houses that did not receive the intervention	Decrease in burglaries in larger neighborhood suggests potential of diffusion of crime control benefits; also some concerns of geographic displacement to other parts of Enfield where burglary hot spots emerged
Mazerolle et al. (2000)	Significant decrease in drug calls for service pre to post in experimental versus control hot spots (decrease in drug calls in experimental sites and increase in control sites) No significant change in disorder calls for service in experimental versus control hot spots (both see small increase in disorder calls per site)	Decline in drug calls in residential treatment catchment areas (vs. increase in control residential catchment areas) but increase in commercial site drug calls (in both groups). Suggests potential for diffusion of benefits for residential sites, and some displacement for commercial sites
Niagara County Sheriff's Office (2011)	60% decrease in crime incidents in Town of Newfane following intervention; similar Town of Porter (comparison town) shows 7% incident decrease in same time period	Not tested
Nunn et al. (2006)	Serious crime calls dropped 10.2% in postperiod in target area versus 9.2% in comparison area For particular call types, burglary, guns, personal violence, robbery, and theft all decreased in the treatment area, with drug calls increasing (which could, in part, reflect increased reporting after intervention) In the control area, burglary, drugs, guns, personal violence, robbery, and theft calls all decreased Percentage decreases were greatest in treatment area for robbery (−31.8% vs. −5.4% in control) and guns (−34.8% in treatment vs. −25.4% in control)	Not tested, because dealers arrested in intervention were sent to prison
San Angelo Police Department (2006)	Forgery cases declined 74.3% pre to post in two targeted stores and declined just 15.9% in nonimplementing stores	Not tested
Sherman et al. (1989)	Small decrease in calls for service in treatment residential addresses compared with control (6.01% treatment group decrease compared with 0.10% increase in control group), especially for the first 6 months of the experiment No difference in commercial addresses	Not tested
Stokes et al. (1996)	Victimization rate in test school increased in second victimization survey from 19.4% to 20.2% There was a statistically significant decrease in victimization at the control schools (21.1% down to 15.2%)	Not tested
Stone (1993)	Rate of being asked to buy or sell drugs increased significantly in intervention and control areas, but a greater increase in intervention area (up 68.29% vs. 30.88% in control area) Narcotics arrests and violent crime decreased in intervention area compared with control area, but total crime and property crime were higher in intervention area	Not tested
Taylor et al. (2011)	For calls for service, pre to post POP hot spots show declines in nondomestic violence, property, and any violence; declines are greater than control group declines only for property crime For incidents, pre to post POP hot spots show declines in nondomestic violence, property, and any violence; decline are greater than control group declines for both categories of violence	No significant differences in crime incidents in the area surrounding POP hot spots
		POP catchment areas had a 29% increase in any violence calls for service and a 31% increase in street violence calls for service in postperiod This could reflect more reporting as a result of nearby residents being aware of intervention, or could reflect displacement
Thomas (1998)	27% of treatment group participants successfully completed probation versus 20% of control group participants Participants had ¼ the rate of recidivism (6% vs. 22% for control group)	Not tested
Tuffin et al. (2006)	Two of the six sites had significantly larger reductions in total recorded crime than the controls Three of the sites had crime declines similar to the controls One site had a crime increase and the control had a significant crime decrease	Not tested
Vancouver Police Department (2009)	Calls for service declined 20.3% in targeted entertainment neighborhood pre to post while increasing 31.2% in two nontargeted comparison entertainment districts	Minimal evidence of displacement within the larger policing zone, some possibility that increase in one entertainment district was offenders displacing to this district
Weisburd and Green (1995)	Experimental group has significantly smaller increases in disorder calls (especially public morals, assistance, suspicious persons) compared with control group No impact on violent or property calls Difficult to determine impact on drug calls as the experiment itself likely changed reporting behavior	Calls for service not more likely to be displaced to experimental catchment areas; instead there appeared to be a diffusion of crime control benefits to two‐block areas surrounding experimental hot spots New hot spots two times more likely to appear in control group catchment areas
White and Katz (2013); Dario (2016)	Calls for service decrease 42% pre to post in targeted stores versus decreasing 31% in similar nontargeted stores	No evidence of displacement to nearby areas for any of the six targeted stores
Zidar et al. (2017)	45.2% pre‐ to postreduction in larcenies less than $500 at targeted Walmart, while comparison Walmarts generally had no significant change (two comparison sites show larceny decreases and three show larceny increases)	Not tested

### 5.3 Study implementation

While there was a relative lack of substantial complications reported, 67.6% (*n* = 23) of the 34 included studies did identify some degree of difficulty during implementation. Based on our coding criteria, the severity of these issues was classified as either minor (*n* = 15), more substantial (*n* = 7), or major (*n* = 1). There also appeared to be thematic consistencies across the studies in the types of issues reported. While such issues are in no way limited to POP interventions, this is perhaps indication that POP interventions are at greater risk of certain complications. Brief summaries of each study's implementation issues are also presented in Table 2b.

Given that POP is an iterative process, such that problems and responses are often continually changing, implementation issues may arise from problem instability. In the Philadelphia Policing Tactics experiment, Groff et al. (2015) note that nearly half of the POP intervention hot spots began focusing on nonviolent crime problems after determining that violent crime was no longer the primary concern in the area. Specifically, eight POP sites were noted to have targeted nonviolent or quality of life offenses, and at least four POP sites were noted to have focused on drug crime in addition to a violent or nonviolent crime problem. This is an issue from an evaluation standpoint as violent crime incidents were the only measured outcomes; thus it is possible that the interventions were effective in ameliorating the problems that they targeted, but this was unclear absent measurement of those outcomes. The RECAP experiment (Sherman et al., 1989) also suffered from issues related to problem instability. Specifically, call trends for many high‐crime addresses were remarkably heterogeneous from year to year, subsequently reducing the experiment's statistical power. The RECAP experiment and the Philadelphia Policing Tactics experiment both suffered from additional resource constraints as well. In the Philadelphia Policing Tactics experiment, POP officers were not dedicated to the intervention full‐time, but were instead drawn from patrol and expected to conduct POP activities during their free time. Similarly, in the RECAP experiment, there were likely too many addresses assigned to the experimental unit, thus spreading the unit's resources too thin and, perhaps, contributing to the lack of effectiveness in the second half of the intervention year.

The reality of resource constraints and other internal barriers to proper program implementation was not uncommon across these studies. Stokes (1996) reported that the safe travel corridor was poorly staffed during the afternoon hours, despite violence being more prevalent during this time. It was subsequently determined that this incongruence was due to officer shift changes and high numbers of outside calls during the afternoon hours. These factors created a gap in coverage and limited police resources toward the intervention (though the authors note that officer presence was adequate due to Temple University Police presence). It was also revealed that very few students were aware of the safety corridor, though it is not clear whether this was resource related, as school administration reported that the corridor was advertised over school announcements and letters that were distributed to students and parents. Stone (1993) also reported organizational and resource‐related constraints during the Atlanta public housing POP project. There was a relative lack of interest regarding the intervention within the department, little administrative support, and police training was minimal. These issues were compounded by the fact that the city of Atlanta had hosted the Democratic National Convention prior to the intervention, which forced officers to delay vacation during this time. Thus, when the POP project started, many officers opted to take time off and the project was chronically understaffed. In their evaluation of the Lowell (MA) Smart Policing Initiative (SPI, now referred to as Strategies for Police Innovation), Bond and Hajjar (2013) also noted that a common complaint from police captains was a lack of resources. It was suggested that such constraints prevented an increased level of proactivity during the intervention, though these issues appeared to be minor as the intervention was still considered to be effective. Minor issues with internal resistance were also noted by the Knoxville Police Department (2002) during their Public Safety Collaborative.

Police subversion concerns created barriers for both the Jersey City violent places POP study (Braga, 1997; Braga et al., 1999) and the Drug Market Analysis Program (Weisburd & Green, 1995). Partly as a result of officer resistance, the Drug Market Analysis Program achieved limited implementation in the first 9 months, with only nine hot spots receiving all program elements. This forced Weisburd and Green (1995) to increase the length of the intervention and develop a more detailed implementation schedule, and ultimately the program was fully implemented for the last 5 months of the intervention period. Braga (1997) noted similar resistance among officers in the violent places POP project, as well as a disconnect between middle management and department headquarters that threatened the integrity of the program and slowed progress during the first 8 months. Ultimately the intervention unit was placed under new leadership and protocols were established to document instances of subversion. Braga also noted significant organizational changes, such as an influx of retirement and scheduled vacations which strained resources and reduced the sample size of the experiment.

Another frequent implementation barrier was resistance from stakeholders that were intended to be involved in the intervention. In the Glendale SPI study, White and Katz (2013) indicated that in phase I of the response, the SPI team was largely unsuccessful in working with Circle K management to change the physical structure and operating policies of the stores. They note that, at this stage, communication between Circe K representatives and the SPI team suffered, and the intervention was forced in a different direction. Partly as a result of this, Dario (2016) expressed concern over treatment dosage, noting that treatment quality likely varied by store location due, in part, to differing levels of responsiveness. In their attempted intervention with Walmart management, Zidar et al. (2017) also reported resistance toward environmental and policy‐oriented intervention measures. This resistance dictated the future direction of the program, as the initial plan to partner with Walmart seemed futile. In response, Zidar et al. forced responsibility on Walmart leadership by forcing them to handle petit shoplifting incidents without police assistance; however, even after doing so they noted instances of Walmart loss‐prevention misrepresenting case facts to illicit police response.

The San Angelo Police Department (2006) cited heavy opposition from business owners to the implementation of an identification checking program, largely over concern that the program would inconvenience customers. This resistance forced the department to shift responsibility for the intervention toward the customer, though despite this, few businesses ever became willing to implement the program. The Motel Interdiction Team (MIT) program in Reno experienced similar complications (Elliott, 2007; Reno Police Department, 2006). Motel owners were concerned about the economic ramifications that would result from the eviction of criminal tenants. The Reno Police Department (2006) noted that it became difficult to educate these owners about recognition of criminal behavior and eviction processes, ultimately slowing the intervention's progress. In response to uncooperative property owners, the Houston Police Department (2012) increased code enforcement in their targeted intervention of the Antoine corridor. However, this response temporarily led to a backlog in the court system, and prosecutors began dropping charges (though this issue was subsequently resolved by use of specialized prosecutors). Lancashire Constabulary (2008) noted a similar delay in court processing based on their enforcement responses.

Several studies reported resistance from other outside stakeholders such as neighborhood residents, community organizations, and local government. Cooley et al. (2019) described an attempted POP replication in Canton, OH. However, while the initial intervention was successful in establishing partnerships with community residents and neighborhood groups, the attempted replication was not. Cooley and colleagues noted that there was a lack of community organizations available to partner with and that local residents were distrusting of and unwilling to cooperate with police. Local community meetings were unsuccessful at bridging the gap between law enforcement and neighborhood residents, officer morale was low, and ultimately the intervention showed limited effectiveness. Issues were encountered forming resident partnerships in the St. Louis County Hot Spots in Residential Areas experiment as well (Kochel & Weisburd, 2017). Specifically, program evaluation revealed that resident partnerships were less frequently established than had been originally intended. While residents appeared to be cooperative in the early stages of the Brightwood Interdiction project, the same residents were subsequently caught tipping off offenders to police surveillance during the intervention (Nunn et al., 2006). This slowed the evidence gathering process, but police were ultimately able to generate enough evidence to execute the planned warrant sweep. Lastly, as a result of varying levels of difficulty partnering with the community, Tuffin et al. (2006) reported that only two of six intended sites receiving full implementation, though the sites that did achieve full implementation showed strong results.

Gill et al. (2018) noted several aspects of the collaborative problem‐solving intervention in Seattle (WA) that were halted by local government resistance. In one intervention area, officers sought to implement a smoking ban, but were unable to do so largely due to a lack of political support. In another target area, officers were unable to implement environmental changes due to a city redevelopment plan that was operating on a different timeline. Gill et al. also noted that, despite the project's intention to be fully non‐enforcement, officers in one of the intervention areas felt that enforcement measures were necessary to stabilize the area.

Studies that targeted residential burglary incidents reported minor issues with environmental changes to the target area. London Borough of Enfield (2011) referenced issues with the installation of alley gates. Installation required 100% approval from area residents; however, several properties were rentals with out of town owners. The police were subsequently able to adjust the approval rate from 100% to 98% to circumvent this issue. Durham Constabulary (2017) noted similar issues installing security measures in private housing areas; however, they were eventually able to gain permission to do so. Lancashire Constabulary (2008) also documented issues implementing situational responses that altered the physical environment, and the Hollywood Police Department (2015) determined that closing access to alleyways was not financially feasible (however, they promoted the use of see‐through fencing instead).

Finally, there were some problems unique to certain studies. Sherman et al. (1989) encountered issues with hot spot selection, discovering that up to 15% of calls were “mirrors,” or duplicates created as a result of multiple 911 calls for the same incident. Additionally, several addresses that were originally believed to be independent were subsequently determined to correspond to the same building. This led to the inclusion of some buildings in both treatment and control groups, and required a series of pairwise deletions to modify the initial assignments. Bichler et al. (2013) expressed some concern over the possibility of unknown history effects, noting that at least one motel location was disqualified from their study after being the target of another policing intervention. Ultimately, however, there was no reported evidence to suggest that similar issues occurred at other intervention motels.

It is also worth noting that these studies may vary in their level of reporting validity. All interventions are likely to encounter obstacles; however, it is not necessarily the case that all such obstacles are accurately documented. At times there may be incentive to represent an intervention in the best possible light, perhaps at the expense of complete transparency. While this is certainly true of any form of research, it bears reminding that our determinations are limited to what was reported in study publications.

### 5.4 Risk of bias in included studies

Five main measures from our coding instrument (see Appendix E) were used to assess potential sources of bias in our included studies. These items included: (a) Were any sources of nonequivalence or bias reported or implied in the application of the intervention or its analysis (i.e., threats to internal validity)? (b) If yes, what sources of nonequivalence or bias were identified? (c) Did the researcher(s) express any concerns over the quality of the data? (d) If yes, explain. (e) If a quasi‐experiment, how was matching of groups achieved? The studies that reported issues along these dimensions and/or compared treatment groups to the rest of a jurisdiction or population are presented in Table 4. The remainder of our included studies reported no such issues and/or employed higher quality matching procedures.

**Table 4:** Assessment of risk of bias in eligible problem‐oriented policing studies

Study/studies	Nonequivalence^a^	Sources of nonequivalence^b^	Data quality concerns^c^	Sources of data quality concerns^d^	Matching process^e^
Baker and Wolfer (2003)	No	N/A	No	N/A	Comparison with the rest of a jurisdiction or population that did not receive the treatment
Boston Police Department (2008)	No	N/A	No	N/A	Comparison with the rest of a jurisdiction or population that did not receive the treatment
Elliott (2007)	No	N/A	Yes	Unclear, only noted concerns over data validity	Comparison to the rest of a jurisdiction or population that did not receive the treatment
Gill et al. (2018)	Yes	Concern over the equivalence of matched pairs	No	N/A	N/A
Guseynov (2010)	Yes	Pretest analyses indicated nonequivalence between treatment and control groups	Yes	New reporting systems resulted in 17 weeks of omitted data	Comparison with the rest of a jurisdiction or population that did not receive the treatment
Houston Police Department (2012)	No	N/A	No	N/A	Comparison with the rest of a jurisdiction or population that did not receive the treatment
Lexington Division of Police (2009)	No	N/A	No	N/A	Comparison with the rest of a jurisdiction or population that did not receive the treatment
London Borough of Enfield (2011)	No	N/A	No	N/A	Comparison with the rest of a jurisdiction or population that did not receive the treatment
Sherman et al. (1989)	Yes	Pretest analyses indicated nonequivalence between treatment and control groups	No	N/A	N/A
White and Katz (2013); Dario (2016)	Yes	Selection of treatment area based on high baseline crime rate	No	N/A	Comparison with the rest of a jurisdiction or population that did not receive the treatment
Zidar et al. (2017)	Yes	Measurement confounds (measure changes over time)	No	N/A	Comparison with the rest of a jurisdiction or population that did not receive the treatment

^a^ Were any sources of nonequivalence or bias reported or implied in the application of the intervention or its analysis (i.e., threats to internal validity)?

^b^ If yes, what sources of nonequivalence or bias were identified? (check all that apply and explain).

^c^ Did the researcher(s) express any concerns over the quality of the data?

^d^ If yes (authors expressed concern over quality of data), explain.

^e^ If a quasi‐experiment, how was matching of groups achieved?

Overall, only 14.7% (*n* = 5) of studies reported internal validity concerns and 5.9% (*n* = 2) of studies reported concerns over data quality. However, the validity of the matching techniques used in our sample of studies does need to be considered, as 40.0% (10 of 25) of the identified quasi‐experiments used the rest of a jurisdiction or population not receiving treatment as the comparison unit. Additionally, the remaining quasi‐experimental evaluations exclusively matched comparison units based on descriptive and demographic characteristics, or simple statistical tests of such characteristics. None of the included quasi‐experimental evaluations reported propensity or regression‐based matching techniques.

Of the randomized experiments (*n* = 9) included in this review, very few reported concerns over randomization procedures or other issues related to internal validity and bias. All experimental studies included some form of blocking or pair‐matching technique in addition to randomization. However, specific concerns were noted in a few of these experiments. In the RECAP experiment (Sherman et al., 1989, p. 17), there was possible contamination (or “spillover” effects) as treatment and control addresses were, at times, under shared ownership. Moreover, the instability in the call frequencies of particular addresses created additional variability between treatment and control groups. However, ultimately the groups were reported to be roughly equivalent. Despite the use of matched‐pair techniques in the Collaborative Problem‐Solving at Youth Crime Hot Spots study, the treatment and control locations were unable to be optimally matched, and Gill et al. (2018) noted some concern over the equivalence of these matched pairs.

The risks of bias in the quasi‐experimental evaluations are undoubtedly greater. However, of the 15 studies that matched treatment to comparison locations based on simple analysis of descriptive, social, or demographic characteristics, no major concerns were reported. It should be noted, however, that few of these studies (*n* = 4) were identified as providing a visual comparison of descriptive statistics between treatment and comparison areas. The remaining studies (*n* = 11) described the rationale and/or process for selection of comparison units, but did not provide further evidence that equivalence was attained.

The most notable concerns among our included studies were related to the use of particularly nonequivalent comparison groups. There were six studies that compared small geographic treatment areas (or collections of small geographic treatment areas) to city, district, or other population‐wide trends (Boston Police Department, 2008; Guseynov, 2010; Houston Police Department, 2012; Lexington Division of Police, 2009; London Borough of Enfield, 2011; White & Katz, 2013). Of note, four of these studies are Goldstein Award submissions, and while they do not report substantial concern over the comparability of the units, there are clear threats to internal validity caused by the use of such comparisons. There were another five studies that compared treatment units to the remainder of a population not receiving treatment, but where the size discrepancy between the groups was not as large (Baker & Wolfer, 2003; Elliott, 2007; San Angelo Police Department, 2006; Zidar et al., 2017).

In addition to the inherent threat to internal validity, several of the weaker quasi‐experimental studies reported unique issues. Comparison of descriptive statistics for the treatment and control areas in the CSTAR intervention (Guseynov, 2010) indicated statistically significant differences on measures of race, unemployment, poverty, single parent households, and population mobility. Both Guseynov (2010) and Elliott (2007) also reported data‐related concerns. Guseynov specifically notes that the Kansas City Police Department switched reporting systems during the beginning of the postintervention period. This switch resulted in the omission of 17 weeks of data, possibly leading to bias in the analysis. Zidar et al. (2017) suggested that the significant decline in reported shoplifting incidents indicated by their evaluation may have been the result of under‐reporting rather than true changes in the outcome. The intervention involved the implementation of a new reporting system for target Walmarts only. Thus, they imply that it is likely the observed outcome was the result of this measurement change rather than changes in the incident rate. In the Glendale SPI (Dario, 2016; White & Katz, 2013), the intervention locations were selected based on high pre‐existing crime rates. Dario (2016, p. 99) noted the inherent potential of regression to the mean when treatment units are selected in such a way, noting the possibility of bias in treatment selection.

### 5.5 Meta‐analysis of the effects of POP on crime and disorder

Our first meta‐analytic model presents the overall mean effects for 70 outcomes across the 34 included studies. As noted, above, many studies reported on multiple crime/disorder outcomes and for many the authors did not specify any one outcome as the primary target of their intervention. In our data, 13 of the 34 (38.2%) studies fit into this category. To avoid any “creaming” of results, we include all relevant outcomes from such studies. For studies with multiple outcomes, a mean effect size is used in this model; thus each study is only counted once in the analysis. This is the same approach used in our original review, as well as other recent Campbell reviews of policing strategies (e.g., Braga, Turchan, et al., 2019).

The results from the first model are presented in Figures 2a (Cohen's *D* model) and 2b (RIRR model). The forest plots show the standardized mean differences and log RIRRs, respectively, between the treatment and control groups, with the lines on either side representing the 95% confidence interval (CI). Effects to the right of 0 are supportive of reductions in crime/disorder, while effects to the left would suggest backfire effects where problems increased in the treatment areas relative to the controls. A random effects model was estimated based on an a priori assumption of a heterogeneous distribution of effect sizes (and the *Q* statistics for our models confirm this assumption).

Figure 2(a) Combined effect size for study outcomes: (a) Cohen's *D* (random effects model, *Q *= 165.177, *df *= 33, *p *< .001, *I* ^2^ = 80.021) and (b) Log RIRR (random effects model, *Q *= 218.963, *df *= 32, *p *< .001, *I* ^2^ = 85.386). CI, confidence interval

**Figure:**
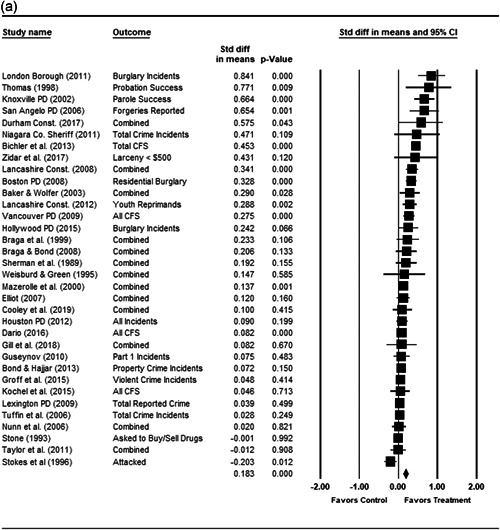

**Figure:**
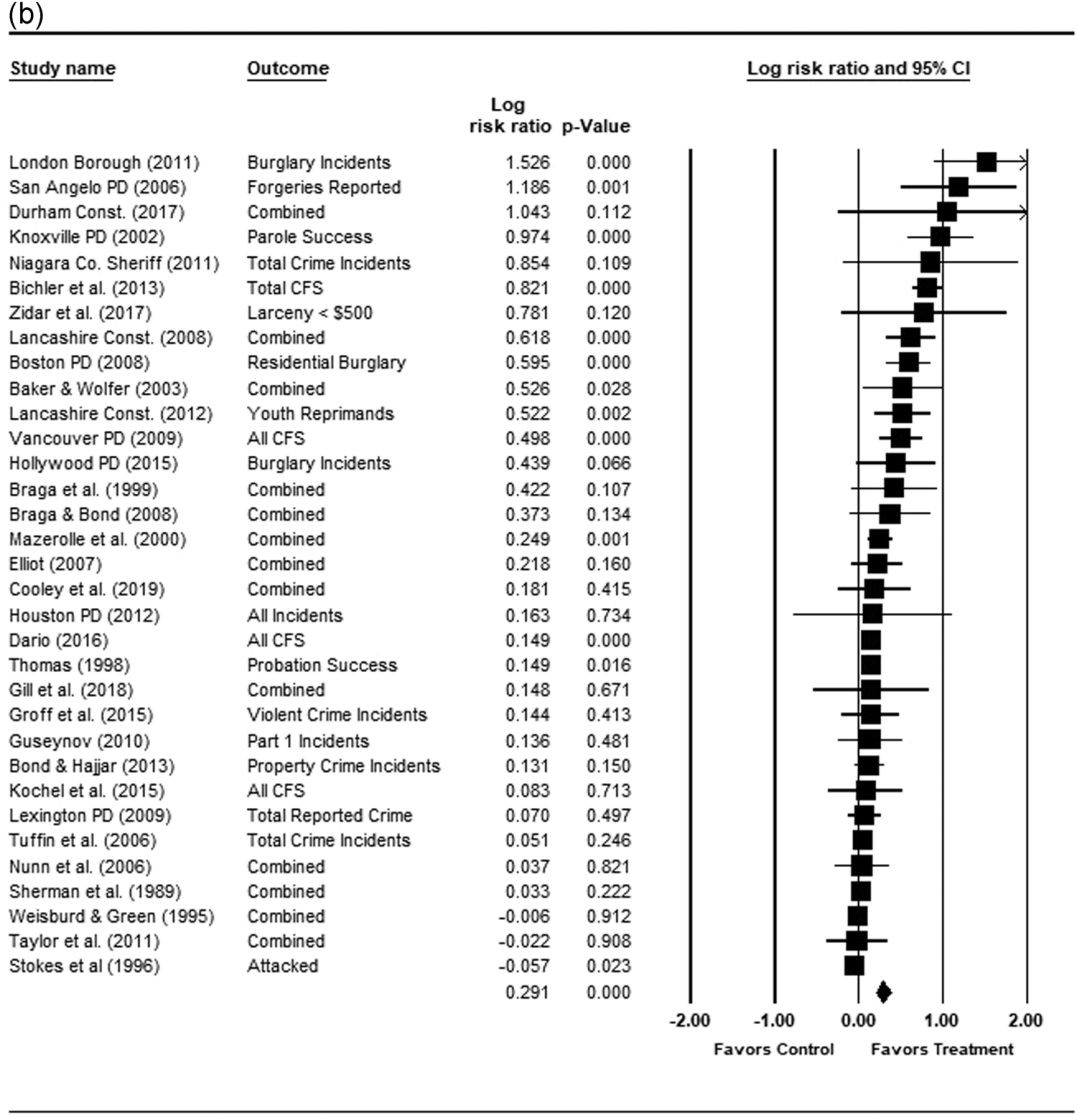

The overall mean effect size for Cohen's *D* approach is 0.183 (*p *< .001). The largest effects were found in the studies conducted by the London Borough Enfield (0.841), Thomas (0.771), the Knoxville Police Department (0.664) and the San Angelo Police Department (0.654)—all four were submissions for the Goldstein Award. Three studies reported negative overall effects, Stone (−0.001), Taylor et al. (−0.012), and Stokes et al. (−0.203).

The overall mean effect is considered a small effect by conventional standards developed by Cohen (1988). However, Lipsey (1990) describes effects in this range as small but meaningful impacts that could “easily be of practical significance” (Lipsey, 1990, p. 109). It is also important to note here that the studies clustered immediately around the mean effect size are randomized experimental evaluations of place‐based versions of POP in which the authors reported notable reductions in a variety of crime and disorder outcomes. (e.g., Weisburd & Green, 1995; ES = 0.147, Sherman et al., 1989; ES = 0.192; Braga & Bond, 2008; ES = 0.206; Braga et al., 1999; ES = 0.233). In this regard, Wilson (in progress) has raised strong concerns in interpreting place‐based effect sizes similarly to person‐based effect sizes. While we follow standard practice here in reporting effect sizes, we think that caution should be used in interpretation of what magnitudes mean.

For example, the largest impact in the study by Nunn et al. (2006) was an effect size of 0.200 for robbery calls for service (see Figure 3). This is an effect at the criterion of 0.20 set by Cohen (1988) for a small effect. Looking at the raw changes in the data, we see that the relative actual reduction in the proportion of crime in the treatment condition was 30.4% percent, while the control area saw no change in robbery calls. Similarly, in Gill et al.'s (2018) study the largest impact was on calls for service in the retail treatment site. Our effect size for this outcome of 0.187 is a bit below the 0.20 threshold, yet the raw change shows a 10.3% decrease in calls compared with a 25.9% increase in the comparison areas. Our point is that standard small effect sizes may translate to very meaningful crime prevention outcomes at places.

Figure 3Largest effect size for each study: (a) Cohen's *D* (random effects model, *Q  *= 489.197, *df  *=  33, *p *< .001, *I* ^2^ = 93.254) and (b) Log RIRR (random effects model, *Q* = 246.548, *df* = 32, *p* < .001, *I* ^2^ = 87.021). CI, confidence interval

**Figure:**
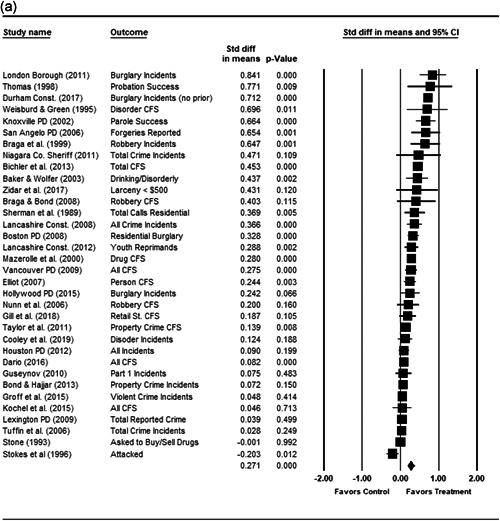

**Figure:**
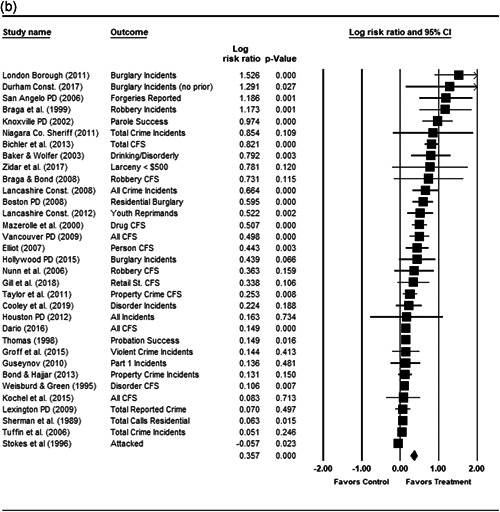

As noted above, David Wilson (in progress) has argued that Cohen's *D* fails to produce effect sizes that are comparable across studies when based on place‐based count data and has also shown that the process of converting RIRRs to Cohen's *D* is problematic. As such, we also present meta‐analyses for all of our models using the Log RIRR as the effect sizes. These analyses are of 33 of 34 included studies (and 69/70 outcomes). The study by Stone (1993) is not included in the RIRR models as the data and methods used did not allow us to calculate an RIRR. This is not a major concern as this study is a near zero effect in Cohen's *D* model (*D *= −0.001, *p* = .992). The overall RIRR model is shown in Figure 2b.

The results of the RIRR show an overall effect of 0.291. This can be interpreted as a relative present change by taking the exponent of the effect (which is a log RIRR) and then subtracting 1 from that value and multiplying by 100. For the overall model this shows that there was a 33.8% reduction in crime/disorder in the POP treatment areas/groups relative to the controls. Some caution is needed in interpreting this effect size as analyses presented below show smaller (though still positive and statistically significant) effects in randomized experiments and after accounting for publication bias. Nonetheless, this finding supports our illustration above of how Cohen's *D* often understates the magnitude of effects for place‐based studies (the majority of our sample) and provides further evidence that Wilson (in progress) is correct that Cohen's *D* is not the most appropriate effect size for these types of studies. We continue to present Cohen's *D* models throughout to allow for comparison to our prior review.

Regarding the meaning of effect sizes, we think it important to also note here that scholars have argued that approaches like POP that alter the characteristics of high‐crime places may reduce more crime in the long run than approaches such as temporarily increased police presence or crackdowns (Braga, Turchan, et al., 2019; Braga & Weisburd, 2010). This is the case as lasting changes to places made through identifying and solving problems may reduce crime over the long term through reducing opportunities for crime or other mechanisms (see above). Thus finding a relative reduction of 33.8% may be suggestive of even larger impacts on crime/disorder in targeted areas in the long run.

Moving beyond the overall effect size, perhaps the most striking finding is that the overall trend of mean effect sizes per study skews very heavily toward studies that produced findings in the direction of POP being effective. Specifically, 31 out of 34 studies (91.2%) in Cohen's *D* model have positive effect sizes, with 13 of them (38.2%) being statistically significant effects in favor of the treatment group. In the RIRR model, 30/33 studies (90.9%) reported positive impacts and 12 (36.4%) were statistically significant. Only one study (Stokes et al., 1996) had a statistically significant backfire effect, and that was a project that was plagued by implementation and research design limitations as reviewed in Section 5.3. Excluding this study from our overall model slightly increases the mean effect size from 0.183 to 0.195 in Cohen's *D* model and increased the relative reduction in crime from 33.8% to 36.6% in the RIRR model.

Before moving on, it is also important to recall that we had 15 eligible studies that are not included in any of our analyses as they lacked data needed to calculate effect sizes. Goldstein and Tilley Award submissions accounted for 13 of these 15 studies. As one would expect for programs submitted for award consideration, all 13 of these studies discuss findings favorable to POP's effectiveness. The other two studies (Carson & Wellman, 2018; Wolfe et al., 2015) failed to find evidence of positive impacts, but also did not note any backfire effects and pointed to challenges with fully implementing the interventions. As such, there is no reason to believe that the absence of these studies from our meta‐analyses would alter any of our conclusions.

Given that the overall models present mean effect sizes for studies reporting on multiple outcomes without a clear primary outcome specified, we felt it important to also estimate models including only the largest and smallest effect sizes for each study. For studies with a single outcome, or a clearly stated primary outcome, their effect sizes remain the same in all models. This approach provides an upper and lower bounds for the overall standardized mean effect. Figure 3a,b presents the largest effects models and Figures 4a,b the smallest effects models.

Figure 4Smallest effect size for each study: (a) Cohen's *D* (random effects model, *Q*  = 194.992, *df*  =  33, *p* < .001, *I* ^2^ = 83.076) and (b) Log RIRR (random effects model, *Q* = 232.501, *df * = 32, *p* < .001, *I* ^2^ = 86.237). CI, confidence interval

**Figure:**
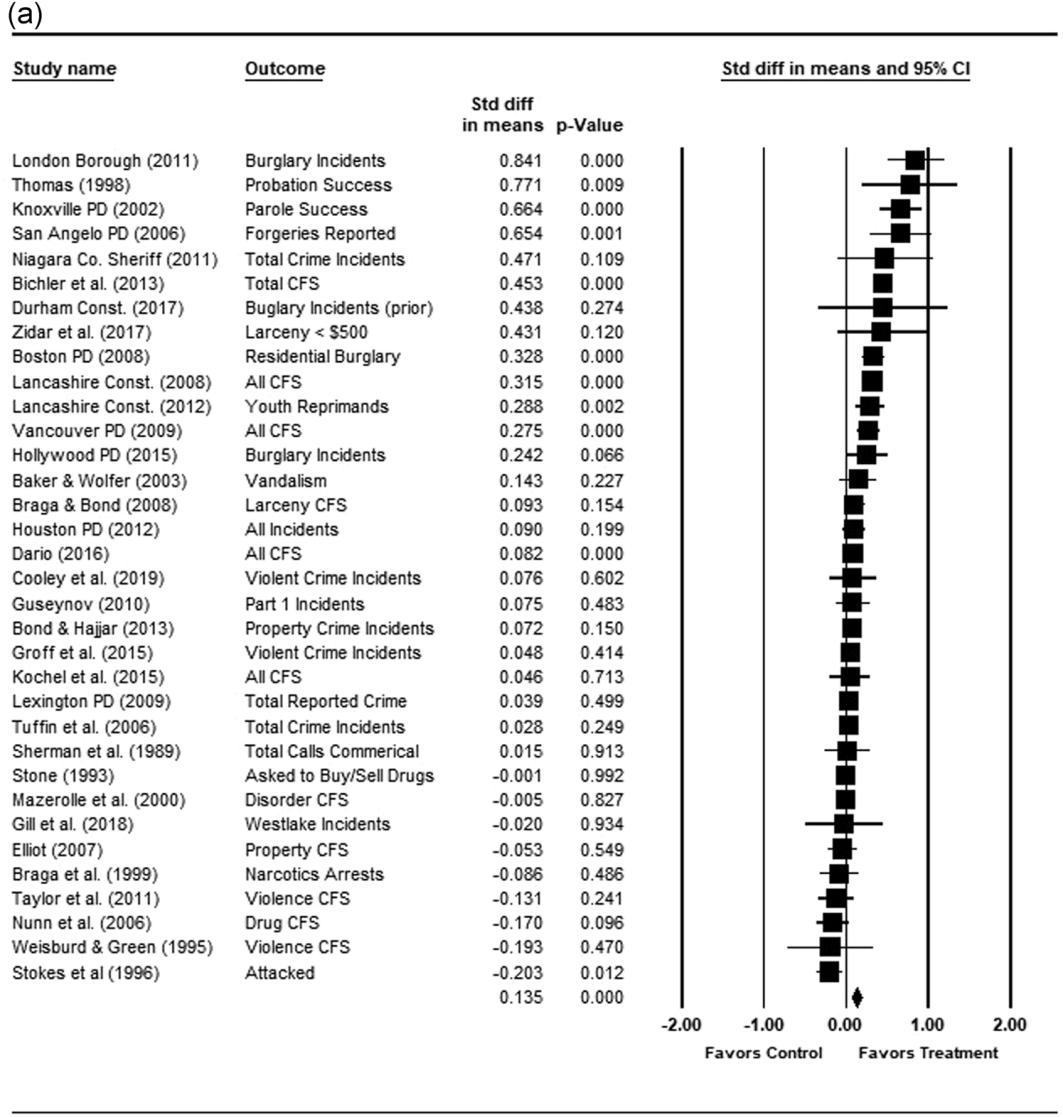

**Figure:**
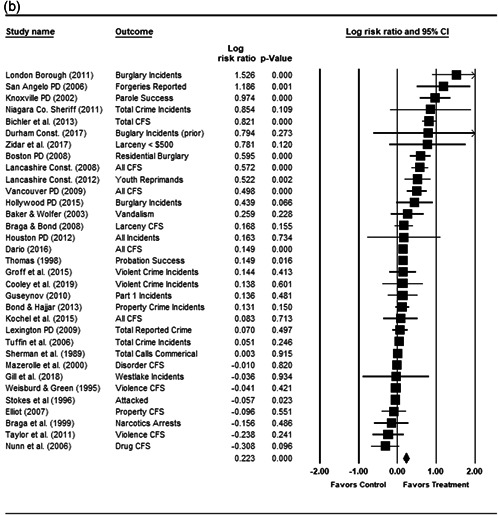

Following the logic of inclusion, the overall random effect is larger when only including the outcome with the largest effect size for each study. While the mean effects Cohen's *D* model had an overall effect of 0.183, the largest effects model has an overall standardized mean effect of 0.271 (*p* < .001)—an increase of 0.088 (48.1%). Similarly, for the RIRR model the mean effects approach had an overall effect of 0.291 (33.7% relative reduction), the largest effects model had an overall effect of 0.357. This corresponds to a 42.9% relative reduction of crime/disorder in the treatment group.

Turning to the smallest effects, we see a lower bound for the standardized mean effect of 0.135 (*p* < .001)—a decrease of 0.048 (−26.2%) from the overall mean effect model. For the RIRR model we see a lower bound of the overall effect of 0.223, corresponding to a 25.0% relative reduction in crime/disorder. We think that this approach gives a sense of the range of effects that can be expected in POP programs. Specifically, using the RIRR results, the overall effect ranges from a 25% relative reduction when using the smallest effects to 42.9% relative reduction when using the largest effects. Importantly, our overall conclusion about the effectiveness of POP remains consistent. Regardless of the type of effect size and whether we examine the overall mean effect or look at the largest or smallest effect size, the results suggest that POP has a significant meaningful effect (Lipsey, 1990) in reducing crime/disorder.

Finally, we felt it important to examine variation in effect sizes across crime types. Table 5 summarizes the mean effects sizes for violent crime, property crime and disorder offenses for both our Cohen's *D* model and the RIRR approach. Studies that reported on aggregated crime counts that included more than one of these categories are not included in these analyses, nor are other types of outcomes that did not fit into those groupings (and lacked enough cases to perform a meaningful meta‐analysis).

**Table 5:** The effects of problem‐oriented policing on specific crime types—mean effects

Crime type	Studies (*N*)	Mean effect, Cohen's *D*	Mean effect, Log RIRR (relative change)	*Q* Statistic (heterogeneity)^a^
Violent crime	9	0.066	0.091 (9.5%)	20.435** (*df *= 8)
Property crime	12	0.171**	0.270 (31.0%)**	49.765** *(df *= 11)
Disorder offenses	7	0.173**	0.173 (18.9%)**	16.895** (*df *= 6)

** *p* ≤ .01.

*** *p* ≤ .001.

^a^ *Q* statistics are from the Log RIRR models. Those for Cohen's *D* models are substantively identical and not presented.

The results show that while POP had significant impacts on property crime (31.0% relative reduction) and disorder offenses (18.9% relative reduction), the overall effect for violent crime did not reach statistical significance (*p *= .156 in the RIRR model, *p *= .218 in Cohen's *D* model). However, the effect is still in the positive direction (9.5% relative reduction) and 13 of the 18 violent crime outcomes were positive. Future research should further explore the potential reasons for the heterogeneous impact of POP across crime types.

#### 5.5.1 Moderator analyses

We also conducted moderator analyses to examine heterogeneity in effect sizes across three dimensions—(a) experiments versus quasi‐experiments, (b) studies with nonequivalent groups versus all others, and, (c) the type of publication (scholarly publications vs. Goldstein Award submissions).

Study design is important to assess as it is well known that more rigorous designs are more likely to produce null findings (Rossi, 1987). Figure 5a,b shows the moderator results for randomized experiments versus quasi‐experiments.

Figure 5Research design as a moderator for study outcomes. (a) Cohen's *D*. Random effects model, quasi‐experiments: *Q* =  160.384, *df *= 24, *p* < .001; randomized experiments: *Q* = 4.773, *df *= 8, *p* < .782; between groups: *Q *= 4.914, *df *= 1, *p *= .027. While the *Q* statistic is not significant in the randomized experiments model, the random effects and fixed effect model results are identical for this subsample. (b) Log RIRR. Random effects model, quasi‐experiments: *Q* = 203.223, *df* = 23, *p* < .001; randomized experiments: *Q* = 12.186, *df* = 8, *p* < .143; between groups: *Q* = 14.171, *df* = 1, *p *= .001. While the *Q* statistic is not significant in the randomized experiments model, the random effects and fixed effect model results are very similar for this subsample). CI, confidence interval

**Figure:**
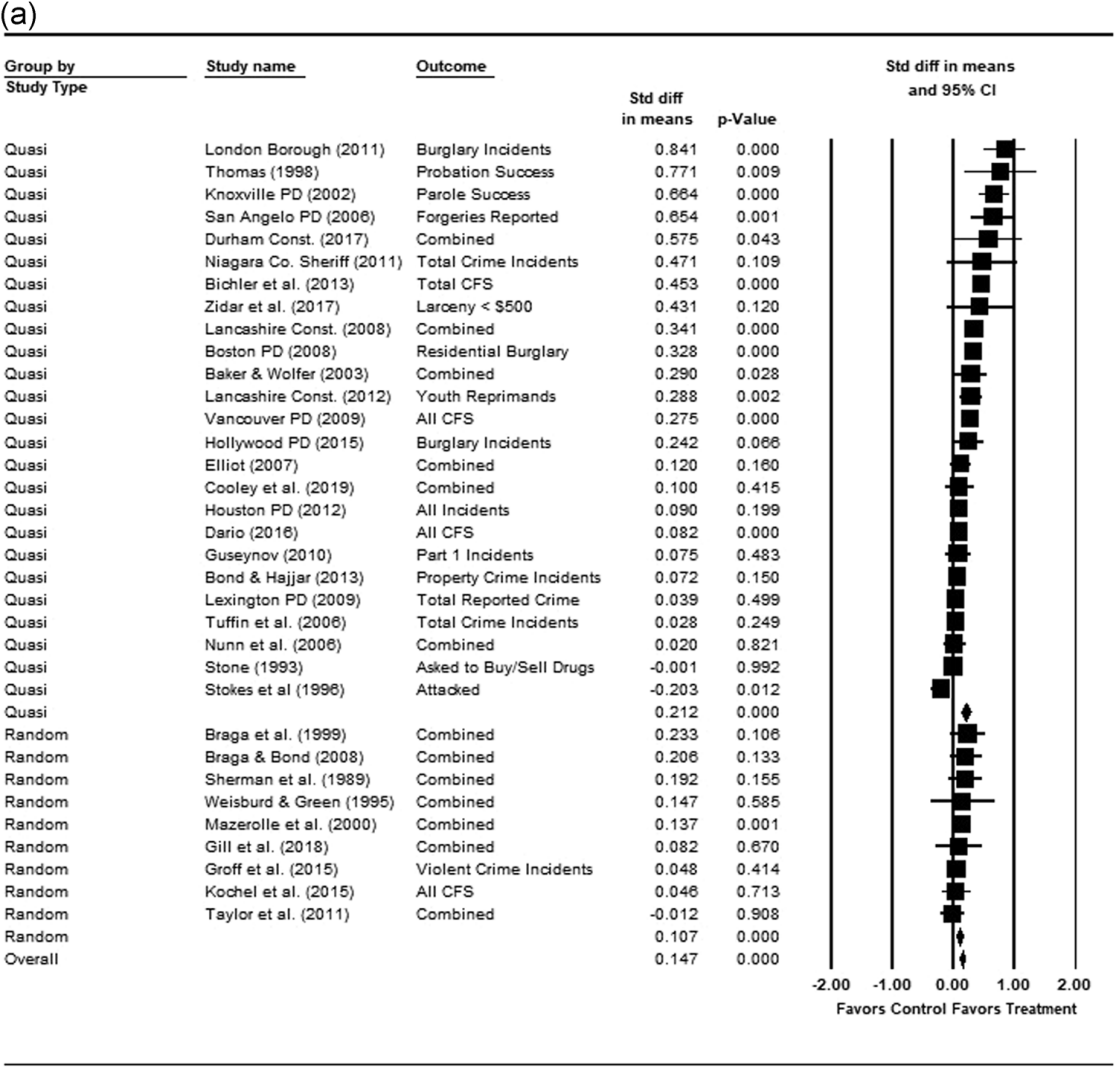

**Figure:**
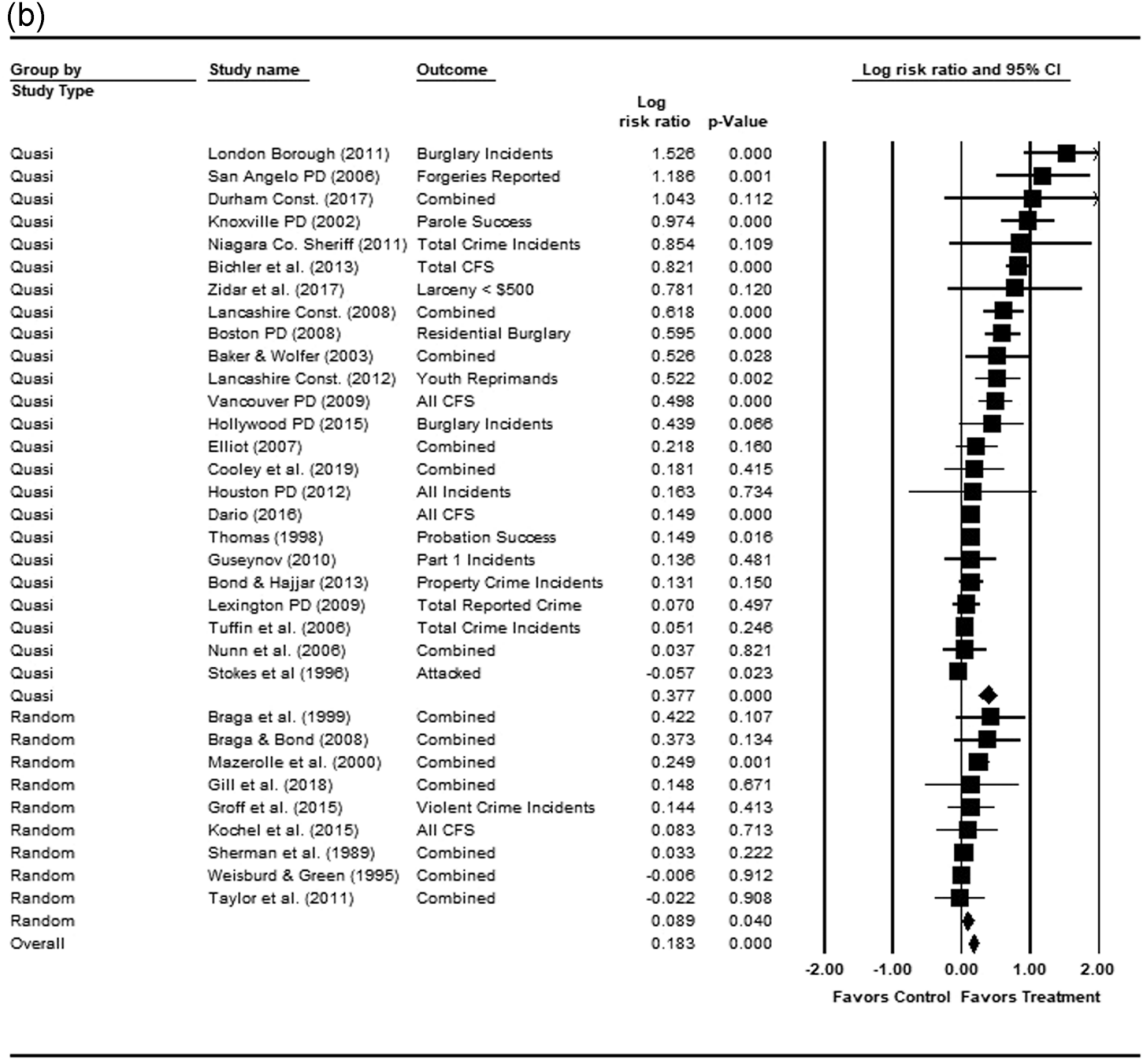

The moderator analysis of the impact of study design shows that quasi‐experiments have a larger overall effect size than randomized experiments. For Cohen's *D* model (see Figure 5a) the quasi‐experimental studies have an effect size of 0.212 (*p *< .001), while the randomized experiments have an effect size of 0.107 (*p *< .001). The difference between groups was statistically significant (*Q *= 4.914, *df *= 1, *p *= .027) and the moderated effect size is 0.147 (*p *< .001). Turning to the RIRR models (see Figure 5b) we see the same pattern. For quasi‐experiments, the effect of 0.377 (a relative reduction of 45.8%) was larger than the effect of 0.089 (a relative reduction of 9.3%) for randomized experiments. The difference between groups was again statistically significant (*Q *= 14.171, *df *= 1, *p *< .001) and the moderated effect size was 0.183—a relative reduction of 20.1%.

These results are consistent with Weisburd, Lum, and Yang's (2003) proposal that experimental designs more generally show smaller impacts in crime and justice research (see also, Welsh, 2016). The results show that while there is a bias toward finding stronger effects in studies with weaker research designs, the overall finding of a significant meaningful effect for POP is supported across study types, as well as by the moderated effect size.

Our second methodological moderator analysis examined the impact of the nonequivalent research designs highlighted in Table 4. Specifically, we compared the studies that are listed in Table 4 as having nonequivalent control groups to studies with better matching methods. The results here suggest that studies with nonequivalent control groups did not bias our conclusions. Indeed, effect sizes were actually slightly larger in the studies with better matching methods. For Cohen's *D* models, the 11 studies with nonequivalent control groups had an overall effect size of 0.178 (*p *< .001), while the effect for the 23 studies with more rigorous matching approaches was 0.190 (*p *< .001). The difference between groups was not statistically significant (*Q *= 0.034, *df *= 1, *p *= 0.854) and the moderated effect size was 0.184 (*p *< .001). Similarly, for the RIRR models, the nonequivalent control groups had an overall effect size of 0.263 (*p *< .001; a 30.1% relative reduction), while the studies which used better matching methods had an effect of 0.309 (*p *< .001; a relative reduction of 36.2%). The between‐groups difference was again not statistically significant (*Q *= 0.231, *df *= 1, *p *= .631) and the moderated effect size was 0.289 (*p *< .001; a 33.5% relative reduction).

Next, we examined the impact of the type of publication. This is important as the award submissions are inherently biased toward successful outcomes and likely also toward larger effects. The reasoning here is simple—police departments are not going to submit a program that did not work for consideration for an award and are probably most likely to submit when a project has a larger impact. As such, our moderator analysis here compares the mean effects for the award submissions to those for scholarly publications (journal articles, research reports, theses and dissertations in our current sample). These results are shown in Figure 6a,b.

Figure 6Publication type as a moderator for study outcomes (a) Cohen's *D* (random effects model, award submissions: *Q *= 52.115, *df *= 12, *p *< .001; scholarly publications: *Q *= 80.200, *df *= 20, *p *< .001; between groups: *Q *= 13.702; *df *= 1; *p *< .001) and (b) Log RIRR (random effects model, award submissions: *Q* = 58.089, *df* = 12, *p* < .001; scholarly publications: *Q* = 113.161, *df* = 19, *p* < .001; between groups: *Q* = 12.329; *df* = 1; *p* < .001). CI, confidence interval

**Figure:**
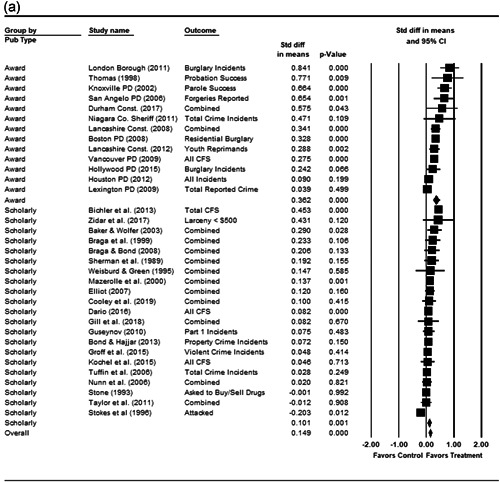

**Figure:**
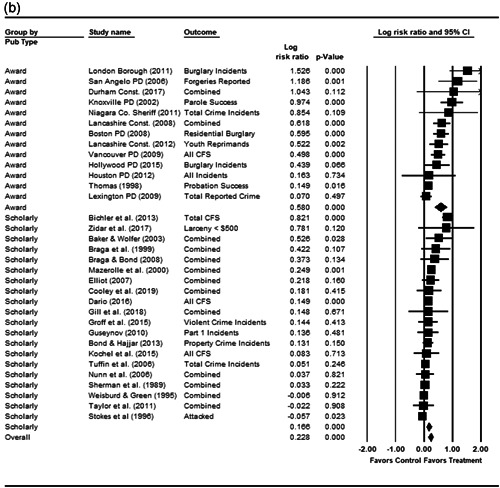

As expected, the award submissions have a larger overall mean effect size than scholarly publications. Examining Cohen's *D* model (Figure 6a) we see that the award submissions had an overall effect of 0.362 (*p *< .001) while the effect for scholarly publications was 0.101 (*p *= .001). The difference between groups was statistically significant (*Q *= 13.702, *df *= 1, *p* < .001) and the moderated effect size is 0.149 (*p *< .001). The results from the RIRR model (Figure 6b) are very similar, with the effect for award submissions being 0.580 (a 78.6% relative reduction) compared with an effect of .166 (an 18.1% relative reduction) for the scholarly publications. The between‐groups difference was again statistically significant (*Q *= 12.329, *df *= 1, *p* < .001) and the moderated effect size was 0.228 (a 25.6% relative reduction).

These results raise possible concerns regarding the exclusion of police‐initiated POP programs that were evaluated in some way but were not submitted for award nominations. We conduct analyses regarding publication bias below, and note the biases there. Nonetheless, because our analyses without the award submissions remain statistically significant, this finding does not alter our overall conclusion of a significant crime prevention outcome for POP. In turn, these award studies do provide additional information about successful interventions and a sense of the upper range of POP impacts that are to be expected.

As a final summary and sensitivity analysis, Tables 6a and 6b below summarize the main effect sizes outlined above, and also present results from models using the smallest and largest effects in each applicable study for randomized experiments, quasi‐experiments, award submissions and scholarly publications that were not presented above to save space. The range of effects support our conclusion of a meaningful effect of POP in reducing crime in all but two of the models. The overall effect is not significant in the two most conservative models—smallest effect outcomes for randomized experiments and scholarly publications—for both Cohen's *D* and RIRR models. All the other models produced statistically significant overall effects. For Cohen's *D* models (see Table 6a), those effects ranged from 0.101 (mean effect for randomized experiments) to 0.415 (largest effect outcomes for award submissions). The mean effects shown in the “Combined ES” column provide the overall summary of effects across all outcomes for each model and show that across all models the average overall effect of POP on crime/disorder ranges from 0.101 to 0.362. Similarly, looking at the RIRR models summarized in Table 6b (again excluding the smallest effects for the randomized experiments and scholarly publications which are not significant) shows that crime in the POP group relative to the controls was reduced between 9.3% (mean effect for randomized experiments) and 81.5% (largest effect for award submissions). The mean effect ranged from the 9.3% relative reduction in randomized experiments to 78.6% for award submissions.

**Table 6a:** Summary of effect sizes ranges across models (Cohen's *D*)

Model	Smallest ES	Combined ES	Largest ES
All studies	0.135***	0.183***	0.271***
Randomized experiments	0.005	0.107***	0.229***
Quasi‐experiments	0.189***	0.212***	0.276***
Scholarly publications	0.039	0.101***	0.167***
Award submissions	0.350***	0.362***	0.415***

*** *p* ≤ .001.

**Table 6b:** Summary of effect sizes ranges across models (Log RIRR)

Model	Smallest ES	Combined ES	Largest ES
All studies	0.223 (25.0%)***	0.291 (33.8%)***	0.357 (42.9%)***
Randomized experiments	−0.003 (−0.3%)	0.089 (9.3%)*	0.241 (27.3%)***
Quasi‐experiments	0.337 (40.1%)***	0.377 (45.8%)***	0.419 (52.0%)***
Scholarly publications	0.080 (8.3%)	0.166 (18.1%)***	0.256 (29.2%)***
Award submissions	0.566 (76.1%)***	0.580 (78.6%)***	0.596 (81.5%)***

* *p* ≤ .05.

*** *p* ≤ .001.

As such, our review provides strong and consistent evidence that POP is an effective approach to reducing crime and disorder. However, there is a great deal of heterogeneity in the magnitude of effect sizes across factors such as study type, study rigor, and crime type. This will be discussed more in the discussion and conclusion sections of this report.

#### 5.5.2 Meta‐analysis of displacement and diffusion effects

Many of our studies were place‐based approaches to POP that may have had potential to either displace crime/disorder to surrounding areas or to see the benefits of the intervention diffuse to areas that were not targeted (see Weisburd et al., 2006). Eight of our studies provided data for a total of 19 outcomes that allowed us to create effect sizes and conduct a meta‐analysis of displacement and diffusion effects.

**Figure 7:**
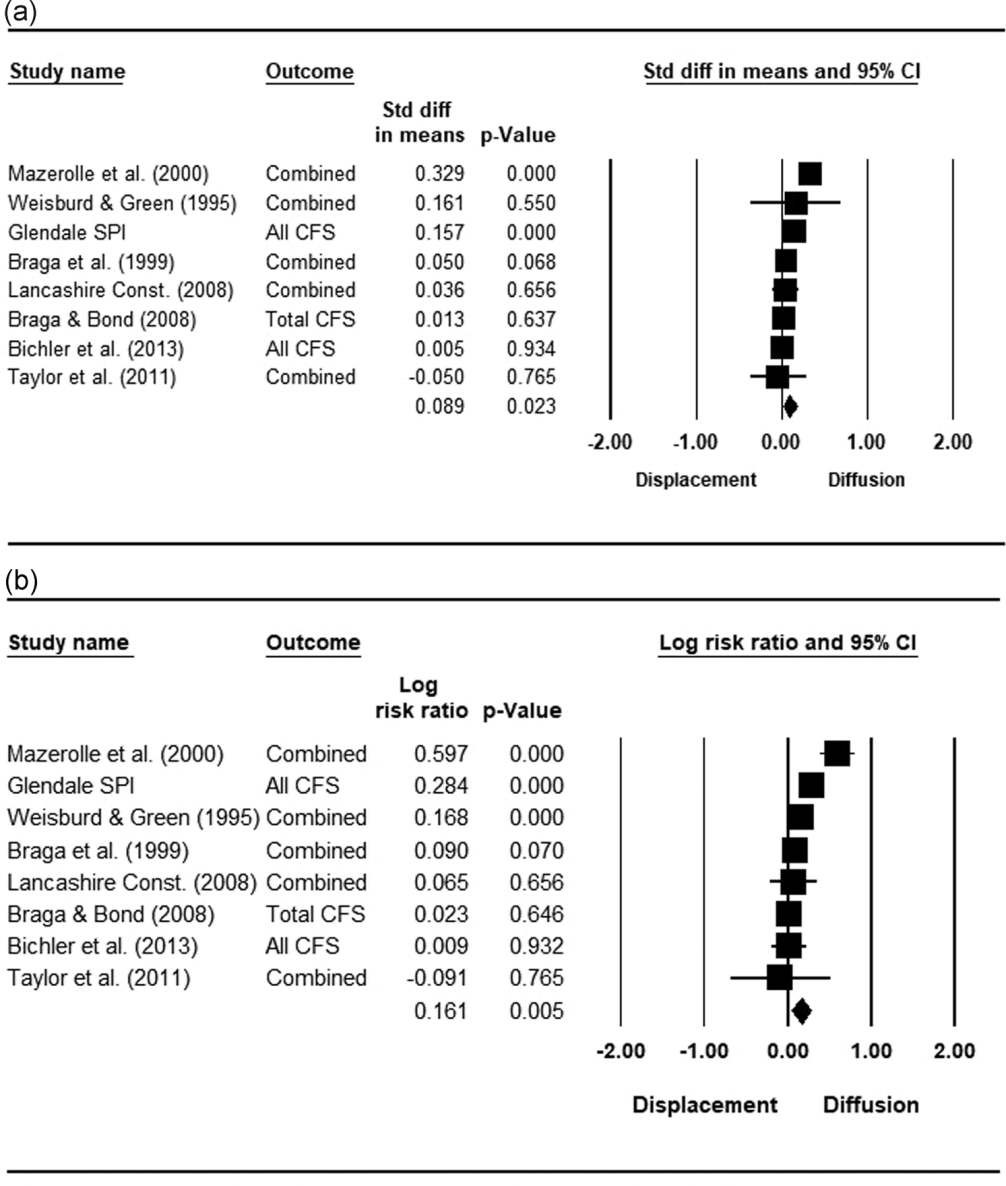
Displacement and diffusion‐combined effect size for study outcomes (a) Cohen's *D* (random effects model: *Q *= 30.069, *df *= 7, *p *< .001, *I* ^2^ = 76.720) and (b) Log RIRR (random effects model: *Q *= 30.547, *df* = 7, *p* < .001, *I* ^2^ = 77.085). CI, confidence interval

These effect sizes were calculated using pre‐ and postintervention counts for target, control, and buffer areas. This was done following the approach used by Telep, Weisburd, Gill, Vitter, and Teichman (2014) in their meta‐analysis of displacement and diffusion effects of interventions in large‐scale geographic areas (see also Bowers, Johnson, Guerette, Summers, & Poynton, 2011; Braga, Turchan, et al., 2019). Effect sizes were calculated using the relative incidence risk ratio approach described above. Pre‐ and postintervention counts from the treatment buffer area(s) are compared with pre‐ and postintervention counts either from a control buffer area or to the control area itself in studies that did not have a catchment area for the control site. Figure 7 shows the mean overall effect for displacement/diffusion. Effects to the right of zero indicate evidence of diffusion of crime control benefits, while effects to the left suggest displacement effects.

Looking at Cohen's *D* model (see Figure 7a), the overall model provides no evidence of displacement. Seven of the eight studies have positive effect sizes, while the sole negative effect for Taylor et al. (2011) was very small and not statistically significant (−0.050, *p *= .765). Moreover, the overall random effect for the model of 0.089 (*p *= .023) is suggestive of diffusion of benefits across these eight studies, though caution is needed given the small effect size and limited number of studies. We also estimated displacement/diffusion models using the largest and smallest effects to provide a range for our overall estimate. This showed that when using the smallest effects, there is no evidence of either displacement or diffusion (0.029, *p *= .345), while when only including largest effects the evidence in favor of diffusion of benefits is moderately stronger than in the mean effects model (0.154, *p *= .006). The results from the RIRR model lead to the same conclusions. The overall model (see Figure 7b) shows a relative reduction of 17.5% (*p *= .005) in favor of diffusion effects and the largest effects model shows a larger relative decline of 34.6% (*p *= .004). The smallest effects model shows no statistical evidence of displacement or diffusion, with a nonsignificant relative difference of 4.6% (*p *= .327) in favor of diffusion.

#### 5.5.3 Narrative review of impacts on noncrime/disorder outcomes

While the primary question of our review is concerned with crime and disorder outcomes of POP, we also collected data, when available, on the cost effectiveness of POP, as well as secondary outcomes of POP programs, including impacts on police legitimacy, fear of crime, and collective efficacy. Because only a small number of included studies focused on each of these outcomes and inconsistency in measures across studies, we felt a narrative review of findings would be more useful for synthesis than a meta‐analysis. Additionally, because our included studies generally prioritized crime control outcomes, the information provided on these secondary outcomes is not always sufficient for calculating effect sizes. We are also more cautious in interpreting these findings, since we did not search for, and excluded any studies we did find, that focused exclusively on fear of crime or other noncrime outcomes. A future study should systematically search for studies of POP focused on impacting outcomes other than crime and disorder.

We include summary information on each outcome below and an examination of findings by study in Table 7. Our findings overall suggest POP can be cost‐effective, but tends to have limited impacts on police legitimacy, fear of crime, and collective efficacy, although those outcomes are often not assessed in our included studies.^13^

**Table 7:** Impacts of problem‐oriented policing noncrime/disorder outcomes

Study	Cost‐benefit	Police legitimacy/satisfaction	Fear of crime	Collective efficacy
Baker and Wolfer (2003)	Not measured	Not measured	Two‐wave survey of park residents (wave 1 = 124, wave 2 = 125) versus general borough residents (Near park (wave 1 = 337, wave 2 = 333) Greater increase in feeling safe in park during day for target residents (from 63.1% to 92.9%) versus control (from 78.3% to 84.4%) Greater increase in feeling safe due to crime prevention in control residents (from 56.1% to 88.6%) versus target (57.3% to 84.1%)	Not measured
Bichler et al. (2013)	Patrol officer time on motels drops from average of 2,744 hr per year pre‐ordinance (2001–2006) to 1,448 hr per year during ordinance (2007–2009) City agency hours spent on motels drop from 184.8 per year pre‐ordinance to 92.4 per year during ordinance	Not measured	Not measured	Not measured
Bond and Hajjar (2013)	Not measured	Not measured	Not measured	Not measured
Boston Police Department (2008)	Not measured	Not measured	Not measured	Not measured
Braga et al. (1999); Braga (1997)	Not measured	Not measured	Only measured in treatment group	Not measured
Braga and Bond (2008, 2009)	Not measured	Pre–post interviews with 52 key stakeholders	No pre to post change in fear of victimization for violent and property crime in treatment relative to control hot spots	Not measured
		Treatment group interviewees saw increase in police presence, but no difference in policing style, demeanor of police, or willingness to work with citizens in treatment relative to control hot spots		
Cooley et al. (2019)	Not measured	Pre‐ and postsurvey (pre: *n* = 79, post = 89) in treatment and control neighborhoods No difference over time or across neighborhoods in perception of police doing a good job	No difference over time or across neighborhoods in whether feel safe walking alone at night or afraid of being a victim	Not measured
Durham Constabulary (2017)	£5,000 spent on crime prevention products Average cost of dwelling burglary is £576. 15 fewer dwelling burglaries in the target areas than the control areas suggest cost savings of £3,640. Savings greater if considering impacts of burglary on all criminal justice agencies and victims (average cost £3,266).	Not measured	Not measured	Not measured quantitatively
Elliott (2007); Reno Police Department (2006)	Estimates project call reduction has saved about 1,750 officer hours per year	Not measured	Not measured	Not measured
Gill et al. (2018)	Not measured	Not measured	Not measured	Not measured
Groff et al. (2015); Ratcliffe et al. (2015)	Not measured	Pre–post mail survey (pre = 157 POP residents, 159 control residents; post = 162 POP residents, 177 control residents) No change over time or between POP and control residents in satisfaction with police services and perceptions of police procedural justice	No difference between POP and control over time in perceptions of safety	Not measured
Guseynov (2010)	Not measured	Not measured	Not measured	Not measured
Hollywood Police Department (2015)	Not measured	Not measured	Not measured	Not measured
Houston Police Department (2012)	Not measured	Not measured	Not measured quantitatively	Not measured quantitatively
Knoxville Police Department (2002)	Not measured quantitatively	Not measured	Not measured	Not measured
Kochel et al. (2015); Kochel and Weisburd (2017, 2019)	Not measured	Three wave survey (pre: *n* = 266 POP, 454 control; post 1: *n *= 223 POP, 331 control; post 2: *n* = 311 POP, 468 control) Improvement in procedural justice in POP over time, but no greater than control Slight nonsignificant decline in legitimacy in POP in short‐term relative to control, but rebounds by second postsurvey	POP and control residents saw increase in perceived victimization risk in short‐term, no differences in long‐term Significant decline in feelings of personal safety in POP relative to controls in short‐term, no significant difference in long‐term	Significant improvement in long‐term (but not short‐term) in informal social control in POP relative to control (7% relative to baseline) No improvement in social cohesion or overall collective efficacy in POP
Lancashire Constabulary (2008)	Average annual burglary costs per year go from $112,700 in pre to $50,600 in post Average criminal damage costs go from $124,440 in pre to $52,020 in post Average antisocial behavior costs go from $128,650 in pre to $76,880 in post	Not measured	Not measured quantitatively	Not measured quantitatively
Lancashire Constabulary (2012)	Reduction in arrests estimated to save £ 82,000 (cost processing arrest estimated as £2,000; drop from 65 arrests in 2008 and 2009 to 24 arrests in 2010 and 2011)	Not measured	Not measured	Not measured
Lexington Division of Police (2009)	Not measured	Not measured	Not measured	Not measured
London Borough of Enfield (2011)	Funding for the crime prevention = £231,000 Estimate of system and social savings from burglaries prevented = £934,000	Only measured in treatment group	Only measured in treatment group	
Mazerolle et al. (2000)	Not measured quantitatively	N/A	Not measured as outcome	Not measured as outcome
Niagara County Sheriff's Office (2011)	Not measured quantitatively	Not measured	Not measured	Not measured
Nunn et al. (2006)	Not measured	Not measured	Not measured	Not measured
San Angelo Police Department (2006)	Not measured	Not measured	Not measured	Not measured
Sherman et al. (1989)	Not measured	Not measured	Not measured	Not measured
Stokes et al. (1996)	Not measured	Not measured	Pre–post victimization survey (pre: *n *= 514 in target school, 1,988 in controls; post: *n* = 414 in target, 1,721 in controls) asked students their fear of being attacked In target school, fear increased from 32.4% in pre to 33.4% in post, while decreasing from 30.4% to 28.9% in control schools	Not measured
Stone (1993)	Not measured	Pre–post survey in two treatment (*n* = 116, pre; *n* = 91, post) and two control (*n* = 147, pre; *n* = 97 post) public housing sites Examined satisfaction with police (overall, see police, police treat residents with respect, police enforce laws) No impact between sites or over time	Fear of crime (level of concern about burglary, violence in the street, violence in the home, children getting involved in drugs)	Informal social control (what would neighbors do if house burglarized or being attacked)
				No impact between sites or over time
			No impact between sites or over time	
Taylor et al. (2011)	Not measured	Not measured	Not measured	Not measured
Thomas (1998)	Not measured	Only measured in treatment group	Not measured	Not measured
Tuffin et al. (2006)	Not measured	Baseline and follow‐up survey, approximately 200 in each wave in each treatment and comparison area Satisfaction with police from direct contact had too small of a sample for significance testing, but treatment areas do show 8% increase pre to post Confidence in policing—large overall program effect, 12% net increase in treatment sites in those who felt police doing an excellent or great job Police willing to listen and respond—8% net increase in treatment sites	No consistent effects in fear of crime types; small net impact for treatment sites in fear of being physically attacked by strangers 5% net improvement in feelings of safety at night in treatment sites	No impact on social cohesion and perceptions of whether respondent is in a close, tight‐knit community No impact on overall collective efficacy and whether residents would intervene if young people were causing trouble or residents helping each other out Net 5% increase for treatment sites for trusting many/some of the people in their area
Vancouver Police Department (2009)	Not measured	Only measured in treatment group	Not measured quantitatively	Not measured
Weisburd and Green (1995)	Not measured	Not measured	Not measured	Not measured
White and Katz (2013); Dario (2016)	Each Circle K call took average of 23 min at $46.26 per hour for officer time Preintervention calls cost $43,686, in officer time versus $25,403 postintervention Conservative estimate as only includes patrol costs	Not measured	Not measured	Not measured
Zidar et al. (2017)	Department spent at least $26,884 less on Walmart‐related costs in year after intervention Spent about 35 hours less per month answering calls, average of $1,807 savings in officer time per month	Not measured	Not measured	Not measured

##### Financial cost‐benefit analysis

Eight studies assessed cost or hours savings as a result of the POP project. These were generally based on cost estimates for how much time would have been spent on calls for service or incidents that were prevented by the POP project. In most cases, estimates were just based on police time and cost, but two studies (Bichler et al. 2013; London Borough of Enfield, 2011) also included estimates for time saved by other agencies. In all cases, the POP project was associated with a substantial cost savings. We recognize though that POP projects without significant impacts on crime would be less likely to include a cost‐benefit analysis.

Two motel‐based studies in the U.S. looked just at hours saved. Bichler et al. (2013) examined just savings in hours, finding the motel ordinance program saved 1,253.4 hours per year in patrol time on calls for service, more than a 51% reduction. Time spent by other city agencies on motel‐related issues also dropped 92.4 hours per year, on average. The Reno Police Department (2006) did not provide precise estimates for their entire project, but noted that impacts had saved the department approximately 1,750 officer hours per year.

Two other U.S. studies estimated cost savings based on calls prevented in retail locations. White and Katz (2013) estimated the cost to respond to calls at targeted convenience stores dropped from $43,685 to $25,403. They argue these are conservative estimates, since they do not account for other criminal justice system and business costs. Zidar et al. (2017) found a program to reduce responses for low‐level theft led to $26,884 less in police department manpower costs. The department saved about 35 hours per month by responding to fewer calls at Walmart.

Four studies by U.K. agencies also estimated savings, using Home Office estimates on the costs of crime. Durham Constabulary (2017) found that burglaries prevented equated to a savings of £3,640 just in police‐related costs, with even higher estimates when accounting for all system and victim costs. Lancashire Constabulary (2008) estimated cost savings across multiple crime categories as a result of their POP project. Burglary savings were estimated at $62,100, criminal damage savings were $72,420, and antisocial behavior incident savings totaled $51,770. A second project by Lancashire Constabulary (2012) found a significant reduction in arrests were associated with a total cost savings of £82,000. The London Borough of Enfield (2011) estimated project cost savings at £934,000, accounting for both police and social costs of crimes prevented.

##### Police legitimacy/satisfaction

Six studies used measures of resident perceptions of police procedural justice and/or legitimacy to assess impacts of POP on trust in the police. Results here were not entirely consistent, but generally suggest POP has limited impact on police legitimacy. There is no evidence here, however, that problem‐oriented approaches, even when applied in hot spots, damage police trust.

Stone (1993) saw no change over time or across treatment and control public housing sites in Atlanta in whether residents were satisfied with police and thought police treated them with respect. Braga and Bond (2008) found no differences in perceptions of police in pre and post interviews with respondents who were likely to have had contact with police during the Lowell hot spots experiment. Cooley et al. (2019) similarly found no change over time or between treatment and control neighborhoods in perceptions of whether police are doing a good job. Results were similar in the two most rigorous assessments. Using a mail survey, Ratcliffe et al. (2015) found no differences in perceptions of procedural justice or satisfaction with police among residents of control spots versus those receiving POP in Philadelphia. Using an in‐person resident survey, Kochel and Weisburd (2017) found procedural justice perceptions improved over time in problem solving hot spots, but no more than they did in control hot spots. There was a small nonsignificant decline in problem solving hot spot resident perceptions of legitimacy in the short‐term follow‐up, but legitimacy had rebounded to similar levels to control respondents by the long‐term follow‐up. Tuffin et al. (2006) found the only evidence of enhanced perceptions of police, although we note that this was an evaluation of reassurance policing, so building trust was a major component along with problem solving. Here, treatment site residents relative to control site residents saw 15% net improvements in confidence in police and 8% net increases in feeling that police are willing to listen and respond.

##### Fear of crime

Eight studies assessed changes in resident fear of crime as a result of a POP project. Findings here were not entirely consistent, but most studies found no impact of POP on resident fear of crime, and for studies that did find an impact, effects were generally small. Stone's (1993) public housing surveys, Braga and Bond's (2009) resident interviews, Ratcliffe et al. (2015) mail surveys, and Cooley et al's. (2019) resident surveys, for example, suggested no pre–post change in fear in treatment relative to control sites. Tuffin et al. (2006) saw limited impacts of reassurance policing on fear of crime. There was some net improvement in feelings of safety after dark in treatment compared with control sites, but for particular crime types, there were no consistent effects. Stokes et al. (1996) saw, if anything, negative impacts of the safe route to school program on student fear of being attacked, not surprisingly given the overall backfire effects of the intervention. Similarly, Kochel et al. (2015) found increases in victimization risk and decreases in feelings of personal safety among residents of problem solving hot spots relative to controls in the short‐term, but there were no differences across groups by the second postintervention survey. Baker and Wolfer (2003) found more substantial impacts of the intervention on fear of crime among residents living near the target site, particularly for feelings of safety in the park during the day; however, even here the findings were mixed. Control group respondents were more likely than treatment group respondents to say they felt safe due to crime prevention efforts in the postsurvey, even though only treatment group residents had received a crime prevention intervention.

##### Collective efficacy

Three studies included pre‐ and postintervention measures of resident perceptions of collective efficacy. Findings across the three studies were inconsistent and mixed. None of the studies showed large impacts of POP on collective efficacy. Stone's (1993) survey showed no difference in perceptions of informal social control over time or in treatment versus control housing complexes. Tuffin et al. (2006) also found no difference in collective efficacy perceptions among residents of reassurance policing areas relative to control sites. There were also no significant changes in perceptions of social cohesion, but treatment sites did show improvements relative to comparison sites in the percentage of residents saying they trust many or some people in their area. Kochel and Weisburd (2019) found no overall change in resident perceptions of social cohesion or overall collective efficacy in problem‐solving hot spots relative to controls. They did find some long‐term improvements in perceptions of informal social control among POP hot spot residents, with about a 7% improvement compared with baseline levels. Kochel and Weisburd (2019) suggested the limited community involvement in most implemented problem‐solving projects may explain the modest impacts.

#### 5.5.4 Publication bias

Publication bias presents a strong challenge to any review of evaluation studies (Rothstein, 2008). Campbell reviews, such as ours, take a number of steps to reduce publication bias, as represented by the fact that 21 of the 34 (61.8%) included studies in our main analyses came from unpublished sources (13 Goldstein Award Submissions, 4 research reports, 2 doctoral dissertations, and 2 Master's theses). Wilson has argued that there is often little difference in methodological quality between published and unpublished studies, suggesting the importance of searching the “gray literature” (Wilson, 2009). For our review, there may also be a bias in unpublished studies that are nevertheless available for review, since 13 studies were identified through the Goldstein Award competition. As noted earlier, award submissions are inherently biased toward successful programs. This was evidenced by our moderator analyses, which showed effect sizes were significantly larger for award submissions versus the other publication types.

Here we focus on an overall comparison of the 13 studies published in scholarly journals versus the other 21 studies (20 for the RIRR approach) through use of moderator analysis. For Cohen's *D* model, the mean overall effect size for studies published in scholarly journals is 0.156 (*p* = .002; *Q* = 46.482, *df* = 12, *p *< .001) and for unpublished studies the average effect is 0.199 (*p* < .001; *Q* = 111.367, *df* = 20, *p* < .001). Moreover, the moderated effect size is 0.184 (*p* < .001) and the between models heterogeneity test was not statistically significant (*Q* = 0.468, *df* = 1, *p* = .494). Similar findings are seen in the RIRR model. The mean effect for the scholarly journal studies shows a relative decline of 29.7% (*p* = .005; *Q *= 63.518, *df* = 12, *p* < .001) while the unpublished studies show a relative decline of 35.1% (*p* < .001; *Q *= 141.113, *df* = 19, *p *< .001). The moderated effect size shows a relative decline of 33.8% and the between models differences test was again not statistically significant (*Q* = 0.145, *df* = 1, *p* = .703). The lack of significance for the between‐model tests, along with the similarity between the mean overall effect sizes, suggests that publication bias may not have major impact on the outcomes of this review.

To more formally assess publication bias we generated a funnel plot to examine for possible selection bias in our results. This is shown in Figure 8a,b. A visual inspection indicates some asymmetry with more studies with a large effect and a large standard error to the right of the mean than the left of the mean. We used the trim‐and‐fill procedure developed by Duval and Tweedie (2000) to examine how our estimates would change in the absence of this asymmetry. The trim‐and‐fill procedure determined that nine studies should be added to create symmetry.

Figure 8Funnel plot to assess for publication bias. (a) Cohen's *D*. Empty circles are the studies included in our analyses, while the filled in circles indicate nine imputed studies for the trim‐and‐fill analysis. These additional studies lowered changed the mean effect size from 0.183 (95% CI = 0.0124–0.241) to 0.106 (95% CI = 0.043‐0.170). (b) Log RIRR. Empty circles are the studies included in our analyses, while the filled in circles indicate 11 imputed studies for the trim‐and‐fill analysis. These additional studies lowered the mean effect size from 0.291 (95% CI = 0.202–0.379) to 0.132 (95% CI = 0.040–0.223). In terms of relative risk reduced in treatment versus control groups this represents a decrease from the observed effect from 33.8% in the observed data to 14.1%. CI, confidence interval

**Figure:**
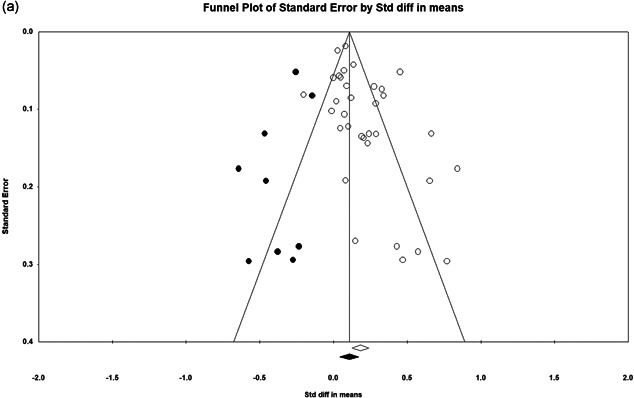

**Figure:**
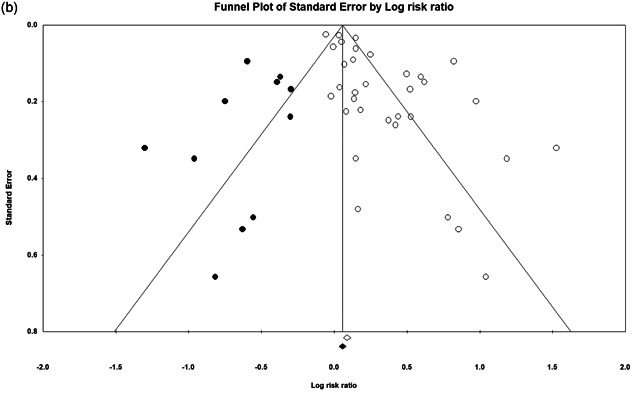

For Cohen's *D* model, these additional nine imputed studies slightly reduced the mean effect size from 0.183 (95% CI = 0.0124–0.241) to 0.106 (95% CI = 0.043–0.170). For the RIRR approach, 11 studies were imputed and this reduced the overall effect from 0.291 (95% CI = 0.202–0.379) to 0.132 (95% CI = 0.040–0.223). Put into relative risk reduction terms, the imputed studies decreased the overall relative reduction from 33.8% to 14.1%.

Along with the moderator analysis above, this suggests that while there is some potential for publication bias in our sample, especially given the nature of including police award submissions, it does not alter our overall conclusion of POP having an overall significant, meaningful impact on crime and disorder.

## 6 DISCUSSION

### 6.1 Summary of main results

The results of this updated review provide strong evidence that POP is effective in reducing crime. Across a large array of analyses, we find statistically significant impacts of POP. Overall we find that there was a 33.8% reduction in crime/disorder in the POP treatment areas/groups relative to the controls. At the same time, the effect sizes we observe are strongly heterogeneous and the overall effect is likely overstated as smaller effects were found when looking only at the randomized experimental evaluations and after accounting for publication bias. Nonetheless, the findings of those models still show that POP is associated with meaningful and statistically significant declines in crime/disorder in treatment groups relative to controls. Such heterogeneity across models is very common in meta‐analyses in criminology (Lösel, 2018), but points to the importance of going beyond what works in POP to what are the most effective strategies for specific problems.

Overall, our findings show that police following the tenets of the SARA model to identify specific problems, conduct analyses to examine underlying causes, and develop and deliver tailor‐made responses is an evidence‐based approach to crime prevention. This is especially true for property crime and disorder offenses based on our results. POP is also an approach that fits well with hot spots policing, another tactic that has been found effective in reducing crime through Campbell reviews (Braga, Turchan, et al., 2019). Some of our studies overlap with those in that review as they involve applying problem‐solving tactics at small hot spots of crime/disorder, and the authors of the hot spots review note that their strongest effects were associated with programs that involved problem‐solving efforts rather than just increased police presence/activity. The positive findings of both reviews suggest that combining the two approaches is likely a fruitful endeavor for crime‐control efforts.

As a number studies involved place‐based POP programs, it was important to also examine the potential for spatial displacement and diffusion of crime control benefits. Eight of our 26 place‐based studies reported data that allowed us to calculate effect sizes for displacement/diffusion. There is no evidence of spatial displacement of crime/disorder in these studies. Indeed, there is evidence of a small diffusion effect when looking at the mean effect across outcomes. This finding, along with a similar finding of a small diffusion effect in the hot spots policing review (Braga, Turchan, et al., 2019), suggests that place‐based policing efforts do not simply cause crime to “move around the corner” (see Weisburd et al., 2006).

There was some evidence that research design moderated the magnitude of the impact of POP on crime/disorder. The effect size for quasi‐experiments was somewhat larger than that for randomized experiments. However, the mean overall effect is significant for both models (as well as the moderated effect size). The same was true when examining the award submissions, which are inherently biased toward success and larger effects, as those had larger effects than the other types of studies. Nonetheless the overall effect for the non‐award submission studies was statistically significant. Adding to this the fact that nearly all of the studies were weighted to the prevention side of the distribution across analyses, we have additional confidence in our overall conclusion.

Finally, as noted above, if underlying causes at problem places are successfully addressed, the crime‐reduction benefits at targeted locations may be longer lasting and more meaningful in terms of overall crimes prevented compared with a similar effect size for a temporary police crackdown on an area or an intervention delivered to individual offenders. In plain language, there may be more “bang for our buck” when lasting changes are made at places, versus crackdowns that see deterrent effects erode when the program ends or person‐based programs that have to be continually delivered to different individuals over time. Unfortunately, existing studies do not examine crime prevention benefits in the long run, and are generally limited to follow‐up within a year or less (see also Weisburd & Majmundar, 2018 for a similar critique of short‐term follow‐up periods for POP studies). Future evaluations should include longer follow‐up periods so that later updates to this review can quantitatively assess the potential lasting impacts of POP.

### 6.2 Overall completeness and applicability of evidence

The findings of this review have widespread applicability to policing and crime prevention given the consistency of the conclusions drawn across all of our models. This review also represents a large increase in the available body of evidence in the time since the original review, which included only 10 studies (4 randomized experiments and 6 quasi‐experiments) and 16 outcomes. The current review includes an additional 24 studies (5 randomized experiments and 19 quasi‐experiments) published through December 2018. Our overall model now provides a meta‐analysis on the impact of POP on a total of 70 crime and disorder outcomes across 34 studies. The inclusion of these additional studies reaffirms the conclusion of the original review about POP's effectiveness in reducing crime and disorder with support from a much larger number of tests. While most studies (82.4%) were conducted in the United States, the fact that 5 were conducted in the United Kingdom and 1 in Canada offer initial support that POP is applicable in different contexts. However, this is still limited and caution may be needed when trying to generalize these findings to contexts outside of North America and the United Kingdom. We also were unable to perform a meaningful meta‐analysis on noncrime outcomes. With the current data we could only provide a preliminary, narrative summary of study findings due to the small number of studies that report on the same noncrime outcomes captured through similar measures. The same was true for cost‐benefit analysis.

### 6.3 Quality of evidence

The overall quality of evidence has improved from the original review with the inclusion of 5 new randomized experiments and 19 quasi‐experiments. However, as we discussed above and assessed with our moderator analyses, POP remains an area that needs more rigorous evaluations. The majority of studies are still quasi‐experiments, and several are weaker designs with unmatched control groups, comparisons of a target area to the rest of a jurisdiction and so forth. While we have confidence in our conclusions as the main finding of a significant effect for POP holds when looking only at the most rigorous studies, caution is needed in individually interpreting the larger effects from the weaker studies—especially the award submissions which are inherently biased toward positive outcomes.

We note that while more randomized experiments, and quasi‐experiments with matched control groups/areas, would improve the quality of evidence on POP, the existing body of evidence is largely a function of the nature of the POP model. The approach calls for identifying specific problems to be researched and addressed and it is often not going to be possible to create a well‐matched comparison area in a study jurisdiction (Eck, 2006a, 2006b). Similarly, the POP model calls on police to identify, analyze and respond to problems, and to then assess whether their efforts are successful. In this sense, the award submissions are evidence of the SARA model in action and are important to include in reviews such as this.

Moreover, the fact that 11 additional award submissions were eligible for this updated review (only two were included in the meta‐analysis in the original review) is indicative of an increase in rigor as more agencies are using control groups, even if unmatched, instead of simple pre–post case study designs. As such, these studies are important evidence and simply require caution in interpreting individual effect sizes and acknowledgment that there is a “file drawer” problem here as agencies are not going to submit (or even write up research reports) for programs that failed. On that front, we retain confidence in our findings as our analyses above suggest that publication bias was not a major concern in our study.

### 6.4 Limitations and potential biases in the review process

In general, there are no specific limitations or biases in the review process used in this study beyond those inherent to the systematic review process. Namely, all reviews will have a “file drawer” problem to some extent, and the threat may be slightly higher for POP than other approaches due to the assessment step of the SARA model asking police to test whether their efforts were effective. As discussed above, these findings are unlikely to be written up (much less submitted for award consideration) when efforts fail. Moreover, the use of the Global Policing Database is a huge asset to the current review. The traditional and gray literature sources searched to compile that database are far more exhaustive than those used in individual reviews (see Appendices B and C), including the original version of this review. Lastly, there were 15 (13 of which were award submissions) potentially eligible studies of POP that we could not include as we could not calculate standardized effect sizes due to insufficient or inadequate information being presented (see Appendix F). As noted above, we do not feel the absence of these studies biased our conclusions as the 13 award submissions discussed positive impacts of POP and the other two studies reported null effects, but no backfire effects.

### 6.5 Agreements and disagreements with other studies or reviews

The findings of this review reaffirm those of the earlier review (Weisburd et al., 2008, 2010) and existing narrative reviews that conclude that current evidence suggests that POP is one of the most promising police approaches to preventing crime (Skogan & Frydl, 2004; Weisburd and Majmundar, 2018). Moreover, the limited evidence on displacement and diffusion confirms the findings of other studies of place‐based crime prevention efforts by showing evidence of diffusion of benefits (Bowers et al., 2011; Braga, Turchan, et al., 2019; Weisburd & Majmundar, 2018; Weisburd et al., 2006). This finding is contrary to arguments made by others that displacement is the likely outcome of place‐based interventions (Blattman, Green, Ortega, & Tobón, 2017; Reppetto, 1976).

## 7 AUTHORS’ CONCLUSIONS

### 7.1 Implications for practice and policy

Evidence from this review suggests that POP is an effective crime prevention approach. While there is a great deal of heterogeneity in effect sizes across studies and outcomes, 31 out of 34 studies (91.2%) have effect sizes in favor of a treatment effect. Moreover, the overall effect is positive and significant in all of our mean effect size models. In short, the findings suggest that POP is a promising approach to reducing a variety of types of crime and disorder in a variety of contexts (the 34 included studies included 29 unique jurisdictions in 3 countries).

Our findings suggest that it is important for police departments to be fully behind POP efforts if they are to succeed. For instance, the lone backfire effect in the study (Stokes et al., 1996) involved a program that was barely implemented as two‐thirds of students at the target school were unaware of the existence of the school safety corridor and the corridor was poorly staffed in the after school hours due to the timing of police shift changes and limited police resources. Similarly, a null effect was reported in the study by Stone (1993), who noted that the police department did not seem entirely interested in implementing POP and that study officers did not view problem solving as “real” police work. This and other factors led to the program being chronically understaffed.

However, our results also highlight the fact that POP efforts can be successful even if the SARA approach is not delivered in its ideal version. This is important as studies have noted that it is difficult to fully implement the ideal model (Braga, Turchan, et al., 2019; Weisburd & Braga, 2006; Cordner & Biebel, 2005; Eck, 2006b; Maguire et al., 2015). Our findings show that even though the SARA model is often loosely followed, with the problem analysis often being shallow rather than in‐depth (see Table 2b), the approach is still found to lead to crime reductions when compared with control areas that received standard police services. This adds support to arguments that even “shallow” problem‐solving efforts can be lead to significant reductions in crime (Braga & Weisburd, 2010).

POP can also be fruitfully combined with other police tactics that have been found effective in recent Campbell reviews. In particularly, the POP approach has been shown to be effective when combined with hot spots policing. Braga, Weisburd, et al. (2019) found larger effect sizes for POP approaches at hot spots than approaches which simply increased police presence/activity at target areas. Elements of POP often also underlie the focused deterrence approach, which has been found effective and could perhaps be enhanced with more in‐depth problem solving in future programs rather than largely replicating the existing “pulling levers” model (Braga, Weisburd, et al., 2019).

There is also potential for combining POP with the popular Compstat model. Indeed, “innovative problem solving” is one of the key elements in the ideal form of Compstat. While evaluations of Compstat in the United States suggest that the emphasis on holding commanders accountable through Compstat meetings has limited innovative problem solving in the field (Weisburd, Willis, Mastrofski, & Greenspan, 2019), a recent Israeli program suggests that innovative problem solving can be encouraged in a Compstat‐like reform (see Weisburd et al., Unpublished manuscript). In that study, evidence‐based policing practices were strongly encouraged in the context of a national POP reform program, and the message of the program was communicated more directly to the rank and file. Robust prevention benefits were identified in moderate and large size police agencies in quasi‐experimental analyses of property crimes.

### 7.2 Implications for research

Our study identified 70 tests of POP in 34 included studies. Our meta‐analyses suggest that overall there is a significant effect of the approach in reducing crime and disorder. Our moderator analyses showed that effects are larger for the quasi‐experiments compared with the randomized experiments. Nonetheless, this does highlight the need for more rigorous evaluations of POP in order for a future update of this review to provide a more robust estimate of overall effect size.

This is not in any way meant to discourage quasi‐experimental evaluations of POP, or even pre–post case studies. As discussed earlier, the nature of the POP model means there may sometimes only be a single area with the problem being treated, and even if there are multiple locations it will often be difficult, and sometimes impossible, to identify suitable control areas—much less to identify enough sites to create a randomized experiment with sufficient statistical power. As such, knowledge from less rigorous research remains important. That said, given the similarity of our current findings to those of our original review, we view it as unlikely that future updates will shed new light on our knowledge of POP's effectiveness in the absence of more rigorous evaluations.

In our view, the question at this point is not whether POP works. The vast majority of the studies we reviewed show prevention benefits. This conclusion is also supported by past narrative reviews (Skogan & Frydl, 2004; Weisburd & Majmundar, 2018) and the summary of simple pre–post case studies provided in our original review (Weisburd et al., 2008, 2010). The key remaining questions surround what characteristics are associated with larger impacts of POP on crime. To truly assess this, future evaluations need to not only be more rigorous, but also must capture and report more data about the problems targeted, the level of problem analysis applied, the specific responses actually delivered and report outcomes more often by crime type than aggregate categories. The current sample of studies have a combination of lack of detailed information on many of these factors and tremendous heterogeneity in what is reported, which makes it difficult to draw strong conclusions about what makes POP most effective.

But irrespective of reporting practices, to build an evidence base that will be useful for practitioners in the field it is important for there to be a robust evidence base that is related to specific problems and specific interventions. This is a limitation more generally in the crime and justice field, but is particularly important to address when we have strong evidence of crime prevention effectiveness of a strategy, as we do here. Practitioners want to know what works in what situations, and which practices are most cost‐effective. Building such an evidence base would take a major federal or foundation effort to advance the practice of POP, and literally hundreds of studies testing practices in regard to specific types of problems. In turn, we have limited cost‐benefit analysis data from the studies we reviewed. The studies that did examine cost savings generally used limited data to estimate both costs and benefits and rarely did any systematic analysis. For practice it is not simply whether something works, it is equally important to provide a sense of what cost for what benefit. Answering this question should be a major focus of future studies.

The authors of this review plan to do a deeper dive and coding of the eligible studies to see if more light can be shed on these matters in a follow‐up publication. The prospects of succeeding in this effort with existing data are unclear, and was beyond the scope and timeline for this funded review.

On this front, it is important for more future studies to evaluate the impact of POP on outcomes beyond the standard crime and disorder outcomes examined in the studies included in this review. These studies tested impacts on aggregate crime/disorder, violence, property crime, disorder, drug sales/use and related outcomes such as probation/parole success or failure. POP was proposed as a flexible model that can be applied to a wide array of problems and our understanding of the model's potential would be enhanced through studies that assess its impact on issues such as cyber‐crime, human trafficking and other issues increasingly of interest to criminologists and criminal justice practitioners. Additionally, more future studies should be designed to examine its applicability in reducing resident fear of crime, improving citizens’ opinions of the police, and bolstering collective efficacy. Too few existing evaluations report on such outcomes to allow for a meaningful meta‐analysis, but our narrative review of the existing evidence suggests mixed and inconsistent findings across studies. This suggests the need for further research, particularly on POP interventions that include close partnership with and involvement of the community, which might be expected to have the greatest impacts on these perception‐based outcomes.

Lastly, this updated review also added the approach of performing meta‐analyses using log RIRRs as the effect sizes. This was done based upon in‐progress work by David Wilson which argues both that Cohen's *D* fails to produce effect sizes that are comparable across studies when based on place‐based count data and that the conversion of RIRR to Cohen's *D* is problematic. We still reported Cohen's *D*, including a majority (27 out of 34) of effects that were converted from RIRR, as we wanted to be consistent with the approach used in our original review (Weisburd et al., 2008, 2010) and other recent Campbell Reviews such as the updated hot spots policing review (Braga, Turchan, et al., 2019). This also allowed us to compare the two approaches.

Our results show that while similar conclusions would be reached about POP's effectiveness using either approach, it does appear based on our comparisons and examples discussed above in Section 5.5 that the Cohen's *D* approach may understate the impact of place‐based interventions, and that the RIRR approach appears to generate effect sizes that are more in line with actual changes reported in the studies themselves. Moreover, being able to convert the log RIRR to relative change in the treatment group versus the control group makes effect sizes more intuitive for researchers and practitioners alike. For instance, for our Cohen's *D* model our mean overall effect of 0.183 is not going to immediately tell a police leader much about the effectiveness of POP. However, using the RIRR approach allows us to more simply state that the relative reduction in the POP groups versus the controls was 33.8%. This is much more easily interpretable to practitioners, which is an important aim of Campbell Systematic Reviews.

Given that the RIRR approach is both more informative for practitioners and, based upon David Wilson's (in progress) work, more appropriate for place‐based studies (while also avoiding the problematic conversion of RIRR to Cohen's *D*) we encourage future meta‐analyses of place‐based interventions to adopt this method.

## CONFLICT OF INTEREST

Weisburd is an author on four of the included studies and has been author or coauthor of several studies that have found POP and other proactive policing approaches effective, coauthored a book on POP with Anthony Braga and served on National Academy of Science panels which concluded that POP is a promising approach for crime prevention. Telep has coauthored a problem‐solving guide for the POP Center and has coauthored narrative reviews of policing strategies and helped design the Evidence‐Based Policing Matrix which suggests POP and similar approaches are effective based on existing evidence. Weisburd and Telep do not have any ideological bias toward the effectiveness of POP. Nonetheless, the inclusion of additional authors without prior work in this area reduces unconscious biases. Hinkle and Petersen have not conducted evaluation research or published on the effectiveness of POP outside of this Campbell Review (including the original version for Hinkle).

## ROLES AND RESPONSIBILITES

J. C. H., D. W., and C. W. T. designed the original systematic review following established Campbell Collaboration conventions and procedures, with assistance from John Eck and Phyllis Shultz. J. C. H., D. W., and C. W. T., designed the updated review with assistance from the GPD team of Elizabeth Eggins, Lorraine Mazerolle, Angela Higginson. This team also performed the search of the GPD and sent results to J. C. H. Forward searches of seminal POP studies and manual inspection of recent volumes of leading journals and the submissions to Goldstein and Tilley awards were performed by K. P. Title and abstract screening for eligible studies was performed by K. P., with any studies that were not obviously eligible or ineligible reviewed and ruled upon via a vote by J. C. H., D. W., and C. W. T. Coding of each study was performed by K. P. and one other graduate student assistant (either Julia Durska or Taryn Zastrow). All discrepancies between the two coders were reviewed, discussed and resolved via vote by J. C. H., D. W., and C. W. T. All effect sizes were calculated by J. C. H. (with some help from David Wilson and Anthony Braga). All analyses were conducted by J. C. H. The literature review and methodology sections of the report were written by J. C., H. D. W., and C. W. T. Summary information about studies (narrative reviews, review of reported implementation problems, review of reported bias and the associated tables for these sections) was drafted by K. P. The narrative review of impacts on noncrime outcomes was written by C. W. T. The results and discussion/conclusion sections were written in close collaboration between J. C. H., D. W. and C. W. T. All authors read, edited and commented on all sections of the report.

- Content: J. C. H., D. W., C. W. T., and K. P.
- Systematic review methods: J. C. H., D. W., C. W. T.
- Statistical analysis: J. C. H.
- Information retrieval: Elizabeth Eggins, Lorraine Mazerolle, Angela Higginson, J. C. H., and K. P.

## SOURCES OF SUPPORT

This updated review was supported by the Campbell Collaboration through funding provided by Problem Solving and Demand Reduction Programme, hosted by the South Yorkshire Police. The original review was supported by Award 2007‐IJ‐CX‐0045, awarded by the National Institute of Justice, Office of Justice Programs, U.S. Department of Justice and the Nordic Campbell Centre.

## PLANS FOR UPDATING THE REVIEW

Joshua Hinkle will coordinate the next update of this review with support from David Weisburd, Cody Telep and Kevin Petersen. We plan to update this review every 5 years, in accordance with Campbell Collaboration Guidelines. As the search strategy of this review depends upon the GPD, the plan is to carry out an update when five additional full years of studies (work through December 2023) have been fully indexed into the database.

## Figures and Tables

**Figure C1:**
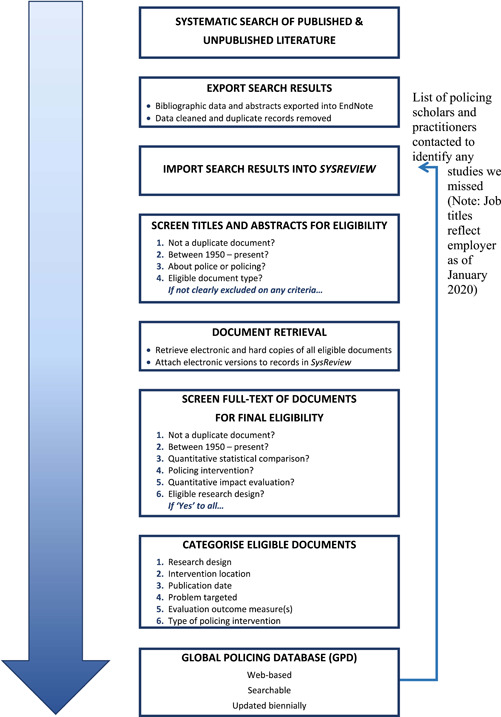
GPD systematic compilation process

## Footnotes

^1^
We note that SARA is not the only model for carrying out POP. Other models, most being refinements/elaborations to SARA, have been introduced over the years including PROCTOR, The 5Is, the 9‐State Model and ID Partners. Sidebottom and Tilley (2011) provide a review and study of these approaches and note that SARA remains the dominant model. It is important to note that our review did not require explicit mention of the SARA model and programs using any of the models listed above or other similar approaches to carry out the basic tenets of POP were eligible for inclusion.

^2^
A series of systematic reviews of “hot spots policing” has been conducted by Anthony Braga and colleagues (2001, 2007, 2014, 2019). Hot spots policing focuses on small geographic areas and concentrations of crime. Hot spots policing per se does not demand detailed analysis of the problem identified and often relies on a law enforcement response. POP can focus on small geographic areas (hot spots); however, further analysis is undertaken to determine the creation of the hot spot and responses are tailored to the needs of each hot spot. Further, POP also examines non‐geographic concentrations of crime—repeat offenders, repeat victims, hot products, and so forth. In short, while POP at hot spots can be considered a type of POP, many hot spots policing programs do not use the more systematic methods associated with POP.

^3^
The Tilley Award went on hiatus after 2010 and was recently resumed by the Problem Solving and Demand Reduction Programme hosted by the South Yorkshire Police. The most recent winner was announced in March 2019.

^4^
Seventy‐three percent of departments serving 250,000–499,999 citizens reported encouraging problem solving among officers (Reaves, 2015). The same was true for 71% of departments serving 500,000–999,999 and 57% of those serving a million or more residents.

^5^
However, we note that many of the studies reviewed in these areas employed relatively weak designs (Clarke, 1997; Eck, 2002; Weisburd, 1997).

^6^
As with our original review (Weisburd et al., 2008, 2010) we opted not to include pulling levers/focused deterrence approaches. The original Boston Gun Project (Braga, Kennedy, Waring, & Piehl, 2001), which can be viewed as a POP approach that would meet our SARA‐based intervention criteria, only made comparisons to other cities as it was a citywide program. We deemed this did not meet our methodological criteria. Most other pulling‐lever approaches have largely replicated all or parts of the original model rather than going through the SARA process to identify a specific problem and develop a tailor‐made response. Thus, they do not meet our intervention criteria. Additionally, there is a recently updated Campbell systematic review of these programs, which summarizes their impacts on crime (Braga, Turchan, et al., 2019).

^7^
The seminal pieces that were searched for are: Braga, Weisburd, et al. (1999), Eck and Spelman (1987), Goldstein (1979, 1990), Spelman and Eck (1987), Weisburd et al. (2008, 2010).

^8^
These journals included: *Cambridge Journal of Evidence‐Based Policing, Criminology, Criminology & Public Policy, Justice Quarterly, Journal of Research in Crime and Delinquency, Journal of Criminal Justice, Police Quarterly, Policing, Police Practice and Research, British Journal of Criminology, Journal of Quantitative Criminology, Crime & Delinquency, Journal of Criminal Law and Criminology, Policing and Society: An International Journal of Research and Policy*.

^9^
The POP Center is included in the GPD's gray literature searches, but given the centrality of the center to the current review topic we opted to double check this source for eligible studies.

^10^
Note we used a slightly different adjustment for over‐dispersion in our original review (Weisburd et al., 2008, 2010). We opted to use the current approach for the update to be consistent with more recent Campbell reviews, including Braga and colleagues (2019) recently updated review of hot spots policing (which includes a handful of overlapping studies/outcomes in cases of POP approaches evaluated at hot spots). Additionally, David Wilson (personal communication) has recently developed a new approach for a more robust adjustment for over‐dispersion when place‐based studies report crime counts for multiple treatment and control areas (or multiple time periods within each group). Unfortunately, we were unable to take advantage of this with our data as too few studies reported on more than one treatment or control area (or only provided aggregated counts by area). For those that did provide disaggregated counts, it was often only two or three areas per group, which is not a sufficient sample for a reasonable adjustment for over‐dispersion in our view.

^11^
One study (Groff et al., 2015) provided the log IRR and its standard error. For three studies, the effect sizes are risk ratios rather than RIRRs. Two studies reported on probation/parole success and failures in a treatment and control group (Knoxville Police Department, 2002; Thomas, 1998) and one reported on students being attacked or not in a treatment and control school (Stokes et al., 1996). While these are calculated slightly differently, they are comparable to RIRR and interpreted in the same manner. The risk ratios were calculated as follows. Referring to the RIRR two‐by‐two grid above, here *a* and *b* are successes (or not being attacked) in the treatment and control group, respectively. Similarly, *c* and *d* are the failures (or being attacked) in the two groups. In the traditional notation for risk ratios (see Wilson, in progress), the treatment group successes are indicated by *T* and the control group successes are labeled *C*, while *N*_T_ and *N*_C_ are the sample sizes (successes plus failures) in the treatment and control groups, respectively. RR is then calculated as (*T* × *N*_C_)/*(C* ×* N*_T_) and its log is the effect size in our models. The variance of log RR is calculated as (*N*_T_ −* T*)/*T* ×* N*_T_) + (*N*_C_ −* C*)/*C* ×* N*_C_) and its square root is the standard error for the log RR.

^12^
This 2019 publication is included as the “online first” version came out in 2018 and was included in our search results.

^13^
Our findings here differ somewhat from those of the National Academy of Sciences Committee on Proactive Policing. Weisburd and Majmundar (2018, p. 209) concluded “studies show consistent small‐to‐moderate, positive impacts of problem‐solving interventions on short‐term community satisfaction with the police.” This difference is likely because of our focus on these outcomes only among our eligible studies, which all had a primary crime‐control emphasis. Thus, we caution that our findings here do not necessarily reflect all problem‐solving projects, and in particular do not include studies that did not meet our other eligibility criteria.

## APPENDIX A NARRATIVE SUMMARY OF INCLUDED STUDIES

### Baker and Wolfer (2003)

Baker and Wolfer (2003) describe a problem‐oriented policing (POP) intervention in a small Pennsylvania town aimed at targeting vandalism and substance use in a local park. During the scanning and analysis process, officers noted that the park was full of litter and had overgrown brush, allowing offenders to hide from police. Using crime prevention surveys and crime mapping, they determined that the problem was isolated in the small area in and around the park. To respond, officers target hardened by removing overgrown shrubs. They used other methods of situational crime prevention by installing cameras, repairing fences, improving lighting, locking the park at night, limiting access, and posting rules and regulations. In addition, the police used proactive patrol and increased enforcement of the curfew law to target juvenile offenders. Officers worked with residents to establish a Neighborhood Watch to coordinate cooperation between the police and area residents. To assess the project, researchers used a quasi‐experimental design with a comparison group. Volunteers administered 29‐question surveys both before and after the project to random samples of residents in the immediate area of the park and a comparison group of residents who lived in the same town, but not adjacent to the park. Pre‐assessment survey results indicated that residents in the target group reported witnessing significantly more vandalism and public drinking/disorder than residents in the comparison group. However, target group residents reported a greater reduction in these behaviors by the post‐assessment period, and the difference between the groups was no longer statistically significant. Target group residents also reported a greater reduction in overall victimization and larger increases in feelings of safety. Some evidence of displacement and dispersion was reported, but no tests of statistical significance were presented.

### Bichler et al. (2013); Chula Vista Police Department (2009)

The City of Chula Vista's tourist economy had been suffering as a result of high crime and disorder at budget motels. Due to the economic ramifications, business organizations began to reach out to the police and local government in hopes of partnering to address the problem. Initial efforts to address motel crime through increased enforcement were deemed unsuccessful, leading to an alternative problem‐solving approach. The police began analyzing trends in calls for service reports and call narratives, surveying motel users, interviewing managers, and conducting environmental assessments. Analysis indicated that management policies and practices were facilitating crime and that place managers may be an effective target for the response. The proceeding intervention unfolded in three separate stages. In the outreach stage, the project staff began producing calls for service report cards for each motel and distributing them individually to motel operators. These reports summarized the level of crime at each respective motel in an effort to increase awareness on the part of motel operators. The outreach stage also included seminars held for motel managers to provide technical assistance as well as education on safety improvements. Following outreach, project staff began the code enforcement stage. With cooperation from local government, motel facilities were inspected, and violations were enforced. Additionally, staff began distributing motel calls for service rankings to all managers in attempt to shame them into making changes. When the problem persisted, a permit ordinance stage was initiated. In this stage an annual permit to operate, based on calls for service rates, was instituted via a multi‐agency collaborative effort. Motels with call levels under a certain threshold were granted license, while those above the threshold were required to enter into a memorandum of understanding that required appropriate action to address crime problems. To assess the impact of the intervention, Bichler et al. (2013) employed a nonequivalent control group quasi‐experimental design. To search for valid comparison units, researchers identified motels within the same region that experienced similar levels of crime and environmental characteristics (such as proximity to major freeways). This process led to the selection of 10 motels from a nearby city. Calls for service rates at these comparison motels were then compared with the calls for service rates at 15 intervention motels during both pre‐ and postintervention time periods. Additionally, researchers selected nine displacement/diffusion motels within proximal distance to the target motels. Raw count comparisons revealed a greater overall reduction in calls for service at intervention motels compared with control motels, and *χ* ^2^ analysis indicated that the overall change was statistically significant. Further, there was no evidence of spatial displacement in the displacement/diffusion motels. The evaluation also examined police and city costs, as measured by personnel hours devoted to the problem. Results indicated notable reductions in dedicated hours across all categories in the postintervention period, suggesting that the program was cost‐effective.

### Bond and Hajjar (2013)

As part of a Smart Policing Initiative (SPI), the Lowell (MA) Police Department (LPD) partnered with local researchers to implement a problem‐solving intervention targeting property crime hot spots. Using incident data derived from the LPD's crime analysis unit, the SPI team discussed the location of potential hot spots and ultimately selected four hot spots in each of the city's three police sectors. Comparison hot spots were then chosen based on similarities to target areas in both crime and other characteristics, resulting in a nonequivalent control group quasi‐experimental design with four matched pairs. As part of the analysis process, captains from each sector completed biweekly surveys documenting the strategies being used in each hot spot. At these biweekly meetings staff reviewed responses and analyzed outcome data, resulting in an iterative problem‐solving process. While specific problem dynamics varied by hot spot, all areas focused on various forms of property crime (i.e., larceny, theft from a motor‐vehicle, burglary, shoplifting) as well as drug violations and prostitution. Response activities generally included directed patrol, traffic enforcement, increased visibility, community meetings, and in some cases partnership with inspectional and neighborhood services. To assess the effectiveness of the intervention, property crime incident counts for each hot spot were aggregated at the sector level. Raw incident counts were then compared before and after the intervention for both treatment and control groups. Simple comparison of percentages indicated that treatment hot spots witnessed a greater decline in aggregate property crime within each sector than comparison hot spots, with reductions ranging from 16% to 19% per sector. Additionally, anecdotal evidence suggested that the problem‐solving initiative may have improved communication and organizational functioning within the LPD.

### Boston Police Department (2008)

The Boston Police Department (2008) noticed increasing public concerns at neighborhood and community meetings over home security and residential burglary rates. An analysis of official data and incident maps suggested an increasing concentration of residential burglary incidents in District D‐14. These data also revealed several consistently problematic days within the week. Police began conducting environmental assessments of addresses experiencing three or more residential burglaries within the past year, revealing numerous security weaknesses. Reviews of prior call handling also revealed a lack of attention to detail in these investigations, and conversations with tenants and property management highlighted security risks in current management practices. Based on these data sources, the police developed a three‐pronged response plan. First, the department increased resources devoted to burglary investigations by assigning dedicated detectives to incidents and partnering with the city's housing division to make security changes to residential buildings. Second, the department held meetings with building owners and residents to educate them about target hardening techniques and general safety measures. Finally, police focused on hot spot enforcement by increasing presence and surveillance near areas that experienced the most burglary incidents. This included an undercover operation in collegiate residential areas during the spring break period. The intervention was assessed using a nonequivalent control group quasi‐experimental design. The evaluation compared residential burglary incident counts for the year preceding the intervention to the year after implementation. Incident counts were compared between District D‐14 and the rest of the city. The assessment showed promising results, with the intervention district accounting for only 12.4% of the citywide residential burglaries in the postintervention year, as compared with 20.5% in the year preceding. The authors also noted that there was no evidence of displacement, based on measurement of burglary rates in nearby jurisdictions. However, no statistical tests of displacement were presented.

### Braga et al. (1999); Braga (1997)

Braga and colleagues (1999) document a POP project in Jersey City, NJ designed to address hot spots of violent crime. These hot spots were defined using computerized mapping and then officers worked to determine what problems existed at each hot spot. After initially choosing 28 pairs of violent crime places, the randomized experiment was narrowed to 12 pairs: 12 hot spots received POP and 12 received traditional patrol. Additionally, two‐block catchment areas were built around each of the 24 places to test for displacement effects. In the 12 treatment pairs, officers were required to complete an analysis report assessing the specific problems in the particular hot spot. They were encouraged to use official data and meetings with, or surveys of, community members. Although all the hot spots were chosen because of high rates of violence (typically street fights, drug market violence, and/or robbery), officers also identified widespread disorder problems that included public drinking and loitering. Officers designed a response to specifically target the problems they uncovered in the analysis stage. Thus, the exact response varied by hot spot, but the responses all included some aspect of aggressive order maintenance and most included efforts to make physical improvements to the area (e.g., removing trash, improving lighting) and drug enforcement. To assess the project, the researchers used calls for service data, incident data, and pre‐ and postobservations of physical and social disorder. Crime incident and calls for service outcomes were assessed using a 6‐month preintervention and 6‐month postintervention time period. Generalized linear models indicated a main effect of treatment on both aggregate criminal incidents and calls for service, as treatment places were associated with significant reductions in both measures compared with control locations. Further analysis revealed significant reductions in street fighting, property, and narcotics calls for service, as well as robbery and property incidents at treatment places relative to control. Pre‐ and postobservations of social and physical disorder indicated that, in both categories, disorder was reduced at 10 of 11 treatment locations (one location was dropped from analysis), and these reductions also reached statistical significance. Displacement/diffusion results did reveal significant displacement of property crime incidents to catchment areas. However, no other crime types showed evidence of displacement, and several outcomes may have shown evidence of diffusion of benefits.

### Braga and Bond (2008, 2009)

Braga and Bond (2008) evaluated a research‐practitioner partnership with the Lowell, MA Police Department (LPD) to implement a problem‐solving initiative targeting hot spots of overall crime and disorder. As part of the scanning phase, crime and disorder‐related calls for service were mapped for the prior year, and temporal analyses were conducted to identify areas with persistently high call levels. Hot spot boundaries were then identified with input from LPD officers and consideration of place characteristics, resulting in 34 distinct hot spots. For each hot spot, a two‐block catchment area was also constructed to measure potential displacement and diffusion of benefits. Following a randomized experimental design, the 34 hot spots were matched into 17 pairs (based on the qualitative characteristics of the areas) and then randomly assigned to treatment or control from within each pair. Treatment hot spots were assigned to police captains who were then required to submit analysis reports detailing the causes of the area's crime problems and the proposed response measures. Analysis sources for treatment hot spots generally consisted of official data and conversations with community members. Throughout the intervention the LPD held monthly meetings with captains at which current strategies would be assessed and amended if appearing to be unsuccessful. Specific responses varied by hot spot, but all measures were broadly considered either situational strategies (cleaning vacant lots, nuisance abatement, improved lighting and video surveillance, code inspections, etc.), social service strategies (providing mental health resources to problem tenants, working with shelters for the homeless, providing recreational activities for youth, etc.), or order maintenance strategies (increased enforcement of disorder offenses, stop and frisk usage, targeting drug dealers, directed patrol, etc.). The control hot spots received standard policing. To assess the results of the experiment, calls for service rates for treatment and control hot spots were compared pre‐ and postintervention across a number of crime and disorder categories (assault, robbery, breaking and entering, larceny/theft, disorder/nuisance). In addition, pre–post observations of both physical and social disorder were conducted for treatment and control places. Analysis of calls for services outcomes was conducted using a 6‐month preintervention and 6‐month postintervention time period. Poisson and negative binomial regression models indicated significant reductions in assault, robbery, burglary, and disorder/nuisance calls for service in treatment hot spots relative to control hot spots. There were no significant changes in larceny/theft, however. These results also translated into a significant overall reduction in calls for service at treatment locations compared with control areas. Observations of physical and social disorder further revealed significant decreases in both categories for treatment locations compared with controls (physical disorder was reduced in 14 of 17 hot spots while social disorder was reduced in 13 of 17). In addition, there was no statistically significant evidence of either displacement or diffusion of benefits. Researchers also conducted a mediation analysis including the three main program elements (misdemeanor arrests, situational strategies, and social service strategies) as mediators between treatment and calls for service. Results of this analysis indicated that situational strategies produced the most significant crime reduction effect.

### Cooley et al. (2019)

The Canton (OH) Police Department (CPD) initiated a POP program targeting disorderly conditions in the Homestead neighborhood. Informal surveys with neighborhood residents indicated that there were concerns regarding quality of life issues. Officers were instructed to go into the neighborhood and take note of problems they would want to change if they lived in the area. Officers stayed in the neighborhood for extended periods of time. They learned that a frequent complaint from residents was that of landlords who facilitated crime by housing problem tenants. In response, the police created a database of problem landlords and confronted them directly. The CPD also attempted to increase trust in police by working with neighborhood groups and making appearances at a job training center for at‐risk youth. Other response activities included focused deterrence with repeat offenders, and partnership with parole to assist with offender reintegration. The evaluation conducted by Cooley et al. (2019) is an attempted replication of the Homestead intervention. The replication was conducted in another Canton, Ohio neighborhood that was also experiencing crime and disorder problems (McCormick neighborhood). The evaluation used a nonequivalent control group quasi‐experimental design. Counts of both violent offenses and quality of life offenses were compared between the treatment neighborhood and a comparison neighborhood (selected based on similar crime problems and neighborhood characteristics) across 12‐month pre‐ and postintervention time periods. Results indicated decreases in both violent and quality of life offenses for the treatment area, compared with minor increases in both categories for the control area. However, difference‐in‐difference analyses indicated that the magnitude of decline was not large enough in either category to reach the conventional level of statistical significance. Additionally, there were no significant differences found between the two groups in satisfaction with police or fear of crime.

### Dario (2016); Glendale Police Department (2016); White and Katz (2013)

In 2009 researchers from Arizona State University's Center for Violence Prevention and Community Safety partnered with the Glendale (AZ) Police Department on a SPI to target property crime using a problem‐oriented approach. As part of the SPI, officers were trained on the SARA model and were instructed to identify persistent problems. Officers selected convenience store crime due to its persistence and consequences for the department and community. During analysis, the team analyzed calls for service data for all 65 Glendale convenience stores and determined that the top 10 call generators were all Circle K stores. The stores were then mapped to determine if these trends were the result of location. Mapping revealed significant differences in call levels, even between Circle K stores and other convenience stores in the same area. Environmental surveys and CPTED assessments of Circle K stores were conducted, and the team noted numerous security issues in store design and management practice. To respond to these issues the SPI team developed a three‐pronged response that targeted the top six call generating Circle Ks. In response I, the SPI team met with Circle K leadership and presented the results of their CPTED assessments and recommendations for change. While some changes were implemented, this stage was met with limited success. The SPI team then convened other law‐enforcement agencies in the area, compared calls for service data, and determined that Circle K stores were problematic in other areas as well. This report was then provided to local media, resulting in coverage of the crime problem as a public shaming mechanism. In response II, the team developed prevention messages with help from local government that advertised the risks of convenience store theft. These messages were targeted at middle school and high school students. Finally, in response III the team engaged in suppression efforts involving increased surveillance and enforcement. To evaluate the intervention, White & Katz (2013) used a nonequivalent control group quasi‐experimental design. They compared call rates at the top six Circle K stores to call rates for the remaining nine Circle K stores in the city, as well as the top 13 other Glendale convenience stores. Statistical comparisons were conducted for the year prior to and year after the intervention. Using an analysis of variance model, White and Katz found a significant overall decrease of 42% in calls for service at the six intervention Circle K stores, with five of six stores experiencing individually significant call decreases. The nine comparison Circle K stores experienced an overall decrease of 31% in calls for service, however, this change was not statistically significant. Further, the non‐Circle K stores used for comparison experienced an overall increase of 0.5% from pre‐ to postintervention. Dario (2016) subsequently extended the pre‐ and postintervention periods by nearly a year and a half to assess the longitudinal effects of the program, while also including additional comparison stores. Difference‐in‐difference estimation and negative binomial regression further indicated a statistically significant effect of treatment. Intervention stores were found to have experienced 16.47% fewer calls for service relative to comparison stores, though the regression results were only significant for the full model (intervention stores vs. all nonintervention stores, rather than only nonintervention Circle K stores). Dario also conducted a displacement/diffusion analysis, finding no evidence of spatial displacement, but rather finding statistically significant evidence of a diffusion of benefits for five of six intervention stores. However, Dario noted mixed evidence of crime‐type displacement varying by individual store.

### Durham Constabulary (2017)

Police managers and researchers began to notice higher rates of dwelling house burglaries at certain locations and among certain victims. Tracking incident rates over the previous 5 years, police identified six neighborhoods that suffered from consistently high levels of burglary in which money and jewelry appeared to be targeted, and six neighborhoods where more general property was targeted. Three neighborhoods within each group of six were assigned to treatment and three were assigned to control, consistent with a nonequivalent control group quasi‐experimental design. Police analyzed the environmental structure of these neighborhoods and their consequences based on theories of environmental criminology. They also engaged with community members to gain additional perspective and former offenders to learn about methods used for offending. The police felt that the problem was the result of offenders repeatedly targeting areas that they were familiar with, requiring a change in both the physical structure and the behavior of victims. In response, the project staff distributed crime prevention supplies such as light timers, anti‐climb paint, security lighting, etc. to homes within intervention areas. Police also erected signage advertising neighborhood watch programs and held public meetings discussing the program to engage with residents. The assessment of the project involved comparison of burglary counts for the 5 years preceding the intervention to burglary counts for the year following implementation. Comparison was made between both treatment neighborhoods and their respective comparison areas. The program appeared to be effective based on a greater percentage decline at all target locations relative to comparison locations. It was further suggested that there was no evidence of displacement, though no statistical tests of displacement were presented. The authors also examined the economic feasibility of the program by comparing the cost associated with program implementation, the typical cost of a residential burglary to the victim and the justice system, and the overall reduction in incidents in treatment locations relative to comparison locations. Based on this analysis they conclude that the program was effective in generating cost‐benefit savings that outweighed the price of program implementation.

### Elliott (2007); Reno Police Department (2006)

Reno (NV) police officers assigned to work in the Downtown Tax District noticed abundant crime and disorderly conditions in the area's low‐budget motels. Business owners and tourists began complaining as well, leading to the initiation of a problem‐solving approach addressing motel crime in the area. Officers began conducting surveillance of the most problematic motels, noting any environmental security risks and code violations. The project officers then conducted interviews with suspects, motel residents, motel managers, and school representatives. Through these interviews and analyses of motel registration practices, officers learned that managers were improperly renting rooms and facilitating crime, while the children of tenants were frequently absent from school. Officers determined that changing management, physical security, and partnering with outside agencies to provide resources for families would all be necessary steps. The response phase began with initial warrant sweeps and door‐to‐door contacts at problem motels. Project staff then partnered with local agencies to explain and conduct code enforcement inspections, as well as present CPTED recommendations. Police also worked with motel managers to institute stricter registration procedures and worked with the County Attorney for special prosecution of motel crime offenders. Other response activities included communication with probation and parole to increase supervision of clients in the motel district, working with local community and social services to provide resources for families living in motels (i.e., food and clothing, computer access at local libraries, etc.), and educating motel managers on recognition of criminal behavior. The motels that received the intervention were all located in the Downtown Tax District, known as the Motel Interdiction Team (MIT) zone. Elliott (2007) assessed the effectiveness of the intervention by using a nonequivalent control group quasi‐experimental design. Calls for service and crime incident counts were compared before and after the intervention for the 35 motels in the MIT zone and the 30 motels outside the MIT zone (comparison motels). Evaluation of crime data was conducted using a 182‐day preintervention and 181‐day postintervention period, however, incident counts were subsequently dropped from the analysis due to low base rates. Results from the calls for service assessment indicated that there was a decrease in aggregate calls for service in the MIT zone compared with an increase in aggregate calls for service outside the MIT zone, though neither overall change was statistically significant based on *t* test results. This finding was largely driven by a significant increase in crimes against person calls outside the MIT zone, coupled with a nonsignificant decrease in crimes against person calls inside the MIT zone. Reno Police Department (2006) also reported that the decrease in calls for service within the MIT zone equated to an estimated savings of 1,750 police officer hours per year.

### Gill et al. (2018)

Researchers partnered with police in Seattle, Washington to address street segments of high‐frequency youth‐related crime/disorder. Police data was mapped and incidents involving youth (ages 12–25) as either suspects, arrestees, or victims were examined to identify street‐level concentrations of crime. The hottest street segments were then matched into two pairs based on incident rate and street‐level characteristics. Within the two matched pairs, street segments were randomly assigned to either treatment or control, resulting in the use of a randomized experimental research design. Small groups of officers were assigned to each intervention area to conduct a primarily non‐enforcement based problem‐solving initiative. During the analysis phase, the problem‐solving teams examined official data and CPTED assessments, as well as engaged in conversations with community and business stakeholders. Officers were encouraged to develop responses that focused on strengthening the infrastructure for youth, rather than relying on enforcement. Response activities differed between the two intervention sites, in the Westlake Park area officers partnered with community stakeholders and local government to promote collective efficacy and implement CPTED changes. Officers also worked with social services to provide shelter for a homeless individual that had been the genesis for other issues in the park. In the “Retail Street” intervention area officers initially began with targeted enforcement of adult drug dealers/users. Other responses included rerouted bus lines, improvements to street furniture, and increased cooperation with Metro Transit Police. The assessment was conducted using pre–post incident and calls for service data for both intervention areas, as well as their respective control locations. Poisson regression models indicated that the effectiveness of the program differed by intervention area. In Westlake Park, the intervention was associated with a nonsignificant increase in calls for service relative to the matched control area. Conversely, on Retail Street the intervention was associated with a statistically significant decrease in calls for service relative to the matched control. Findings were similar for crime incidents, with Westlake Park experiencing a respective increase, and Retail Street experiencing a respective decrease, relative to controls (though neither result was statistically significant by conventional standards). Gill et al. (2018) suggest that the differing effects may be related to the nature of the response activities. In Westlake Park, where enforcement responses were not used, program effects may be delayed, or measurement may not have adequately captured the changing dynamics within the community. On Retail Street, however, enforcement responses may have helped to contribute to short‐term crime reductions that are receptive to measurement.

### Groff et al. (2015); Ratcliffe et al. (2015)

The Philadelphia Policing Tactics experiment compared the effectiveness of POP, foot patrol, and offender‐focused policing in addressing hot spots of violent crime. Hot spots were identified by geocoding and mapping incident data from the prior year. Hot spot boundaries were then determined by District Captains based on their knowledge of the areas, and a total of 81 violent crime hot spots were selected. Police then divided these areas into groups of 27 based on which areas they felt would be best suited for each policing tactic, resulting in 27 potential problem‐solving hot spots. Due to the police department's desire to treat a certain number of total hot spots, randomization was conducted using a 3:1 ratio. Thus, within the group of POP hot spots, 20 locations were randomly assigned to treatment and 7 locations to control (standard policing). Problem‐solving was conducted by POP teams in each treatment location. Analyses and response activities varied by location, but District Captains were required to continually update and submit action plans and progress reports. Some responses included partnership with other agencies and enforcement measures such as focusing on known offenders and foot patrol. Additionally, a number of locations ultimately switched their focus away from violent crime, instead targeting property and drug‐related offenses. This randomized experiment was evaluated through a comparison of all violent crime and violent felony incident counts. Outcomes were measured pre‐ and postintervention for both treatment and control hot spots, as well as across policing tactics. Negative binomial models indicated nonsignificant changes in both violent felonies and all violent crime measures for POP treatment places relative to control. Furthermore, because the effects of the POP intervention were nonsignificant, displacement was not measured for these hot spots. Groff et al. (2015) suggest that the heterogeneity in targeted problems may have been one of the contributing factors to the lack of measurable violence reduction. Ratcliffe et al. (2015) also conducted a survey assessment of the intervention's effects on community perceptions. A total of 157 residents from POP locations and 159 residents from control locations were compared during the preintervention period to 162 POP residents and 177 control residents during the postintervention period. Results from OLS regression models indicated that there was no significant effect of the intervention on satisfaction with police or perceptions of violent crime, property crime, physical and social disorder, safety, or procedural justice.

### Guseynov (2010)

Guseynov (2010) evaluated a problem‐solving initiative conducted by the Kansas City Police Department's Comprehensive Strategic Team Accountability Review (CSTAR) unit. The police entered the Columbus Park area intending to target crime and quality of life offenses. They determined that abandoned buildings and public housing projects were major contributors to the crime problem in the area, and that there was a lack of partnership between the police and the community. Police responded by tearing down abandoned buildings that had been linked to drugs and prostitution. They also began prosecuting landlords that were facilitating crime and implementing strict code enforcement in the area. Other response activities included replacing destroyed parking meters to remove excuses for rule breaking, partnering with other law‐enforcement agencies to target drug dealers, removing signs of disorder, and working with community members to increase trust and cooperation with the police. Guseynov evaluated the program using a nonequivalent control group quasi‐experimental design. Weekly Index I crimes within the three police beats comprising the Columbus Park area (intervention area) were compared with the rest of the Central Patrol division. Comparisons were made for the year before, during, and after the intervention. Results from *t* test analyses indicated that weekly Index I crimes decreased significantly in both intervention and control areas over the course of the project. Guseynov also constructed an anticipated average for the treatment area based on the reduction rate in the control area. This analysis indicated that the treatment area's average weekly crime count had decreased more than would be anticipated based on the trend in the control group, and that the difference between the observed and anticipated treatment group average was statistically significant.

### Hollywood Police Department (2015)

The Hollywood, FL Police Department (2015) began receiving complaints from residents during community meetings over increases in residential burglaries. Analysis of official data confirmed rising residential burglary rates in three particular reporting areas, and at specific times of day. Police first began conducting environmental surveys of residences that had experienced repeated victimization, noting potential CPTED recommendations. They also analyzed the characteristics of arrested offenders and the types of property being stolen in the burglaries, noting trends in both offender profile and targeted property. All units of the department began meeting monthly to discuss the problem‐solving strategies in an iterative fashion. The response phase was initiated with increased patrol presence in the targeted areas during at‐risk times. Police also began making changes to the physical layout of the areas, such as closing or restricting access to alleyways, constructing deterrent signage that advertised the anti‐burglary initiative, implementing a property marking campaign for victims, and conducting security surveys with victims to recommend target hardening/surveillance measures. Other response activities included establishing neighborhood watch programs and partnering with probation to monitor prior offenders. The project assessment was conducted using a nonequivalent control group quasi‐experimental design. The three targeted reporting areas were compared with three similarly sized reporting areas that did not receive the intervention. Burglary incident counts were compared before and after the intervention for both groups. This comparison indicated that all target areas experienced decreases in burglary incidents over the course of the intervention, while all comparison locations experienced increases in burglary incidents over the same time span. There was a noted small amount of spatial displacement; however, no statistical tests were provided.

### Houston Police Department (2012)

The Antoine corridor in Houston, Texas is described as a one‐mile stretch of road surrounded on both sides by residential apartments. The Houston Police Department (2012) had been dealing with rising crime and declining occupancy in this area for several years and had made prior attempts at intervention via increased presence and enforcement but had ultimately been unable to address the problem. As the area worsened, local media coverage and citizen complaints mounted, leading the department to pursue a problem‐solving initiative to address the crime problem. Officers believed that the apartment complexes in the area were facilitating issues by renting to criminals. Analysis of crime data confirmed that certain apartment complexes in the area were responsible for disproportionate amounts of crime. Thus, the police began responding to apartment complexes one by one in an iterative SARA loop. Officers first began with strict code enforcement and partnership with other police units to execute warrant sweeps and gather intelligence on gang members. As a result, local government held dangerous building hearings, leading to the demolition of one particularly problematic complex. As police repeated this process targeting new apartment complexes, they were also able to cooperate with several government agencies to pass legislation that allowed greater enforcement and supervision activity over rental properties. Response activities also included engagement with residents and local stakeholders regarding input on area redevelopment, which lead to the construction of new area housing that was CPTED compliant. The intervention was assessed using a nonequivalent control group quasi‐experimental design that compared Part I crime counts for the intervention area and the rest of the city as a whole. Crime counts were provided for several years before and after the intervention. Crime count trends indicated that the intervention area experienced large and sustained decreases in Part I crime and narcotics cases in the postintervention period, declining by as much as 57% from preintervention peaks. The rest of the city also experienced decreases, but of far less magnitude. The authors claim that there was initial evidence of displacement that quickly gave way to diffusion of benefits; however, the report contains no associated statistical test.

### Knoxville Police Department (2002)

The Knoxville (TN) Police Department (2002) describes a program designed in response to citizen complaints about repeat offenders. These repeat offenders tended to be parolees or probationers that received limited supervision and services in the community. Working with the Tennessee Board of Probation and Parole, officers reviewed parolee records and citizen complaints, determining that past efforts such as increased patrol (more arrests) and reduced workloads had been largely unsuccessful. They recognized that these offenders re‐entering the community frequently had dysfunctional families and substance abuse and mental health problems. The two agencies created the Knoxville Public Safety Collaborative as a response, combining the resources of the police and probation services and collaborating with 25 human service providers to bring much needed services to parolees. The response involved coordinated and proactive treatment in which the parolee and parole officer developed a release plan, followed by a multi‐division staff meeting to discuss treatment options, and then the parolee supervision by a team including police officers, probation officers, and community service providers. The 265 parolees in the program were compared with a historical comparison group of 261 parolees who would have been eligible for the program. This quasi‐experimental evaluation was completed by the University of Tennessee School of Social Work. Assessment of success rates indicated that 78 (29%) program participants succeeded in not having their parole revoked while only 29 (11%) succeeded in the comparison group. Program participants were also found to be less likely to pick up new charges and receive technical violations.

### Kochel and Weisburd (2017); Kochel and Weisburd (2019); Kochel et al. (2015)

The St. Louis County Hot Spots in Residential Areas experiment evaluated the differing effects of problem‐solving, directed patrol, and standard policing on hot spots of crime. Part I and Part II crime incident data was mapped to identify both stable and active hot spots. After initial hot spots were identified, precinct commanders evaluated the potential hot spots, and ultimately 71 areas were included in the study. Using a randomized experimental design, hot spots were first separated into four blocks based on shared characteristics, and then randomly assigned from within each block to receive either problem‐solving, directed patrol, or standard policing. Randomization resulted in 20 problem‐solving hot spots and 31 control hot spots. Twenty‐two police officers were assigned to the problem‐solving areas, and problem‐solving officers were also provided a dedicated crime analyst. The analyst would provide incident and calls for service data for officers to examine during problem scanning and analysis processes, and officers were generally found to have focused on problems that accounted for the highest calls for service levels. Problem‐solving efforts were required to involve partnership with an outside stakeholder on at least one problem, and while problems differed by location, responses generally focused on either property crimes, violent crimes, quality of life offenses, or repeat address issues. Analysis data sources often included resident surveys, area observation, CPTED assessments, interviews, and discussions with community members, landlords, businesses, school representatives, etc. Problem responses also varied based on location, but included target hardening education and implementation, nuisance abatement, area cleanup, code enforcement, and communication with other agencies (among others). To evaluate the experiment, Kochel et al. (2015) used a time‐series analysis to compare trends in weekly calls for service levels before and after implementation of the intervention. ARIMA time‐series models were created for problem‐solving, directed patrol, and control locations. These models indicated that problem‐solving places experienced a statistically significant decline in calls for service per week, while control locations experienced a nonsignificant decline. Kochel et al. also conducted community surveys to assess changes across several community‐level variables. Analysis of survey responses indicated that there were some initial but short‐term decreases in police legitimacy and feelings of safety among residents in problem‐solving areas, but that there were long‐term improvements in willingness to cooperate with police, relative to control locations. Subsequent survey analyses by Kochel & Weisburd (2017, 2019) indicated that residents in problem‐solving areas exhibited delayed but increased willingness to cooperate with police and increased informal social control relative to residents in standard policing communities. However, these residents/areas did not display significant changes in perceptions of procedural justice, police abuse, legitimacy, social cohesion, or collective efficacy.

### Lancashire Constabulary (2008)

In 2005 a new neighborhood policing team took over the Farringdon Park area of Preston, England. Official data indicated that Farringdon Park was one of the most deprived neighborhoods in England, and that the area suffered from disproportionately high levels of crime. Concerns over crime and disorder in the area were highlighted by elected representatives, local community, and press reports. Police analyzed official data as well as data from the primary landlord association in the area, determining that criminal damage was becoming a major economic and environmental cost. Surveys were carried out to assess neighborhood residents’ thoughts on crime issues in the area and receptivity to the formation of a community group. The police held meetings with residents and local representatives and carried out environmental surveys with cooperation from service providers and other stakeholders. Project staff also met with the head of criminology at the University of Central Lancashire and reviewed relevant criminological theory. Based on this analysis, the response phase consisted of various enforcement, situational, and social crime prevention tactics. Enforcement measures included warrant execution, high profile arrests, evictions, and hot spot patrol, among others. Situational measures included target hardening of various access points, area cleanup, lighting improvements, CCTV funding, and road redesign. Social measures involved restorative justice, outreach work by youth services, recreational opportunities, and other youth intervention schemes. The project was evaluated with a nonequivalent control group quasi‐experimental design. Crime incident and calls for service data for the intervention neighborhood were compared with another neighborhood with similar characteristics that did not receive the intervention. Incident and calls for service counts were presented both before and after the intervention. Comparison of raw differences for treatment and control locations indicated that the target area experienced a sizable decrease in crime counts over the course of the intervention, while the comparison area experienced a slight increase. Furthermore, while both areas also experienced a decline in call numbers, the target area's decline was notably larger. The authors assessed displacement/diffusion by examining crime and call rates in an adjoining neighborhood. This analysis suggested no evidence of displacement, but rather evidence of diffusion of benefits. However, no test of statistical significance was included in the report. Monetary savings were also examined through comparison of yearly crime costs. Savings for the postintervention year were reported to be as much as $220,467.

### Lancashire Constabulary (2012)

Lancashire police became aware of public concern regarding juvenile crime and delinquency in Preston, England. While the number of first arrests among juveniles was decreasing slightly, police observed a lack of resources for at‐risk youth and were concerned that existing resources would continue to decline due to budget cuts. Thus, police felt that juvenile arrests would begin to increase without proper intervention. In the analysis phase, police examined national trends and economic costs associated with youth offenders. They also analyzed the characteristics of the youth offenders, victims, and locations of offenses within the city, as well as literature on youth interventions. The response drew inspiration from the American “Scared Straight” program, but with a focus on early intervention and education rather than fear. An asset scoring system was created wherein police intervention with youth would trigger the allocation of points for the individual involved. As points accumulated, the youth's record would be reviewed at weekly meetings between police and other project partners. When enough points accumulated, the “Custody Experience” would be initiated. This intervention involved collecting the individual from their home and taking them to the police station. Once there, the youth would be given a scenario of possible arrest and educated about the custody process, consequences, and threats associated with their actions. The intervention was designed to be tailored to the specific circumstances of the individual, and with the intention to provide a positive and preventative experience. The project was assessed using a nonequivalent control group quasi‐experimental design. Youth reprimand counts for the entire intervention city were compared against two nonintervention cities that were selected based on similar geographic and financial characteristics. Reprimand counts were provided for both pre‐ and postintervention years. Between the year prior to, and the year following, the intervention, reprimand rates in the target city decreased by 33% while reprimand rates in the two comparison cities rose by 11% and 14%, respectively. The authors also suggest that the decrease in reprimands equated to a cost savings of £82,000.

### Lexington Division of Police (2009)

In 2006 the former and current Police Chiefs of the Lexington Division of Police commissioned a historical analysis of the department's official crime data. During this review, the department's analysis unit identified seven neighborhoods with disproportionate amounts of reported crime and calls for service. The problems in these neighborhoods were also frequently recognized by local government, residents, and police observations. To address these problematic neighborhoods police began analyzing UCR data, as well as victim and resident surveys to further determine community perceptions and concerns. Police determined that these neighborhoods were primarily multi‐housing units, but that local business operations had been declining, leading to increased vacancy. They further determined that there was a prevailing perception that the majority of offenders resided within the community, rather than outside of it. Additional analysis activities specific to particular neighborhoods included consultation with government agencies, social services, and inspectional services. In the response phase, police conducted meetings with various government and community organizations to discuss strategies and foster communication. Neighborhood response officers began engaging in directed patrols and other proactive techniques focusing on problem offenders. Other response measures included code inspection and enforcement at high crime addresses, construction of deterrent signage, area cleanup, fence, and streetlight repair, and other situational measures. The project assessment was conducted with a nonequivalent control group quasi‐experimental design. Reported crime counts for the seven intervention neighborhoods were compared with reported crime counts for the rest of the city. Counts were provided for 2 years before and 2 years after the year of project implementation. Trends in the data suggested that reported crime decreased in the seven intervention neighborhoods by an average of 8%, while reported crime in the rest of the city (or neighborhoods not receiving intervention) decreased by only 1%. This finding is also used as evidence that there was no displacement of crime, as the remainder of the city did not experience a subsequent crime increase. However, no statistical test of displacement was included.

### London Borough of Enfield (2011)

Enfield's Community Safety Partnership (CSP) noticed an increase in burglary offenses during 2008, after rates had been stable for several years prior. Meetings with residents, community safety surveys, and local media coverage further indicated that burglary was a major concern among the public. The CSP was also concerned with the economic and psychological effects that burglary can create, leading to the implementation of a problem‐solving initiative designed to address it. Project staff mapped and analyzed official data, noticing burglary concentrations in specific hot spots, seasonal burglary trends, and consistencies in the characteristics of the offenses (i.e., method of entry). The partnership similarly assessed the environmental characteristics of the highest frequency areas, noting physical security risks that needed to be addressed. There was also analysis of criminological literature and information on prior burglary offenders in the area, including offenders that had been apprehended and subsequently provided rationale for their offense. Ultimately, the project staff felt that they had an actionable understanding of the environmental characteristics of locations that predisposed them to repeat victimization. Thus, the response primarily involved situational crime prevention measures such as installing window and door locks, controlling access to alleyways, distribution of timer switches, low‐watt bulbs, and shock alarms. Residents were also given the resources to mark property and provided crime prevention literature. Further response activities included publicization of burglary awareness and graffiti and area cleanup. The intervention was assessed using a nonequivalent control group quasi‐experimental design. Houses that received the intervention were aggregated and compared with all other nonintervention houses in the borough. Burglary incident counts were used to compare the treatment and comparison groups both before and after the intervention. This evaluation indicated that intervention houses experienced 78.7% fewer burglaries in the postintervention period than the preintervention period. Conversely, nonintervention houses only experienced 2.1% fewer burglaries over the same time period. The authors also suggest that, when factoring in the economic and social costs associated with burglary, the intervention was responsible for generating a cost savings of £934,000.

### Mazerolle et al. (2000)

Mazerolle et al. (2000) describe a randomized experiment testing the impact of the Beat Health POP program in Oakland, CA. The Beat Health program was designed to address drugs and disorder at problem addresses/street blocks in the city. Sites were referred to the Beat Health team through hotlines, community meetings, and reviews of calls for service. Half of the sites (50) referred were randomly selected to receive the Beat Health treatment; the other half (50) received normal patrol. The analysis used a blocked design to compare residential and commercial addresses separately. The Beat Health intervention involved a team of one police officer and one police service technician visiting a site to identify and analyze the problem and to make contact with the property owner or place manager to try to address the problems. The police attempted to build a close working relationship with individuals who had a stake in improving the property and tried to provide guidance on crime prevention. The intervention typically involved pressuring third parties (usually the landlord of a problem apartment building or property owner) to make changes to improve property conditions.

The Beat Health team could also use the SMART (Specialized Multi‐Agency Response) Team, made up of city inspectors, to enforce local housing, fire, and safety codes. The team could also instigate legal action against landlords and property owners through civil law. This project used a problem‐oriented approach to third‐party policing: Beat Health teams met with property owners and closely examined problem sites to determine the best course of action to target problems.

Calls for service data were used for the assessment. Comparing call data before the intervention to a 12‐month postintervention period, percentage change and mean assessments indicated that experimental sites exhibited a significant decrease in drug calls, primarily driven by decreases at residential sites. In contrast, control sites experienced a significant increase in drug calls, and this was primarily driven by increases at commercial sites. There were no significant decreases in violent crime, disorder, or property crime calls in either experimental or control groups, though disorder calls in the experimental group did decline significantly at commercial sites relative to residential sites. Mazerolle et al. measured displacement/diffusion by examining drug calls for service within 500‐foot catchment areas around each target address. This analysis revealed statistically significant evidence of spatial displacement into the catchment areas at commercial sites, particularly for the control group. There was also some evidence of diffusion of benefits in the residential catchment areas for the experimental group, however, this did not appear to be statistically significant.

### Niagara County Sheriff's Office (2011)

The Niagara County Sheriff was contacted by a New York State Senator's office on behalf of the Newfane Business Association. The association was expressing concerns about economic repercussions associated with increasing crime and disorder along the Main Street corridor in the town of Newfane, New York. The small rural town was suffering from visible drug dealing, vacant buildings, and general area blight. Police began by partnering with community members to increase information sharing. This partnership consisted of five officers and eight civilians representing local school, church, and government organizations. Analysis of official data indeed revealed disproportionate trends in CFS rates, with patterns at specific locations and times. During information sharing meetings, members of the partnership discussed these trends and the characteristics of local suspects and problem addresses. It was further determined that problem areas along Main Street were multi‐unit housing complexes with detached landlords. Based on the information learned, the Sheriff devised a multi‐point action plan that involved intensive police enforcement (zero tolerance methods), curfew enforcement for youth, investigation of suspected drug dealers/users, partnership with landlords to evict drug dealers and clean graffiti, code enforcement, warrant sweeps, and other situational and enforcement based responses. The assessment followed a nonequivalent control group quasi‐experimental design by comparing reported crime counts for Newfane to a similar nearby town that did not receive intervention efforts. Crime counts were compared four weeks prior to, and 4 weeks proceeding, the intervention. Results showed that reported crime decreased by 60% in the intervention town, while reported crime decreased by only 7% in the comparison town.

### Nunn et al. (2006)

The Brightwood neighborhood in Indianapolis (IN) had been known to be experiencing problems related to drug trafficking and associated violent crime. In 1995 and 1996 surveys with community residents were conducted. These surveys indicated that, not only was there a concentration of crime in and around the Brightwood area, but that residents overwhelmingly felt that the problem was related to drugs. During problem analysis, officers from the Metro Drug Task Force (MDTF) began observing the Brightwood area, trying to identify drug dealers based information they had received from neighborhood and crime watch associations. Through communication with community members and other district police, MDTF officers were able to compile a list of suspected drug dealers in the area. Officers then began pulling arrest records and criminal histories on these offenders to build intelligence files. The MDTF partnered with the FBI to conduct surveillance, both in‐person and through the use of wiretaps. Once enough evidence had been gathered, MDTF officers began to obtain search, arrest, and seizure warrants. The physical response consisted of a targeted raid and warrant sweep in the Brightwood area, resulting in the arrest of 21 individuals considered to be major drug dealers. To evaluate the interdiction a nonequivalent control group quasi‐experimental design was used. Calls for service data in the Brightwood neighborhood were compared with calls for service data in a nearby neighborhood (Westside). The comparison neighborhood had similar economic characteristics and a similar number of residents between the ages of 14 and 29. Call numbers were measured preintervention and for each of the following 2 years after the intervention. Analysis of *t* test results indicated that the treatment neighborhood experienced significant decreases in calls for overall serious crime (significant in both postintervention years), burglary (only significant in the second postintervention year), gun crime (both postintervention years), personal violence (both postintervention years), and robbery (both postintervention years). In contrast, the comparison neighborhood only witnessed significant decreases in calls for overall serious crime (limited to first postintervention year) and gun crime (limited to first postintervention year). Results for theft calls were nonsignificant for both groups, while drug calls initially increased in the intervention area and decreased in the control area, though these differences were also nonsignificant (in year two drug calls returned to near preintervention levels in both areas).

### San Angelo Police Department (2006)

In 2004, the San Angelo (CA) Police Chief asked officers to determine salient problems facing the community. Through these discussions, an increase in reported forgeries was brought to the Chief's attention. The department believed that the rise in forgeries was likely attributable to repeat offenders with drug addictions, and that, through addressing this problem, other crime and safety issues may also be improved. To analyze the problem, police relied on official data, tracking the increase in forgeries over the prior 2 years. The department initially created a fraud unit that relied on traditional enforcement responses, but this approach proved to be ineffective. Reassessing the issue, the police noted that the primary victims of reported forgeries were businesses rather than individual citizens, and thus switched their focus to proactive intervention with city businesses. The police chief initially held meetings with representatives from the most repeatedly victimized businesses, advising them to implement a customer identification checking procedure. When this approach was met with resistance, it was determined that the more effective response would be to involve the customers. Police developed the “See! It's me!” program designed, primarily, to encourage customers to proactively protect themselves from identity theft. The program consisted of distinct stages: in the public education stage, the threat of identity theft was advertised on television, radio, and billboards. In the retailer training stage, the police provided businesses with program advertisement materials to place in their stores and established a standardized identification checking procedure. In the financial institutions stage, banks and credit unions began advising their customers to limit personal information printed on checks and take other protective measures. Two Walmart stores subsequently embraced the program and were used as the treatment group for impact evaluation. The assessment was quasi‐experimental in nature and compared the two Walmart stores that adopted the program to two other businesses that did not implement the program properly or at all. Reported forgery counts were compared between the two groups for several months before and after program implementation. Both intervention Walmart stores experienced a notable decrease in average reported forgeries per month. In contrast, one of the two comparison businesses reported an increase in average forgeries, and the other remained nearly stable over the same 12‐month time period. Additionally, citywide average monthly forgeries were reported to have decreased by 34% in the 5 months following the evaluation period compared with the previous year average. The authors suggested that offenders were displaced to other businesses as a result of the intervention at Walmart stores; however, no statistical test was discussed or presented.

### Sherman et al. (1989)

Sherman and associates (1989) describe the Minneapolis, MN Repeat Call Policing (RECAP) program designed to respond to commercial and residential addresses with a high number of calls for service. Using calls for service data, the top 500 addresses with the most calls were examined. Schools, city hall, hospitals, police stations, parks, check‐cashing locations, and intersections were all removed because police felt these locations were inappropriate for the intervention. The remaining sites were blocked into half commercial (250) and half residential sites (250). These sites were then randomized in rank‐ordered pairs with half of the sites assigned to receive a POP treatment and half to receive standard patrol. After some data cleaning issues, a total of 119 residential sites and 107 commercial sites received the treatment. The treatment team was four officers and a sergeant who were assigned to visit each site and use as many sources as possible to diagnose the problem. These sources included analysis of call data and incident reports, on‐site interviews of residents, and interviews of place managers. Officers were then supposed to design and implement an intervention plan that needed to be approved by the sergeant. The actual treatment varied greatly across addresses. Officers spent a lot of time helping landlords with problem tenants and providing letters to repeat domestic violence victims informing them of their rights and available services. Commercial responses were even more heterogeneous than residential responses. The time spent at each site also varied considerably with officers visiting some addresses only once and others weekly throughout the yearlong intervention period. The program was assessed using a comparison of calls for service data. Pre–post call trends indicated that, in the first 6 months of the program, target residential addresses experienced a statistically significant decrease in calls, however during the second 6 months of the program no call reduction was observed. Additionally, during this second 6‐month period a significant reduction in calls at commercial addresses favored the control group. After the full experimental year, target residential addresses displayed a nonsignificant 6% reduction in calls compared with a 0.1% increase at control addresses, while there were no notable overall differences between treatment and control at commercial addresses.

### Stokes et al. (1996)

Stokes and associates (1996) document a POP project designed to reduce student victimization on the way to and from middle school in Philadelphia, PA. Officers recognized that school violence was an issue, and they worked to understand the underlying problems. Using focus groups, victimization surveys, and analysis of police and school data, the police, along with representatives from the Center for Public Policy at Temple University and vice‐principals from Philadelphia middle schools all came to better understand the dynamics of students being attacked on their way to or from school. They used crime mapping to visually display unsafe locations identified by students and the student victimization survey provided data on the level of victimization, how often this victimization was reported, and how dangerous students perceived their trip to and from school to be. Using this data, the Philadelphia Police Department decided to create a police‐secured safe corridor for students to travel on foot safely to one middle school. Using officers from the Philadelphia Police Department, the Temple University Police Department, and the Philadelphia Housing Authority, the police used crime maps to create a corridor 10 blocks long and three blocks wide where police patrols were increased from 8 to 9 am and 2:30 to 4 pm.

During these time periods, two foot patrol officers, a patrol car, and a bike patrolled the corridor.

A pre‐ and poststudent victimization survey was used for the assessment. Student responses in the target middle school were compared with responses from students in three similar middle schools after the 6‐week intervention period. Simple pre–post comparison of survey results revealed that the percentage of students from the test school who reported being attacked increased from 19.4% to 20.2%, while the same measure for students from control schools decreased from 21.2% to 15.2%. Students from test schools also indicated a slight increase in feelings that they would be picked on or bothered (from 32.4% to 33.4%) while control students indicated a slight decrease (from 30.4% to 28.4%). Analysis of variance tests indicated that the increase in reports of being attacked or bothered was nonsignificant in the test school, however, the decrease on the same measure in the control schools was statistically significant. Further, there were no significant differences for either group in fear of being bothered or attacked between pre‐ and postintervention surveys. Results also indicated that there appeared to be little knowledge of the intervention, as only 27.4% of students at the test school reported being aware of the corridor.

### Stone (1993)

Stone (1993) describes a POP project in Atlanta, GA designed to address drug selling and use in public housing projects. Two housing projects were chosen as intervention sites and two were used as comparisons in this quasi‐experiment. To analyze the drug problems, a management team was created with representatives from the Atlanta Police Department and the housing authority. The management team conducted resident victimization surveys to determine the extent of problems and understand resident perceptions of crime problems. The research team, along with the police, conducted extensive research to document the drug problem in the area by examining data from the police, drug treatment facilities, schools, courts, social service agencies, and corrections agencies. The management team focused on five problem areas in the response: poor lighting, abandoned cars, abundant litter, poor playgrounds, and improperly strung clotheslines. These five problems were identified by residents, officers, and supervisors, and the management team thought focusing on these problems would help address some of the underlying issues leading to drug problems. There was also an effort to get uniformed officers to work more closely with undercover narcotics detectives and to have all officers work more cooperatively with the Atlanta Housing Authority. The team did successfully work with Georgia Power to implement weekly lighting checks, abandoned cars were quickly removed, resident cleanup days reduced the litter problem, and dangerously strung clotheslines that could get in the way of officers were quickly repaired. The program was assessed using pre and post victimization data on whether residents in the target and comparison housing projects had been asked to buy or sell drugs. Analysis of variance tests for the year before and after program implementation indicated that residents’ in both treatment and comparison areas reported significant increases in victimization from violent crime and being asked to buy/sell drugs; however, there was a significantly higher increase in treatment sites relative to controls. Conversely, based on difference of proportions tests using official data, there were no significant differences in overall crime and property crime between the target and comparison areas, and the comparison areas experienced an overall significant increase in violent crime and drug arrests relative to the target areas.

### Taylor et al. (2011)

Taylor et al. (2011) conducted a randomized controlled trial of hot spot policing strategies in partnership with the Jacksonville (FL) Sheriff's Office (JSO). With a noted increase in violent crime in recent years, the team mapped official crime data to identify hot spots of street violence (nondomestic violence). This process led to the identification and selection of 83 hot spots with varying land use characteristics. Using block randomization, the hot spots were separated into four groups based on violent incident levels, and randomly assigned to receive either standard policing (control), directed patrol, or POP. In total, 40 areas were assigned to control and 22 areas were assigned to receive the 90‐day POP intervention. A 100‐foot buffer zone was also constructed around each hot spot to measure displacement. The POP areas were provided a total of 60 assigned police officers and four dedicated crime analysts who were to be engaged in problem‐solving activities full‐time. Officers tackled problems in small teams and were advised to determine the root cause of violence in their area. POP teams often worked closely with community members and focused on an array of issues such as specific offenders, the community, and environmental factors. Specific responses varied by area, but common measures included situational strategies (repairing fences and lighting, constructing road barriers) and collaboration with businesses/property managers to address security issues. Other responses involved community surveys and outreach, providing youth with recreational opportunities, area cleanup, nuisance abatement, and code enforcement. Additionally, while some enforcement measures were used, officers were encouraged to focus on preventative responses. To evaluate the experiment, both violent and property crime incident data and calls for service were used. These measures were compared across POP, directed patrol, and control groups both during and after the intervention period. Poisson and negative binomial regression models indicated that nondomestic violence decreased significantly in POP hot spots relative to controls, but only during the postintervention period, and only as measured by incident data. No significant differences were found in either incident counts or calls for service during the intervention period. Additionally, no significant differences were found for property crime, or violent crime as measured by call data, in the postintervention period. Taylor et al. also found statistically significant evidence of displacement of violent calls for service, but no significant evidence of incident displacement. The authors suggest that residents in the buffer areas may have become aware of the POP intervention, resulting in an increased willingness to call the police.

### Thomas (1998)

Thomas (1998) describes the Coordinated Agency Network (C.A.N.) designed to reduce juvenile probationer recidivism in San Diego. The San Diego Police and the San Diego County Probation Department Juvenile Division both recognized that juveniles were frequently being rearrested after release on probation. In San Diego, low‐risk juvenile offenders were typically “banked,” meaning they only had to contact their probation officer by mail. They were largely unsupervised and frequently failed to abide by the conditions of their probation. An analysis of the area revealed that many of these juveniles needed greater supervision because of unstable family lives, and because of their close geographic proximity to major drug ports, gang activity, and a large prison. The police and probation division formed C.A.N. to increase supervision and monitoring of juvenile probationers. Fifteen officers volunteered to help monitor the juveniles and to refer them and their families to community‐based support programs. After an initial assessment by a senior probation officer, police officers assigned to each juvenile would make biweekly visits to be supervisors and mentors. The program included a graduated model of sanctions and rewards based on the juvenile's compliance with probation along with their performance at school. For the assessment, recidivism rates for a group of 80 C.A.N. participants were compared with a group of 80 similar “banked” juveniles who did not participate. Basic comparison of frequencies indicated that individuals in the treatment groups experienced a much lower rate of recidivism (6%) relative to the comparison group (22%). Additionally, 27% of program participants successfully completed probation, compared with 20% in the comparison group.

### Tuffin et al. (2006)

Tuffin et al. (2006) report on the National Reassurance Policing Programme implemented in six wards (neighborhoods) in the United Kingdom. The program was designed to address the “reassurance gap,” the idea that residents are fearful of increasing crime rates even when crime is actually decreasing. This gap has been explained in part by the signal crimes perspective, which argues that certain crimes, particularly certain types of disorder, signal to the community that crime is out of control. Thus, the rates of these signal crimes are more important in generating resident perceptions than actual overall crime rates. The program had three main focuses: having accessible and visible police officers, community involvement in identifying priorities for police, and using targeted police activity and problem solving. A seven‐stage model was used to implement the program: *Research*‐ officers had to find out about the neighborhood and how to engage residents; *Engage*‐ police needed to create conditions for dialogue; *Public preferences‐*officers used surveys, questionnaires, neighborhood meetings, and visual audits to better understand problems facing the community; *Investigation and analysis*‐police used meetings and focus groups to give a deeper analysis to identified problems; *Public choices*‐ the police presented the findings of their analysis to residents, so the community could choose priorities; *Plan and action*‐officers developed and implemented a plan with local partners; *Review*‐ police completed an assessment of the problem. The specific problems targeted varied by ward, but all included some type of antisocial behavior, and typically involved drug problems. The researchers used total recorded crime as a method of assessment, comparing each target site to a similar comparison ward before and after the implementation of the program. Evaluation of the 12‐month preintervention and intervention periods indicated that two of six treatment sites experienced significant decreases in reported crime relative to their matched comparison sites. One matched pair demonstrated a significant reduction in favor of the comparison site, and the other three pairs demonstrated nonsignificant changes.

### Vancouver Police Department (2009)

The Granville Entertainment District (GED) in Vancouver, Canada is a nightlife area consisting of both businesses and residential complexes. In 2002 a liquor policy was passed that increased the hours of service within the district. Shortly after, the Vancouver Police Department began to observe increased street‐related crime and disorder in the area. The department initially responded by increasing law‐enforcement presence but were unable to stabilize the problem. Negative media portrayal, public complaints, and officer observations all suggested that an alternative solution was needed. Police began by analyzing official crime data, noticing a significant increase in street crime/disorder‐related calls for service in the GED. Official data also indicated that the primary offenders and victims of these incidents were both intoxicated young adults. Officers also conducted environmental observations of the area, noting a lack of transportation options, overcrowded walkways, gang presence, and problem establishments. The department additionally sought input from the Bar Association, a local bar industry group, during the analysis phase. To respond to the problem, police decided to move away from the traditional enforcement measures that had been unproductive in the past. Importance was placed on increasing communication with the local Bar Association, leading to a line restriction at bars after 2:00 am. Proactive police patrol was increased in the area, encouraging officers to be more mobile and interact with patrons. Police also partnered with local radio to advertise safe practices in the GED and partnered with taxi services to increase transportation options. The response redeveloped overcrowded sidewalks by closing off main through streets on weekend nights, and constructed deterrent signage reminding patrons of police presence in the area. While the majority of response activities were proactive, the police did partner with the Firearm Interdiction Team to target gang members in the GED and conducted an undercover operation to monitor liquor law compliance at local bars. The assessment followed a nonequivalent control group quasi‐experimental design. Calls for service numbers for the GED were compared with two other popular nearby entertainment districts. Comparisons were provided both pre‐ and postintervention. Simple analysis of percentages indicated that the intervention area witnessed a 20% decrease in calls for service after the intervention, relative to before. In contrast, one of the comparison districts experienced a decrease of only 4%, while the other comparison district experienced a 46% increase. Increases in the call rates of nearby areas led the authors to suggest that there was minimal displacement, however no statistical test of displacement was presented.

### Weisburd and Green (1995)

Weisburd and Green (1995) evaluated the Jersey City, NJ Drug Market Analysis Program. The program identified 56 hot spots of high‐activity drug dealing. These hot spots were identified using narcotics sales arrests, drug‐related calls for service, narcotics tip‐line information, and the assessments of narcotics detectives. Half of these hot spots were randomly assigned to a POP treatment and half received routine enforcement that relied primarily on arrest. The cases were randomized in four statistical blocks, based on volume of drug activity. Two‐block catchment areas were constructed around each hot spot for measurement of displacement. The program recognized from the outset the need to assign specific officers to specific hot spots to increase accountability, and the need to allow for a diversity of responses to address the problems at a specific hot spot. The program included a step‐wise process similar to the SARA model. In the planning stage, officers collected data on the physical, social, and criminal characteristics of each area. In the implementation stage, officers coordinated efforts to conduct a crackdown at the hot spot and use other relevant responses to address underlying problems at the hot spot. Finally, in the maintenance stage, officers attempted to maintain the positive impact of the crackdown. To implement the experiment, squads of narcotics officers were randomly assigned to the treatment or control hot spots. The assessment used calls for service data for the 7‐month pre‐ and postintervention periods. An analysis of variance model indicated that a significant difference in overall disorder‐related calls favored the treatment group. While both treatment and control areas experienced increases, the control areas’ increase was significantly greater. The model showed no significant differences between the two groups on violent or property calls, and the measurement of narcotics calls was deemed to be unreliable and omitted from the results. Displacement analysis results suggested a diffusion of benefits into experimental catchment areas for both public morals and narcotics calls. The emergence of new hot spots was also determined to be nearly two times more likely to occur in control catchment areas than experimental catchment areas.

### Zidar et al. (2017)

During an interview for a crime analyst position with the Paducah (KY) Police Department (PPD), Zidar was asked to analyze local crime data. During the process, Zidar noticed that incidents at local Walmart stores were expending a disproportionate amount of officer time and resources. After being hired, the PPD asked Zidar to pursue this issue and he began riding with patrol officers, talking with community members, and examining official crime data. He determined that Walmart stores were accounting for a disproportionate number of shoplifting incidents and that there was a sense within the department that little could be done. During analysis, the characteristics of shoplifting offenses were examined, and the physical layout of the stores was assessed. The analysis suggested there were too few elements of guardianship or natural surveillance in the stores, and that the result was an environment conducive to theft. Police representatives met with Walmart management and provided recommendations for redesign of the physical environment and the management practices. Based on these meetings, it was determined that Walmart management felt little responsibility for the prevention of theft, and that other response options would need to be pursued. In response, the PPD altered the reporting practices for shoplifting incidents at the two local Walmart locations. They created an online reporting system that was to be used by Walmart loss prevention for any shoplifting incidents in which the reported value stolen was less than $500. This system forced the store to bypass the PPD and take these cases directly to the County Attorney, rather than receive police response (unless violence was used, or loss‐prevention could not determine the identity of the offender). This response was an effort to force Walmart management to take responsibility, and simultaneously free up police resources. Walmart subsequently coordinated with a third‐party vendor to implement a restorative justice program giving offenders the option of bypassing formal case‐processing. The evaluation of the program followed a nonequivalent control group quasi‐experimental design. Reported larcenies under $500 were measured for the two Walmart stores in the intervention area and compared with four nearby Walmart stores, and a nearby mall, that did not receive the online reporting system. Independent *t* tests indicated that reported larcenies decreased significantly at the intervention Walmart stores between pre‐ and postintervention periods. One comparison location also witnessed a significant decrease, while another witnessed a significant increase, in reported larcenies. The other three stores experienced nonsignificant changes. Zidar et al. (2017) also conducted a cost/benefit analysis, comparing average monthly time and monthly cost associated with theft‐related calls at local Walmart stores. Results indicated a statistically significant decrease in mean monthly cost and time related to these calls during the postintervention period relative to the preintervention period .

## APPENDIX B GPD SYSTEMATIC SEARCH STRATEGY ^14^ Appendices B and C are taken directly from Higginson, A., Eggins, E., Mazerolle, L. and Stanko, E. (2015). *The Global Policing Database [Database and Protocol]*.

### Search Terms

To ensure optimum sensitivity and specificity, the GPD search strategy utilizes a combination of free‐text and controlled vocabulary search terms. Because controlled vocabularies and search capabilities vary across databases, the exact combination of search terms and field codes are adapted to each database.

The free‐text search terms for the GPD are provided in Table B1 and are grouped by substantive (i.e., some form of policing) and evaluation terminology. Although the search strategy may vary slightly across search locations, it follows a number of general rules:

- Search terms are combined into search strings using Boolean operators “AND” and “OR.” Specifically, terms within each category are combined with “OR,” and categories will be combined with “AND.” For example: (police OR policing OR “law#enforcement”) AND (analy* OR ANCOVA OR ANOVA OR …).
- Compound terms (e.g., law enforcement) are considered single terms in search strings by using quotation marks (i.e., “law*enforcement”) to ensure that the database searches for the entire term rather than separate words.
- Wild cards and truncation codes are used for search terms with multiple iterations from a stem word (e.g., evaluation, evaluate) or spelling variations (e.g., evaluat* or randomi#e).
- If a database has a controlled vocabulary term that is equivalent to “POLICE,” the term is combined in a search string that includes both the policing and evaluation free‐text search terms. This approach ensures that the search retrieves documents that do not use policing terms in the title/abstract but have been indexed as being related to policing in the database. An example of this approach is the following search string: (((SU: “POLICE”) OR (TI,AB,KW: police OR policing OR “law*enforcement”)) AND (TI,AB,KW: intervention* OR evaluat* OR compar* OR …)).
- For search locations with limited search functionality, a broad search that uses only the policing free‐text terms is implemented.
- Multidisciplinary database searches are limited to relevant disciplines (e.g., include social sciences but exclude physical sciences).
- Search results are refined to exclude specific types of documents that are not suitable for systematic reviews (e.g., newspapers, front/back matter, book reviews).

**Table B1:** Free‐text search terms for the GPD systematic search

Policing search terms	Evaluation search terms			
police	analy*	data	outcome*	result*
policing	ANCOVA	effect*	paramet*	“risk#ratio*”
“law*enforcement”	ANOVA	efficacy	“post‐test”	sampl*
constab*	“ABAB design”	eval*	posttest	“standard deviation*”
detective*	“AB design”	experiment*	“post test”	statistic*
sheriff*	baseline	hypothes*	predict*	studies
	causa*	impact*	“pre‐test”	study
	“chi#square”	intervent*	pretest	survey*
	coefficient*	interview*	program*	“systematic review*”
	“comparison condition*”	longitudinal	“propensity score*”	“t#test*”
	“comparison group*”	MANCOVA	quantitative	“time#series”
	“control condition*”	MANOVA	“quasi#experiment*”	treatment*
	“control group*”	“matched group”	questionnaire*	variable*
	correlat*	measure*	random*	variance
	covariat*	“meta‐analy*”	RCT	
	“cross#section*”	“odds#ratio*	regress*	

### Search Locations

To reduce publication and discipline bias, the GPD search strategy adopts an international scope and involves searching for literature across a number of disciplines (e.g., criminology, law, political science, public health, sociology, social science and social work). The search captures a comprehensive range of published (i.e., journal articles, book chapters, books) and unpublished literature (e.g., working papers, governmental reports, technical reports, conference proceedings, dissertations) by implementing a search strategy across bibliographic/academic, gray literature, and dissertation databases or repositories.

It is noted that there is substantial overlap of the content coverage between many of the databases. Therefore, the *Optimal Searching of Indexing Databases* (OSID) computer program (Neville & Higginson, 2014) has been used to analyze the content cross‐over for all databases that have accessible content coverage lists. OSID analyses the content coverage and creates a search location solution that provides the most comprehensive coverage via the least number of databases. Another advantage of using OSID when designing a search strategy is the reduction in the number of duplicates that would need to be removed prior to the screening phase. Databases with >10 unique titles are searched in full, whereas databases with ≤10 unique titles were searched only the unique titles and any non‐serial content (e.g., reports, conference proceedings). Where a modified search of a database would be more labor‐intensive than a full search and export results, a full search of the database is conducted. The final search locations and solutions are reported in Table B2.

**Table B2:** GPD search locations and protocol (January 1, 1950–December 2018)

Indexed and academic databases		Content coverage fed into OSID?	Full or modified search?	Search modifications
ProQuest	Criminal Justice	Yes	Full	None
	Dissertation and Theses Database Global	Not Available	Modified	Social Sciences subset
	Political Science	Yes	Full	None
	Periodical Archive Online	Yes	Full	None
	Research Library	Yes	Modified	Social Sciences subset
	Social Science Journals	Yes	Full	None
	Sociology	Yes	Modified	Search 2 unique journal titles and non‐serial content only
	Applied Social Sciences Index and Abstracts	Yes	Full	None
	International Bibliography of the Social Sciences	Yes	Full	None
	Public Affairs Information Service	Yes	Full	None
	Social Services Abstracts	Yes	Modified	Search 5 unique journal titles and non‐serial content only
	Sociological Abstracts	Yes	Full	None
	Worldwide Political Sciences Abstracts	Yes	Modified	Search 9 unique journal titles and non‐serial content only
EBSCO	Academic Search Premier	Yes	Full	None
	Criminal Justice Abstracts	Yes	Full	None
	EconLit	Yes	Full	None
	MEDLINE with Full‐Text	Yes	Full	None
	Social Sciences Full‐Text	Yes	Full	None
OVID	International Political Science Abstracts	Not Available	Full	None
	PsycARTICLES	Yes	Modified	Search 4 unique journal titles only
	PsycEXTRA	Not Available	Full	None
	PsycINFO	Yes	Full	None
	Social Work Abstracts	Not Available	Full	None
Web of Science	Current Contents Connect—Social and Behavioural Sciences Edition	Yes	Modified	Search 1 unique journal title and non‐serial content only
	Book Citation Index (Social Sciences and Humanities)	Not Available	Full	None
	Conference Proceedings Citation Index (Social Sciences and Humanities)	Not Available	Full	None
	Social Science Citation Index	Yes	Full	None
Informit	Australian Attorney General Information Service	Yes	Full	None
	Australian Criminology Database (CINCH)	Yes	Full	None
	Australian Federal Police Database	Yes	Full	None
	Australian Public Affairs Full‐Text	Yes	Full	None
	DRUG	Yes	Full	None
	Health & Society Database	Yes	Modified	Search unique journal titles and non‐serial content only
	Humanities and Social Sciences Collection	Yes	Full	None
Gale‐Cengage	Expanded Academic ASAP	Yes	Full	None
Standalone and open access databases	Cambridge Journals Online	Yes	Modified	Search 4 unique journal titles in Law and Political Science collections and full search of Social Studies collection
	Directory of Open Access Journals	Yes	Full	None
	HeinOnline	Yes	Modified	Law Journals Online collection only
	JSTOR	Yes	Modified	Search unique titles across the Law, Political Science, Public Health, Public Policy, Social Work and Sociology collections only. The Criminal Justice collection had no unique content and so will be excluded from the search. Only 10% of content in this database have abstracts and a full‐text search returns >250,000 results because of inability to construct complex search strings. Therefore, a modified search of the unique titles across these collections will be more pragmatic than a full search of the database
	Oxford Scholarship Online	Yes	Full	None
	Sage Journals Online and Archive (Sage Premier)	Yes	Modified	Search 5 unique journal titles and non‐serial content only
	ScienceDirect	Yes	Full	None
	SCOPUS	Yes	Full	None
	SpringerLink	Yes	Full	Although this database has low uniqueness when combined with the full set of databases, a full search using only the policing search terms will be more pragmatic than a modified search on unique titles because of the restricted search functionality of this database
	Taylor & Francis Online	Yes	Modified	Although this database has low uniqueness when combined with the full set of databases, a full search using only the policing search terms will be more pragmatic than a modified search on unique titles because of the restricted search functionality of this database
	Wiley Online Library	Yes	Full	None
	California Commission on Peace Officer Standards & Training Library	No	Full	None
	Cochrane Library	No	Full	None
	CrimeSolutions.gov	No	Full	None
	Database of Abstracts of Reviews of Effectiveness (DARE)	No	Full	None
	FBI—The Fault (Reports and Publications)	No	Full	None
	Evidence‐Based Policing Matrix	No	Full	None
	International Initiative for Impact Evaluation Database (3ie)	No	Full	None
	National Criminal Justice Reference Service	No	Full	None
	Safety Lit Database	No	Full	None
	Australian Institute of Criminology	No	Full	None
	Bureau of Police Research and Development (India)	No	Full	None
	Canadian Police Research Catalogue	No	Full	None
	Centre for Problem‐Oriented Policing	No	Full	None
	College of Policing (including POLKA and Crime Reduction Toolkit)	No	Full	None
	European Police College (CEPOL)	No	Full	None
	Evidence for Policy and Practice Information and Coordinating Centre	No	Full	None
	National Research Institute of Police Science (Japanese)	No	Full	None
	Office of Community Oriented Policing Services	No	Full	None
	Police Executive Research Forum (US)	No	Full	None
	Police Foundation (US)	No	Full	None
	Tasmania Institute of Law Enforcement Studies (Australia)	No	Full	None
	Policing Online Information System (POLIS, Europe)	No	Full	None
	Scottish Institute for Policing Research	No	Full	None
	Centre of Excellence in Policing and Security (Australian, now archived)	No	Full	None

## APPENDIX C GPD SYSTEMATIC COMPILATION STRATEGY

### Inclusion Criteria

Each record captured by the GPD systematic search must satisfy all inclusion criteria to be included in the GPD: timeframe, intervention and research design. There are no restrictions applied to the types of outcomes, participants, settings or languages considered eligible for inclusion in the GPD.

#### Types of interventions

Each document must contain an impact evaluation of a policing intervention. Policing interventions are defined as some kind of a strategy, program, technique, approach, activity, campaign, training, directive, or funding/organizational change that involves police in some way (other agencies or organizations can be involved). Police involvement is broadly defined as:

- Police initiation, development or leadership
- Police are recipients of the intervention or the intervention is related, focused or targeted to police practices
- Delivery or implementation of the intervention by police

#### Types of study designs

The GPD includes quantitative impact evaluations of policing interventions that utilize randomized experimental (e.g., RCTs) or quasi‐experimental evaluation designs with a valid comparison group that does not receive the intervention. The GPD includes designs where the comparison group receives “business‐as‐usual” policing, no intervention or an alternative intervention (treatment‐treatment designs).

The specific list of research designs included in the GPD are as follows:

- Systematic reviews with or without meta‐analyses
- Cross‐over designs
- Cost‐benefit analyses
- Regression discontinuity designs
- Designs using multivariate controls (e.g., multiple regression)
- Matched control group designs with or without preintervention baseline measures (propensity or statistically matched)
- Unmatched control group designs with pre–post intervention measures which allow for difference‐in‐difference analysis
- Unmatched control group designs without preintervention measures where the control group has face validity
- Short interrupted time‐series designs with control group (less than 25 preintervention and 25 postintervention observations)
- Long interrupted time‐series designs with or without a control group (≥25 pre‐ and postintervention observations)
- Raw unadjusted correlational designs where the variation in the level of the intervention is compared with the variation in the level of the outcome

The GPD excludes single group designs with pre‐ and postintervention measures as these designs are highly subject to bias and threats to internal validity.

### Systematic Screening

To establish eligibility, records captured by the GPD search progress through a series of systematic stages which are summarized in Figure C1, with additional detail provided in the following subsections.

All research staff working on the GPD undergo standardized training before beginning work within any of the stages detailed below. Staff then complete short training simulations to enable an assessment of their understanding of the GPD protocols and highlight any areas for additional training. In addition, random samples of each staff's work are regularly cross‐checked to ensure adherence to protocols. Disagreements about screening decisions between staff are mediated by either the project manager or GPD chief investigators.

#### Title and abstract screening

After removing duplicates, the title and abstract of records captured by the GPD systematic search is screened by trained research staff to identify potentially eligible research that satisfies the following criteria:

- Document is dated between 1950 and present
- Document is unique (i.e., not a duplicate)
- Document is about police or policing
- Document is an eligible document type (e.g., not a book review)

Records are excluded if the answer to any one of the criteria is unambiguously “No,” and will be classified as potentially eligible otherwise. Records classified as eligible at the title and abstract screening stage progress to full‐text document retrieval and screening stages.

#### Full‐text eligibility screening

Wherever possible, a full‐text electronic version of an eligible record is imported into *SysReview* (review management software; Higginson & Neville, 2014). For records without an electronic version, a hardcopy of the record is located to enable full‐text eligibility screening. The full‐text of each document is screened to identify studies that satisfy the following criteria:

- Document is dated between 1950 and present
- Document is unique
- Document reports a quantitative statistical comparison
- Document reports on policing evaluation
- Document reports in a quantitative impact evaluation of a policing intervention
- Evaluation uses an eligible research design

## APPENDIX D LIST OF POP EXPERTS CONTACTED

List of policing scholars and practitioners contacted to identify any studies we missed (Note: Job titles reflect employer as of January 2020)

**Table:**

Name	Employer
Bayley, David	University at Albany, State University of New York
Boba Santos, Rachel	Radford University
Bobo, Lawrence	Harvard University
Braga, Anthony	Northeastern University
Bynum, Tim	Michigan State University
Capowich, George	Loyola University, New Orleans
Clarke, Ronald	Rutgers‐Newark, The State University of New Jersey
Cordner, Gary	Kutztown University of Pennsylvania
Davis, Rob	National Police Foundation
Forst, Brian	American University
Glensor, Ron	Arizona State University
Goldstein, Herman	University of Wisconsin Law School
Greene, Jack	Northeastern University
Groff, Elizabeth	Temple University
Hope, Tim	University of Salford
Kennedy, David	John Jay College of Criminal Justice
Klinger, David A.	University of Missouri‐ St. Louis
Knutsson, Johannes	Norwegian Police University College
Koper, Chris	George Mason University
Lauritsen, Janet	University of Missouri‐ St. Louis
Laycock, Gloria	Jill Dando Institute, University College London
Lum, Cynthia	George Mason University
Maclin, Tracey	Boston University Law School
Maguire, Ed	Arizona State University
Manning, Peter	Northeastern University
Mastrofski, Stephen	George Mason University
Mazerolle, Lorraine	University of Queensland, Australia
McGarrell, Ed	Michigan State University
Meares, Tracey	Yale University Law School
Mills, Andy	Santa Cruz Police Department
Moore, Mark	Harvard University
Newman, Graeme	University at Albany, State University of New York
Peterson, Ruth	Ohio State University
Ratcliffe, Jerry	Temple University
Ready, Justin	Griffiths University
Roehl, Janice	Justice Research Center
Rosenbaum, Dennis	University of Illinois at Chicago
Sampson, Rana	Union Bank, San Diego
Saville, Gregory	AlterNation Consulting
Schmerler, Karin	San Diego County District Attorney's Office
Schultze, Phyllis	Rutgers‐Newark, The State University of New Jersey
Scott, Michael	Arizona State University
Sharp, Elaine B.	University of Kansas
Sherman, Lawrence	University of Maryland
Silverman, Eli	John Jay College of Criminal Justice
Skogan, Wesley	Northwestern University
Skolnick, Jerome	New York University Law School
Sousa, William	University of Nevada, Las Vegas
Spelman, William	University of Texas
Stephens, Darrel	Darrel Stephens Group, LLC
Stephenson, Paul	Embrace Child Victims of Crime
Tilley, Nick	Nottingham Trent University
Tita, George	University of California, Irvine
Travis, Jeremy	Laura and John Arnold Foundation
Uchida, Craig	Justice and Security Strategies
Walker, Samuel	University of Nebraska, Omaha
Weisel, Deborah Lamm	North Carolina State University
Wellford, Charles	University of Maryland
Welsh, Brandon	Northeastern University
Willis, James	George Mason University
Worden, Robert	University at Albany, State University of New York

## APPENDIX E CODING SHEET

**POP META ANALYSIS CODING SHEET**

**Reference Information**

1. Document ID: __ __ __ __
2. Study author(s): ____________________

3a. Are multiple publications/reports associated with this intervention?
  1. Yes
  2. No

3b. If yes, list secondary/additional study author(s):___________

3c. Study title(s) associated with intervention: _______________________

4a. Primary publication type(s): ______
  1. Book
  2. Book chapter
  3. Journal article (peer reviewed)
  4. Thesis or doctoral dissertation
  5. Government report (state/local)
  6. Government report (federal)
  7. Police department report
  8. Technical report
  9. Conference paper
  10. Award submission
  11. Other (specify)

4b. Specify (Other)_____________________

5. Publication date (year): ______________

6a. Journal Name: ____________________

6b. Journal Volume: _______________

6c. Journal Issue: ____________

7. Date range of research (when research was conducted):
  Start: ____________
  Finish: ____________

8. Source of funding for study: ___________________

9. Country of publication: ___________________

10. Date coded: ___________

11. Coder's Initials: __ __ __

**Describing the Problem(s)**

12a. How did the problem(s) come to the attention of the police? (Select all that apply)
  1. Crime analysis unit
  2. Citizen meeting/organization
  3. Officer observation/suggestion
  4. Other government agency
  5. Funding agency
  6. Researcher
  7. Other (specify)

12b. Specify (Other) _____________

13. What was the environment where the problem(s) occurred? (Select all that apply)
  1. Residential
  2. Recreational (bars, restaurants, parks)
  3. Hotels/Motels
  4. Offices
  5. Retail
  6. Industrial
  7. Agricultural
  8. Education
  9. Human service (jails, courts, hospitals)
  10. Public ways
  11. Transport (buses, airports)
  12. Open/transitional (construction sites, abandoned buildings)
  13. Citywide/no particular environment specified

14a. What type of event(s) make up the problem(s)? (Select all that apply)______
  1. Predatory crimes against persons (sexual assault, robbery, homicide)
  2. Predatory crimes against property (vandalism, auto theft)
  3. Illegal service crimes (prostitution, selling drugs)
  4. Public disorder crimes (disorderly conduct, drunkenness)
  5. Vehicular/traffic offenses
  6. Status crimes
  7. Hard drug use
  8. White collar crime (forgery, embezzlement etc.)
  9. Overall crime/disorder
  10. Other (specify)

14b. Specify (Other) ___________

15. Specifically, what event(s) makes up the problem(s)? ______________________________________________________________________________

16. The events making up the problem(s) primarily center on which part of the problem analysis/crime triangle?
  1. Offenders
  2. Victims/targets
  3. Guardians or managers
  4. Places/geographic areas

17a. What data sources were used for analysis of the selected problem? (Select all that apply)
  1. Official crime data
  2. Arrest information
  3. Surveys of people (non‐offenders)
  4. Surveys of places or environments
  5. Interviews and discussions with people (non‐offenders)
  6. Interviews of offenders
  7. Literature examination
  8. Consultation with government agencies
  9. Consultations with businesses
  10. Consultations with community organizations
  11. Consultations with community members
  12. Other (specify)

17b. Specify (Other)___________________

18. What was the level/intensity of problem analysis?
  1. No analysis
  2. Shallow or cursory analysis (looked at official data)
  3. Moderate analysis (looked at official data with analysis by time of day, day of week etc.)
  4. In‐depth analysis (3 above, as well as other problem analysis with other data)
  5. Authors do not provide sufficient detail to make an assessment

**Describing the Response**

19. At what unit of analysis was the treatment delivered/intervention primarily directed at?
  1. Micro place (e.g., hot spot)
  2. Meso area (e.g., neighborhoods)
  3. Large area (e.g., entire city)
  4. Individual offender
  5. Individual victim
  6. Group of offenders (e.g., gang)
  7. Group of victims
  8. Individual guardian or manager
  9. Group of guardians or managers
  10. Entire population (no types of individuals or groups specified)
  11. Other (specify)

20a. Did the evaluation use the same unit of analysis as the unit the intervention was directed at?
  1. Yes
  2. No

20b. If No, specify the unit of analysis for the evaluation
  1. Micro place (e.g., hot spot)
  2. Meso area (e.g., neighborhoods)
  3. Large area (e.g., entire city)
  4. Individual offender
  5. Individual victim
  6. Group of offenders (e.g., gang)
  7. Group of victims
  8. Individual guardian
  9. Group of guardians
  10. Entire population (no types of individuals or groups specified)
  11. Other (specify)

21. Briefly describe the response(s) implemented

____________________________________________________________________________________________________________________________________________________________

22a. What techniques of situational crime prevention were used in the implementation of the response? (Select all that apply)
  1. Increasing the effort of crime (target hardening)
  2. Increasing the risks of crime
  3. Reducing the rewards of crime
  4. Reducing provocations
  5. Removing excuses for crime
  6. Situational crime prevention used, but specific techniques not specified
  7. Unclear/Not mentioned (cannot be sure if SCP was used or not)
  8. N/A‐ Situational crime prevention not used
  9. Other

22b. Specify (Other)___________________

23a. What groups (other than the police) were involved in the implementation of the response? (Select all that apply)
  1. Neighborhood associations/organizations
  2. Government organizations/agencies
  3. Social service agencies
  4. Commercial establishments/businesses
  5. National organizations with an interest in the problem (e.g. MADD)
  6. Schools/Academic organizations
  7. Individual residents
  8. Other police agencies
  9. Other criminal justice agencies
  10. Other (specify)

23b. Specify (Other)___________________

24a. At what level of the police department was the response implemented? _____
  1. Department wide
  2. Multiple precincts or sectors
  3. One precinct, sector, or district involved
  4. Special units (i.e. community policing unit) involved
  5. Select few officers in specific area involved
  6. Other (specify)
  7. N/A (not mentioned)

24b. Specify (Other)___________________

***Implementation of Response***

25. What did the evaluation indicate about the implementation of the response? _____
  1. There were no reported implementation issues
  2. There were minor implementation issues
  3. There were more substantial implementation issues
  4. There were major implementation issues/the project was not implemented as planned
  5. Unclear/no process evaluation included

26. If the process evaluation indicated there were problems with implementation of the response, describe these problems:

__________________________________________________________________________________________________________________________________________________________________________________________________________________________________________

***Location of the intervention***

27. Country where study was conducted: __________________

28. City (and state/province, if applicable) where study was conducted: _________________

*The following questions refer to the area receiving treatment*:

29a. Geographic area receiving treatment: ______
  1. Micro place (street segments/blocks)
  2. Neighborhood/police beat
  3. Police district/precinct
  4. Entire city
  5. Other (specify)

29b. Specify (Other)___________________

30. What is the exact geographic area receiving treatment? _____________________________

*The following refer to the area not receiving treatment*

31a. Geographic area NOT receiving treatment: ______
  1. Micro place (street segments/blocks)
  2. Neighborhood/police beat
  3. Police district/precinct
  4. Entire city
  5. Other (specify)

31b. Specify (Other)___________________

32. What is the exact geographic area not receiving treatment? ___________________________

**Methodology/Research design:**

33a. Length of pre‐intervention study period______

33b. Length of intervention study period_________

33c. Length of post‐intervention study period______

34. Is there a secondary time outcome for this intervention? (*Note that for each secondary time outcome, a separate coding sheet is required)*
  1. Yes
  2. No

35a. Type of study: _____
  1. Randomized experiment
  2. Nonequivalent control group (quasi‐experimental)
  3. Multiple time series (quasi‐experimental)
  4. Interrupted time series
  5. Other (specify)

35b. Specify (Other)___________________

35c. If a quasi‐experiment, how was matching of groups achieved?
  1. Propensity score matching
  2. Identification of matching areas or persons through regression analyses
  3. Statistical tests of mean differences among demographic and other relevant variables
  4. Comparison of descriptive statistics with no statistical test of differences across groups
  5. Comparison to the rest of a jurisdiction or population that did not receive the treatment

35d. Specify (Other)___________________

36a. Were any sources of nonequivalence or bias reported or implied in the application of the intervention or its analysis (i.e. threats to internal validity)?
  1. Yes
  2. No

36b. If yes, what sources of nonequivalence or bias were identified? (check all that apply and explain)
  1. Extraneous events or factors occurring during the intervention period; historical artifacts
  2. Selection of treatment area based on high baseline crime rate
  3. Measurement confounds (measure changes over time)
  4. Differential attrition, breakdown of randomization, or contamination of control group
  5. Pre‐test analyses indicated nonequivalence between treatment and control groups
  6. Statistical analyses failed to adjust for nonequivalence at baseline
  7. Inappropriate statistical analysis for design
  8. Any outcomes measured by reporters that did not have corresponding outcome measures in the results
  9. Other threats to internal validity (specify)

36c. Explain any yes responses checked in 34b.

______________________________________________________________________________________________________________________________________________________________________________________________________

37. Did the researcher assess the quality of the data collected?
  1. Yes
  2. No

38a. Did the researcher(s) express any concerns over the quality of the data?
  1. Yes
  2. No

38b. If yes, explain ____________________________________________________________________________________________________________________________________________________________

39a. Does the evaluation data correspond to the initially stated problem? (i.e. if the problem is fear of crime, does the evaluation data look at whether fear of crime decreased)
  1. Yes
  2. No
  3. Mixed (i.e. varies across sites or analyses)

39b. If no, explain the discrepancy: ____________________________________________________________________________________________________________________________________________________________

***Outcomes reported** (Note that for each outcome, a separate coding sheet is required)*

40. How many crime/disorder outcomes are reported in the study? ____

41. What is the specific outcome recorded on this coding sheet?

_______________________________________________________________

42. Was it the primary outcome of the study? _______
  1. Yes
  2. No
  3. Can't tell/researcher did not prioritize outcomes

***Dependent Variable***

43a. What type of data was used to measure the outcome covered on this coding sheet? ____
  1. Official data (from the police)
  2. Researcher observations
  3. Self‐report surveys
  4. Other (specify)

43b. Specify (Other)___________________

44a. If official data was used, what specific type(s) of data were used? (Select all that apply)
  1. Calls for service (911 calls)
  2. Arrests
  3. Incident reports
  4. Level of citizen complaints
  5. Other (specify)
  6. N/A (official data not used)

44b. Specify (Other)___________________

45a. If researcher observations were used, what types of observations were taken? (Select all that apply)
  1. Physical observations (e.g. observed urban blight, such as trash, graffiti)
  2. Social observations (e.g. observed disorder, such as loitering, public drinking)
  3. Other observations (specify)
  4. N/A (researcher observations not used)

45b. Specify (Other)___________________

46a. If self‐report surveys were used, who was surveyed? (Select all that apply)
  1. Residents/community members
  2. Business owners
  3. Elected officials
  4. Government/social service agencies
  5. Other (specify)
  6. N/A (self‐report surveys not used)

46b. Specify (Other)___________________

**Effect size/Reports of statistical significance**

***Sample size***

47. Based on the unit of analysis for this outcome, what is the total sample size in the analysis? ________

48. What is the total sample size of the treatment group (group that receives the response)? _______

49. What is the total sample size of the control group (if applicable)? _____

50a. Was attrition a problem in the analysis for this outcome?
  1. Yes
  2. No

50b. If attrition was a problem, provide details (e. g. how many cases lost and why they were lost).

__________________________________________________________________________________________________________________________________________________________________________________________________________________________________________

51a. What do the sample sizes above refer to?
  1. Crimes
  2. People
  3. Geographic areas
  4. Other (specify)

51b. Specify (other) ________________

***Effect Size Data***

52. Raw difference favors (i.e. shows more success for):
  1. Treatment group
  2. Control group
  3. Neither (exactly equal)

9. Cannot tell (or statistically insignificant report only)

53a. Did a test of statistical significance indicate statistically significant differences between either the control and treatment groups or the pre and post tested treatment group for the current outcome? ____
  1. Yes
  2. No
  3. N/A (no testing completed/reported)

53b. If yes, what was the level of statistical significance for the current outcome
  1. .01
  2. .05
  3. .1
  4. N/A (no p‐value reported)

53c. Type of significance test used for the current outcome
  1. One‐tailed
  2. Two‐tailed

9. Cannot tell (unclear from text or not reported)

54a. Was a standardized effect size reported?
  1. Yes
  2. No

54b. What type of effect size was reported? _______________

55. If yes, what was the effect size? ______

56. If yes, page number where effect size data is found ________

57. If no, is there data available to calculate an effect size?
  1. Yes
  2. No

58a. Type of data effect size can be calculated from:
  1. Means and standard deviations
  2. *t*‐value or *F*‐value
  3. Chi‐square (df=1)
  4. Frequencies or proportions (dichotomous)
  5. Frequencies or proportions (polychotomous)
  6. Other (specify)

58b. Specify (other) _________

*Pre‐post Study Counts*

59a. Pre‐period number of events for current outcome in target area _______

59b. During intervention‐period number of events for current outcome in target area ______

59c. Post‐period number of events for current outcome in target area ______

59d. Pre‐period number of events for current outcome in comparison area _______

59e. During intervention‐period number of events for current outcome in comparison area _____

59f. Post‐period number of events for current outcome in comparison area ______

59g. Did the evaluation control for validity by using multivariate methods (i.e. regression) to assess the impact of the program?
  1. Yes
  2. No

59h. If yes, did this analysis find that the intervention reduced the outcome at a statistically significant level?
  1. Yes
  2. No
  3. N/A

*Means and Standard Deviations*

60a. Pre‐period treatment group mean. _____

60b. During intervention‐period treatment group mean_____

60c. Post‐period treatment group mean_____

60d. Pre‐period control group mean. _____

60e. During intervention‐period control group mean_____

60f. Post‐period control group mean_____

61a. Pre‐period treatment group standard deviation. _____

61b. During intervention‐period treatment group standard deviation. ____

61c. Post‐period treatment group standard deviation. _____

61d. Pre‐period control group standard deviation. _____

61e. During intervention‐period control group standard deviation. _____

61f. Post‐period control group standard deviation. ______

*Proportions or frequencies*

62a. *n* of treatment group with a successful outcome. _____

62b. *n* of control group with a successful outcome. _____

63a. Proportion of treatment group with a successful outcome. _____

63b. Proportion of control group with a successful outcome. _____

64. IRR value _______

*Significance Tests*

65a. *t*‐value _____

65b. *F*‐value _____

65c. Chi‐square value (*df*=1) _____

*Calculated Effect Size*

66a. Effect size ______

66b. Standard error of effect size _____

**Mediation Analysis** *(Note that for each mediator/outcome, a separate coding sheet is required)*

67. Did the evaluation include a mediational analysis?
  1. Yes
  2. No

68a. What is the specific mediator recorded on this coding sheet?

________________________________________________________________________

68b. Coefficient from treatment to mediator ______

69a. What is the outcome of the mediational analysis?

________________________________________________________________________

69b. Coefficient from mediator to outcome______

**Cost‐Benefit Analysis** *(Note that for each outcome, a separate coding sheet is required)*

70a. Did the evaluation include a cost‐benefit analysis?
  1. Yes
  2. No

70b. If yes, what was the primary unit of analysis for the cost benefit evaluation? (Select all that apply)
  1. Police personnel hours spent on problem
  2. Officer time spent attending CFS/problem incidents
  3. Cost associated with CFS/problem incidents
  4. Other/city costs (specify)

70c. Specify (other) ______________

71. What is the specific cost‐benefit outcome captured on this coding sheet? ________________________________________________________________________

72. Cost of implementing response. _______

73a. Pre‐intervention number of hours spent on identified problem. _______

73b. Post‐intervention number of hours spent on identified problem. _______

74a. Average pre‐intervention personnel hours spent on identified problem. _______

74b. Average post‐intervention personnel hours spent on identified problem. _______

75a. Average yearly pre‐intervention cost attributed to CFS/problem incidents. ______

75b. Average yearly post‐intervention cost attributed to CFS/problem incidents. ______

75c. Total pre‐intervention cost attributed to CFS/problem incidents. _______

75d. Total post‐intervention cost attributed to CFS/problem incidents. _______

76a. Police cost associated with single problem incident. ______

76b. Pre‐intervention treatment group cost. ______

76c. Pre‐intervention control group cost. ______

76d. Post‐intervention treatment group cost. ______

76e. Post‐intervention control group cost. _______

*For the following questions please consider the cost of implementing the response (if applicable)*

77a. Treatment group cost savings. _____

77b. Control group cost savings. _____

78. Total departmental cost savings._____

**Conclusions made by the author(s)**

*Note that the following questions refer to conclusions about the effectiveness of the intervention in regards to the current outcome/problem being addressed on this coding sheet*.

79. Conclusion about the impact of the intervention?_____
  1. The authors conclude intervention associated with a crime decline
  2. The authors conclude intervention not associated with a crime decline
  3. Mixed across crime/disorder type
  4. Unclear/no conclusion stated by authors

80. Did the assessment find evidence of a geographic displacement of crime?______
  1. Yes
  2. No
  3. No statistical test but authors claim evidence of displacement
  4. No statistical test but authors claim no evidence of displacement
  5. Mixed across crime/disorder type
  6. Not tested

81a. Did the assessment find evidence of other non‐geographic types of displacement of crime?_____
  1. Yes
  2. No
  3. No statistical test but authors claim evidence of displacement
  4. No statistical test but authors claim no evidence of displacement
  5. Mixed across crime/disorder type
  6. Not tested

81b. If yes, specify what types of displacement were found

____________________________________________________________________________________________________________________________________________________________

82. Additional notes about conclusions:

____________________________________________________________________________________________________________________________________________________________

83. Additional notes about study:

____________________________________________________________________________________________________________________________________________________________

## APPENDIX F LIST OF EXCLUDED STUDIES

**Table:**

Author(s)	Intervention	Location	Reason for exclusion
Anaheim Police Department (2010)	Truancy Reduction Program	Anaheim, CA	No crime/disorder outcome
Arias and Ungar (2009)	Community Policing	Multiple Sites in Central and South America	Community policing focus, no evidence of SARA model being used
Bässmann (2011)	Vehicle Theft Reduction	Germany	No comparison area and timeline of intervention unclear
Braga (2008)	Pulling Levers Intervention	Stockton, CA	“Pulling levers” focus, did not follow SARA model
Braga and Dusseault (2018)	POP Approach to Improving Homicide Clearance Rates	Boston, MA	No crime/disorder outcome
Braga, Pierce, McDevitt, Bond, and Cronin (2008)	Pulling Levers Intervention	Lowell, MA	“Pulling Levers” focus, no evidence of SARA model being used
Cincinnati Police Department (2011)	Traffic Safety Program	Cincinnati, OH	No crime/disorder outcome
Corsaro, Brunson, and McGarrell (2009)	Pulling Levers Intervention	Rockford, IL	“Pulling Levers” focus, no evidence of SARA model being used
Corsaro and McGarrell (2010)	Pulling Levers Intervention	Indianapolis, IN	“Pulling levers” focus, no evidence of SARA model being used
Fagan, Davies, and Holland (2005)	Drug Elimination Program	New York, NY	No evidence of SARA model being used
Fell, Fisher, Yao, and Scott McKnight (2017)	Beverage Enforcement Program	Multiple sites, US	No evidence of SARA model being used
Florence, Shepherd, Brennan, and Simon (2011)	Anonymized Information Sharing Program	Cardiff, Wales (UK)	Not a police‐led intervention, no evidence of SARA model being used
Hampshire Constabulary (2007)	Program to Improve Crime Reporting in Convenience Stores	Portsmouth, Hampshire (UK)	No crime/disorder outcome, intervention not intended to reduce crime
Hyunseok, Hoover, and Joo (2010)	Compstat	Fort Worth, TX	Compstat focus, no evidence of SARA model being used
Koper, Woods, and Isom (2016)	Community Gun Violence Initiative	St. Louis, MO	POP not the main focus, largely a variation on “Pulling Levers”
Kumar (2012)	Community Policing	Kerala, India	No crime/disorder outcomes
Lancashire Constabulary (2010)	Glassware Injury Reduction Project	Lancashire, UK	No crime/disorder outcome
Lancashire Constabulary (2017)	Vulnerable Callers Project	Lancashire, UK	No crime/disorder outcome
Las Vegas Metropolitan Police Department (2008)	Directed Patrol and CCTV Intervention	Las Vegas, NV	Not POP, no evidence of the SARA model being used
LaVigne, Owens, and Hetrick (2009)	Target's Safe City Initiative	Multiple sites, US	Not a police‐led intervention, no evidence of SARA model being used
Lisburn Neighbourhood Team (2009)	Anti‐Social Behavior Reduction Program for Offenders with ADHD	Lisburn, UK	Noncomparable control area
Maguire, Johnson, Kuhns, and Apostolos (2019)	Community Policing	Trinidad and Tobago	Not an evaluation of a particular POP project, mixture of policing tactics and unable to isolate the effects of POP
Martinez (2013)	Team Policing	Las Vegas, NV	Mixture of multiple policing tactics, unable to isolate effects of POP
Mazerolle, Rombouts, and McBroom (2006)	Operational Performance Reviews	Queensland, Australia	Compstat focus, no evidence of SARA model being used
Mazerolle, McBroom, and McBroom (2011)	Compstat	Queensland, Australia	Compstat focus, no evidence of SARA model being used
Mazerolle, Darroch, and White (2013)	Leadership in Problem‐Oriented Policing	South Australia	Evaluation of leadership rather than specific intervention
McGarrell, Chermak, Wilson, and Corsaro (2006)	Pulling Levers Intervention	Indianapolis, IN	“Pulling levers” focus, POP aspect of intervention captured in Nunn et al. (2006)
McGarrell, Corsaro, Hipple, and Bynum (2010)	Project Safe Neighborhoods	Multiple sites, US	Aggregate evaluation, interventions varied
Metropolitan Police Service (2006)	Disorder Reduction Program at Local Estate	London, UK	No comparison area
Norman Police Department (2012)	My Body My Life Female Empowerment Program	Norman, OK	No comparison group
Providence Police Department (2018)	Reducing Street Level Prostitution	Providence, RI	Outcomes do not match intervention, lack of clarity regarding intervention timeline
Rajaee, Madden Rodriguez, Addison, Readio, and Longwood (2013)	Law Enforcement Advocate Program	Denver, CO	No evidence of SARA model being used
Sedelmaier & Hipple (2016)	New Haven Smart Policing Initiative	New Haven, CT	Focus on foot patrol, unable to isolate effects of POP
Sullivan (2013)	School Resource Officers	Multiple sites in Kentucky, US	Not a full test of POP, no evidence of SARA model being used
Wagers (2007)	Broken Windows Policing	Los Angeles, CA	No evidence SARA model was used
Washington State Patrol (2006)	Ticketing Aggressive Cars	Washington State, US	No crime/disorder outcome
